# Photoorganocatalytic trifluoromethylation of (het)arenes in green conditions

**DOI:** 10.3762/bjoc.22.50

**Published:** 2026-04-30

**Authors:** Egor N Boronin, Svetlana E Kaurkina, Milena M Svetlakova, Anton S Bolshakov, Maxim V Arsenyev, Vasilii F Otvagin, Alexey Yu Fedorov, Timothy Noël, Alexander V Nyuchev

**Affiliations:** 1 Department of Organic Chemistry, N.I. Lobachevsky State University of Nizhny Novgorod, Gagarina Av. 23, 603950 Nizhny Novgorod, Russia; 2 Van't Hoff Institute for Molecular Sciences, University of Amsterdam, Science Park 904, 1098 XH, Amsterdam, The Netherlands

**Keywords:** green chemistry, photocatalysis, photochemistry, trifluoromethylation

## Abstract

A sustainable photocatalytic protocol for the trifluoromethylation of (hetero)arenes is reported. The method operates under metal- and base-free conditions using an inexpensive and atom-efficient CF_3_ source, trifluoroacetic anhydride, in ethyl acetate as a green solvent. An organic cyanoarene photocatalyst enables efficient CF_3_ radical generation under blue-light irradiation, providing a broad range of trifluoromethylated arenes and heteroarenes. The reaction displays pronounced sensitivity to substituent patterns rather than electronic effects. Mechanistic investigations, including radical trapping, Stern–Volmer analysis, and DFT calculations, support a reductive quenching pathway involving photocatalyst-mediated reduction of TFAA. The protocol is amenable to scale-up, with continuous-flow operation delivering a significant increase in space–time yield, highlighting its potential for sustainable synthesis of CF_3_-containing molecules.

## Introduction

Trifluoromethylated compounds play a crucial role in pharmaceuticals, agrochemicals, and advanced functional materials. A substantial fraction of newly approved drugs feature a CF_3_ group, reflecting its unique ability to modulate the chemical stability, lipophilicity, and biological activity of active pharmaceutical ingredients (APIs) [1–3]. However, current approaches to accessing CF_3_-containing molecules frequently depend on costly and toxic rare metals, as well as on highly specialized trifluoromethyl sources [4–5]. This has stimulated an urgent demand for atom-economical and practical strategies to construct trifluoromethyl-substituted compounds.

Over the past decade, numerous photocatalytic protocols for the trifluoromethylation of arenes and heteroarenes have been developed, including approaches employing microflow techniques [6]. These transformations utilize a broad spectrum of catalysts, ranging from Ru- and Ir-based complexes to other transition metals and organic photocatalysts, as well as diverse CF_3_ sources. The major limitations of these methodologies include the high cost of catalysts and trifluoromethylating reagents, along with the use of toxic species such as transition-metal complexes and hazardous gases, which necessitate rigorous purification of the target products, particularly in the case of APIs.

During the last 15 years, several strategies for efficient trifluoromethylation employing relatively inexpensive CF_3_ sources have been reported. These methodologies are based on UV-driven [7], metal-catalyzed [8], organic photocatalyst-mediated, or electrochemical transformations [9], and also include continuous-flow processes [6,10–13]. Given that photocatalytic reactions align with the principles of green chemistry, particularly energy efficiency and the use of catalytic pathways, such approaches can be regarded as among the most environmentally sustainable methods for trifluoromethylation.

**Scheme 1:**
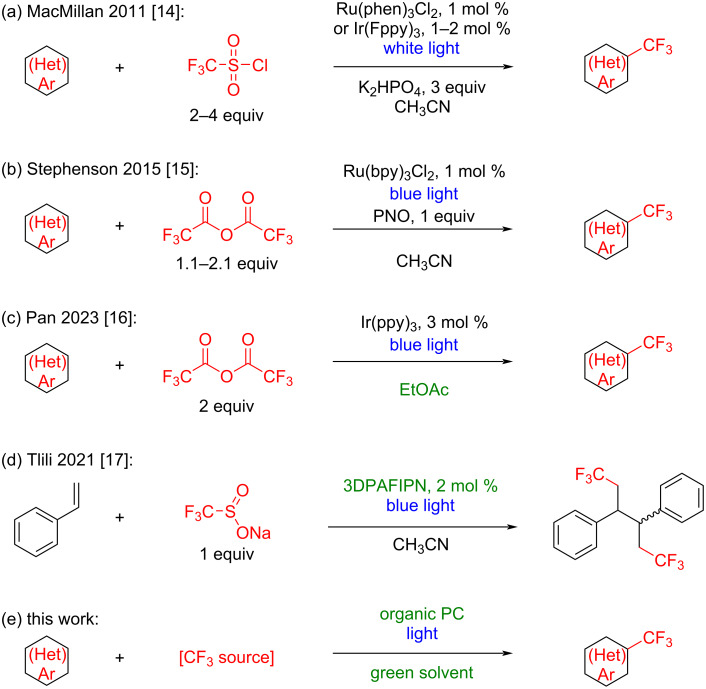
Selected photocatalytic trifluoromethylation procedures.

MacMillan and co-workers reported the photochemical trifluoromethylation of arenes and heteroarenes using Ru- and Ir-based photocatalysts together with trifluoromethanesulfonyl chloride (TfCl) in the presence of a base in acetonitrile (Scheme 1a) [14]. Stephenson and colleagues subsequently demonstrated the use of trifluoroacetic anhydride (TFAA) with a Ru-based photocatalyst in acetonitrile, employing pyridine-*N*-oxide as an additive (Scheme 1b) [15]. More recently, the Pan group developed a protocol for the photochemical trifluoromethylation of (hetero)arenes using an Ir-based photocatalyst under blue-light irradiation (Scheme 1c) [16]. Although these approaches employed inexpensive CF_3_ sources, they relied on costly noble-metal catalysts with loadings of up to 3 mol %. Moreover, all of these transformations were performed in acetonitrile, which reduces their ecological compatibility, particularly for large-scale applications. Tlili and co-workers have described the use of an organic photocatalyst for the CF_3_-functionalization of styrenes with the Langlois reagent (sodium trifluoromethanesulfinate) under blue-light irradiation in acetonitrile (Scheme 1d) [17]. Also, photoelectrochemical trifluoromethylation procedures using trifluoroacetic acid or its salt were published recently [18–19]. Inspired by these methodologies, we sought to combine their advantages to develop a metal- and base-free, atom-economical, and cost-effective photocatalytic protocol for the trifluoromethylation of arenes and heteroarenes in a green solvent (Scheme 1e).

The cost of reagents is one of the key factors in large-scale synthesis. We have updated the price comparison table of trifluoromethylating agents originally reported by Bazyar and Hosseini-Sarvari [20]. A comparison of the atom efficiencies of various CF_3_ sources clearly indicates that trifluoroacetic acid (TFA) is the most atom-efficient and, moreover, the least expensive CF_3_ source (Table 1).

**Table 1:** Comparison of atom efficiency and prices of the CF_3_ sources.^a^

	CF_3_ source	Atom efficiency^b^ (%)	Price^c^ (USD/mol CF_3_)

1	TFA	60.5	41
2	TFAA	32.9^d^	139
3	CF_3_I	35.2	978
4	TfCl	41.0	1948
5	Langlois reagent	44.2	1717
6	Ruppert – Prakash reagent	48.5	2371
7	Umemoto reagent	20.3	56452
8	Togni’s Reagent	20.9	61925

^a^For details see Supporting Information File 1. ^b^Atom efficiency calculated as ratio of M(CF_3_) = 69.01 g/mol to molar weight of corresponding CF_3_-source. ^c^Prices in US dollars per 1 mol are given according to Sigma-Aldrich web site and actual for USA on 6th October, 2025. ^d^In case the only CF_3_-group transfer into product.

Although iodotrifluoromethane, triflyl chloride, and the Langlois and Ruppert–Prakash reagents formally exhibit higher atom efficiencies than trifluoroacetic anhydride (TFAA), they are significantly costlier and challenging to prepare. In contrast, trifluoroacetic acid generated as a by-product can be readily converted back into TFAA, especially in large-scale processes. Umemoto and Togni reagents display low atom efficiencies in addition to very high costs. Taken together, these considerations indicate that TFA and TFAA represent the most attractive choices from both ecological and economic perspectives.

In the selection of solvents for photochemical transformations, acetonitrile is most commonly employed owing to its aprotic nature, compatibility with a wide range of photocatalysts, and transparency to visible light. However, solvent selection guides from Pfizer, GSK, and Sanofi rate acetonitrile as “Usable,” “Major issues,” and “Recommended,” respectively; in contrast, ethyl acetate is assessed as “Preferred,” “Some issues,” and “Recommended” [21]. Importantly, ethyl acetate can be produced from renewable feedstocks, namely, bio-acetic acid and bio-ethanol, both derived from biobased materials. According to the GSK solvent guide, the key drawbacks of acetonitrile are related to waste recycling and biotreatment (2 out of 10), as well as life cycle score (environmental impact of production, 3 out of 10) [22]. By comparison, ethyl acetate receives the lowest ratings for waste treatment and flammability (4 out of 10 for each). Taken together, these data indicate that among the solvents employed in the procedures shown in Scheme 1, ethyl acetate represents a more environmentally sustainable alternative.

Herein, as a continuation of our studies on photochemical approaches to the synthesis of fluorine-containing compounds [23–25] and on the development of methodologies for efficient chemical synthesis [26–27], we report a green and sustainable protocol for the trifluoromethylation of (hetero)arenes under metal- and base-free conditions. The method employs an inexpensive CF_3_ source, an organic photocatalyst, visible light, and a renewable, low-cost solvent.

## Results and Discussion

To identify reaction conditions consistent with the principles of green chemistry, we carried out an optimization study using different organic photocatalysts and solvents. The reactions were performed under an argon atmosphere in anhydrous solvents, employing 1,3,5-trimethoxybenzene (TMB) as the substrate and 3 equivalents of TFAA as the CF_3_ source, over 6 hours at room temperature (25 °C). Initially, white light irradiation was selected owing to its broad coverage of the visible spectrum (Supporting Information File 1, Figure S6). Screening of various organic photocatalysts, including rhodamine B, rhodamine 6G, eosin Y, riboflavin, methylene blue, THPP (tetrahydroxyphenylporphyrin), Birch O-PC™ C0103 (benzo*[ghi]*perylene monoimide) [28], 4CzTPN (tetracarbozalylterephthalonitrile), and 4CzIPN (tetracarbozalylisophthalonitrile), did not afford the desired product (Table 2, entry 1; for detailed results, see Table S3 in Supporting Information File 1).

**Table 2:** Optimization of reaction conditions.

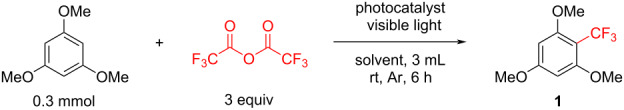				

Entry	Photocatalyst and additives	Solvent	Light^a^	Yield, %^b^

1	photocatalyst, 1 mol %: rhodamine B, rhodamine 6G, eosin Y, riboflavin, methylene blue, THPP, Birch O-PC™ C0103, 4CzTPN or 4CzIPN	EtOAc	white	n.d.
2	4CzIPN, 3 mol %	EtOAc	white	5
3	3DPAFIPN, 1 mol %/2 mol %	EtOAc	white	41/64
4	3DPAFIPN, 1 mol %/2 mol %	EtOAc	violet	50/64
5	3DPAFIPN, 1 mol %/**2 mol %**/5 mol %	EtOAc	blue	54/**78**/87
6	3DPAFIPN, 2 mol %	CHCl_3_, acetone, MeCN, DMF, DMSO or THF	blue	<6
7	3DPAFIPN, 2 mol %	MTBE/BuOAc	blue	27/28
8	3DPAFIPN, 2 mol %	EtOAc	blue	(reaction time, h) 27 (1 h) 46 (2 h) 68 (3 h) 57 (12 h)
9	3DPAFIPN, 2 mol %	EtOAc	blue	(TFAA, equiv) 28 (1 equiv) 47 (2 equiv) 74 (5 equiv) 77 (10 equiv)
10	3DPAFIPN, 2 mol %	EtOAc	blue (50 °C)	68
11	no PC	EtOAc	blue	n.d.
12	3DPAFIPN, 2 mol %	EtOAc	no light	n.d.
13	3DPAFIPN, 2 mol % + TEMPO, 1 equiv	EtOAc	blue	n.d.
14	3DPAFIPN, 2 mol %	EtOAc, in air	blue	n.d.

^a^The light source parameters are described in Supporting Information File 1, Figures S5–S7). ^b^The yield was determined by ^19^F NMR.

Increasing the loading of 4CzIPN to 3 mol % afforded the desired product in 5% yield (Table 1, entry 2). In contrast, another cyanoarene photocatalyst, 3DPAFIPN (tris(diphenylamino)fluoroisophthalonitrile), provided a significantly improved outcome, delivering the product in 41% yield at only 1 mol % catalyst loading (Table 1, entry 3). On this basis, subsequent optimization was performed using 3DPAFIPN as the photocatalyst. Metal-containing catalysts were not used due to their lower environmental compatibility; moreover, they are more expensive compared to cyanoarene derivatives (Table S2, Supporting Information File 1).

Considering the absorbance spectrum of 3DPAFIPN [29], violet (400 nm) and blue (450 nm) LEDs were evaluated as alternative light sources, resulting in increased yields of 50% and 54%, respectively (Table 1, entries 4 and 5).

The influence of catalyst loading was then examined. Raising the photocatalyst concentration to 2 mol % led to yields of 64%, 64%, and 78% under white, violet, and blue light, respectively (Table 1, entries 3–5). A further increase to 5 mol % (Table 1, entry 5) afforded 87% yield; however, this 2.5-fold increase in catalyst loading did not provide a commensurate improvement in efficiency. Moreover, owing to the high molecular weight of 3DPAFIPN, a 5 mol % loading cannot be considered compatible with the development of a green methodology. Consequently, 2 mol % 3DPAFIPN under blue-light irradiation was selected as the optimal set of conditions for further investigations, with subsequent experiments benchmarked against entry 5 (2 mol % PC, 78% yield, Table 1).

We next investigated the effect of solvent on the reaction outcome. Chloroform, acetone, acetonitrile, DMF, DMSO, and THF afforded no more than 6% yield (Table 1, entry 6). Interestingly, MTBE, in contrast to the cyclic ether THF, gave a satisfactory yield of 27% (Table 1, entry 7). BuOAc provided a comparable yield of 28% (Table 1, entry 7), likely due to its higher viscosity (0.685 cP at 25 °C) compared with EtOAc (0.423 cP at 25 °C) [30]. These results established EtOAc as the most suitable solvent for the transformation.

The effect of reaction time was also evaluated. Variation from 1 h to 12 h (Table 1, entry 8) demonstrated that 6 h (Table 1, entry 5) is optimal for achieving the highest yield. Optimization of the CF_3_ source loading showed that 3 equivalents of TFAA (Table 1, entry 5) afforded superior results compared to 1, 2, 5, or 10 equivalents (Table 1, entry 9). Finally, varying the temperature did not lead to further improvements in yield (Table 1, entry 10), in line with observations reported in Pan’s protocol [16].

Finally, a series of control experiments was carried out. No product formation was observed in the absence of catalyst (Table 1, entry 11), in the dark (entry 12), in the presence of TEMPO (entry 13), or under an air atmosphere (entry 14). Based on the optimization studies, the standard reaction conditions were established as follows: 2 mol % 3DPAFIPN in EtOAc under blue-light irradiation (450 nm) at room temperature (23 °C) for 6 h under an argon atmosphere.

**Scheme 2:**
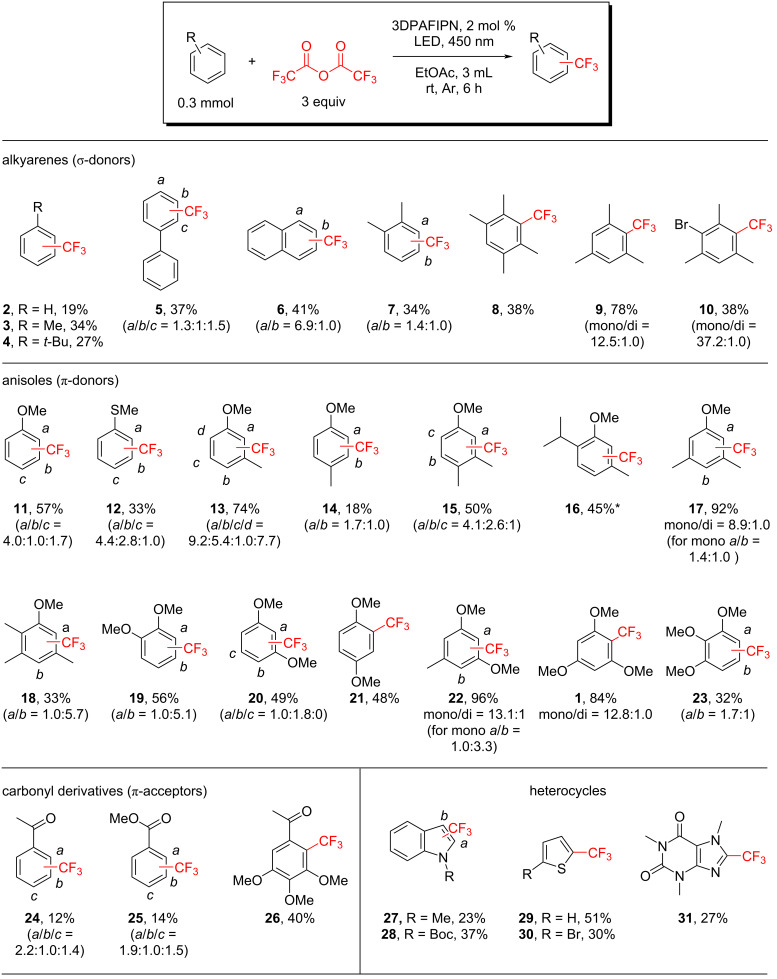
Scope of the trifluoromethylation. Summary yield for all formed isomers is shown for each product. Ratio of isomers is depicted in parentheses. In case of indistinguishable isomers, only their summary yield is shown (depicted as *). Other products and unreactive substrates are shown in Supporting Information File 1.

Using the optimized conditions, the scope of the developed protocol was evaluated with a range of arenes and heteroarenes (Scheme 2); unreactive substrates are shown in Supporting Information File 1, Schemes S4 and S5.

Benzene, toluene, *tert*-butylbenzene, biphenyl, naphthalene, *o*-xylene, and durene afforded the corresponding products **2**–**8** in moderate yields. In contrast, mesitylene, a less electron-enriched substrate than durene, gave product **9** in 78% yield, with traces of bis-trifluoromethylated product. The presence of bromine in the substrate dropped the yield twice, to only 38% for product **10**. Confirming this case, halobenzenes and halotoluenes were obtained with low yields (see Scheme S3 in Supporting Information File 1).

Given that CF_3_^·^ behaves as an electrophilic radical [31–32], it was expected to react preferentially with electron-rich substrates compared to electron-neutral or electron-deficient ones. To probe this trend, trifluoromethylation was examined with substrates bearing donor substituents (methoxyarenes) and acceptors (carbonyl groups).

Trifluoromethylation of anisole afforded product **11** in 57% yield, with the *ortho-*isomer identified as the major product. Compared with anisole, thioanisole, a less electron-rich substrate, produced a mixture of *ortho-*, *meta-*, and *para-*isomers **12** in a combined yield of 33%, with the *meta-*isomer formed in noticeably higher proportion than the *para-*isomer. The introduction of a methyl substituent into the anisole framework significantly altered the reactivity, with a strong dependence on the relative position of the substituents. For the *meta-*substituted substrate, the yield of trifluoromethylated product was high (74% for **13**), whereas the *para-*substituted analogue provided only 18% yield (**14**). To further investigate the influence of substituent position on substrate reactivity, reactions with dialkylanisoles were examined. For *meta-*methylanisoles bearing an additional methyl group at the *ortho-* or *para-*position, only moderate yields were obtained (50% and 45% for **15** and **16**, respectively). When both methyl substituents occupied *meta*-positions, the yield increased markedly to 92% for **17**, with 10% of the product corresponding to the doubly trifluoromethylated derivative. Interestingly, introduction of a third methyl substituent reduced the yield nearly threefold, affording only 33% of product **18**. These observations suggest that the relative arrangement of electron-donating substituents exerts a greater influence on reactivity than their overall donating effect.

Three isomers of dimethoxybenzene gave trifluoromethylated products in modest yields (48–56% for products **19**–**21**). In contrast, the reaction of 3,5-dimethoxytoluene yielded product **22** in an excellent yield of 96%. 1,3,5-Trimethoxybenzene, employed in the optimization studies, delivered product **1** in 84% yield, whereas its isomer, 1,2,3-trimethoxybenzene, provided only 32% yield of product **23**.

To probe the influence of electronic effects, a series of halo-substituted anisoles was examined under the standard conditions. As expected, introduction of a bromine substituent resulted in a pronounced decrease in yield for both the anisoles and methylanisoles (Scheme S3, Supporting Information File 1). Substrates bearing strongly electron-withdrawing substituents and heterocycles furnished products **24**–**30** in low and moderate yields. Finally, caffeine underwent trifluoromethylation at a single position to give product **31** in 27% yield.

### Scaling-up

Based on the optimized conditions, the scalability of the trifluoromethylation of TMB was evaluated (Table 3).

**Table 3:** Scaling-up of the trifluoromethylation of TMB.

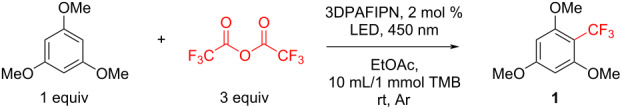						

Entry	Amount of TMB	Reaction vessel	Time	Yield, %^a^	Productivity, mg/h	STY, g L^−1^ h^−1^

1	8 vials × 0.3 mmol (2.4 mmol, 403 mg)	8 vials (i.d. = 11 mm)	6 h	67	63	1.05
2	2.4 mmol (403 mg)	Schlenk flask (i.d. = 30 mm)	6 h	51	48	0.96
3	6.0 mmol (1000 mg)	Schlenk flask (i.d. = 38 mm)	6 h	51	120	1.20
4	50.4 mg (TMB is contained in 1 reactor volume)	Flow setup, **3 mol % PC** (i.d. = 0.75 mm, *V* = 3 mL)	0.5 h	71	101	33.51

^a^The yield was determined by ^19^F NMR.

First, a numbering strategy was applied (Table 3, entry 1), in which eight vials were used to conduct the reaction on a 2.4 mmol scale within the same photoreactor. Under these conditions, the average yield decreased to 67% (compared to 78% under small-scale conditions, Table 2, entry 5). We attribute this decline to a reduced light penetration in each vial, resulting from mutual shading. Subsequently, a Schlenk flask with a larger inner diameter was employed for the same amount of TMB (Table 3, entry 2), which led to an even lower yield of 51% (isolated yield was 39% for entry 2). Further scale-up to a 1 g level, carried out in a Schlenk flask with a 38 mm inner diameter, afforded a comparable yield. In contrast, implementation of a continuous-flow setup provided a higher yield while simultaneously shortening the reaction time. In this case, the catalyst loading was increased to 3 mol % (Table 3, entry 4). As expected, the formal productivity increased with both reactor volume and shorter reaction times, exceeding 100 mg/h for the large-scale flask and the flow setup (Table 3, entries 3 and 4). A comparison of the space–time yield highlights the significant advantage of the flow technique (Table 3, entry 4), which was almost 30-fold higher than that achieved in the most efficient batch reaction (entry 3).

**Scheme 3:**
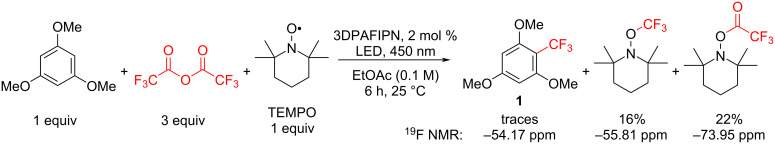
Radical trapping reactions.

Next, we investigated the reaction mechanism. As a first approach, radical trapping experiments were conducted. In line with the results obtained during the optimization, no product formation was observed in the presence of TEMPO or under air (Table 2, entries 13 and 14). To further probe the mechanism, the reaction mixture from the TEMPO-containing experiment was analyzed by q^19^F NMR spectroscopy. Only trace amounts of the trifluoromethylated product were detected, whereas the TEMPO adducts of the trifluoromethyl and trifluoroacetyl radicals were observed in 16% and 22% yield, respectively (Scheme 3).

Stern–Volmer studies of the quenching of the photocatalyst excited state by both TFAA and TMB revealed very similar quenching constants: *K*_q_(TFAA) = 6.07 × 10^9^ M^−1^·s^−1^ and *K*_q_(TMB) = 5.37 × 10^9^ M^−1^·s^−1^ (Figure S16; for further details, see Supporting Information File 1). These results indicate that either initial interaction is feasible under the reaction conditions.

**Scheme 4:**
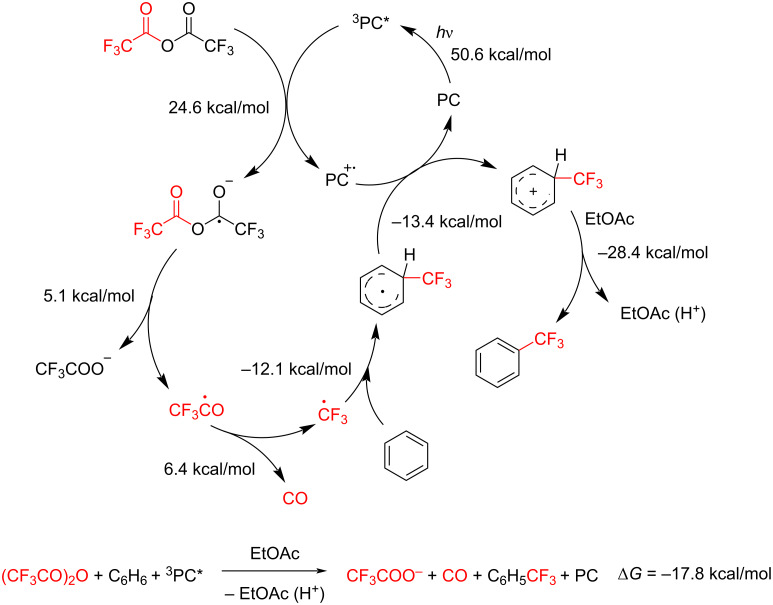
Proposed mechanism.

Since the photocatalyst can function as both an oxidizing and a reducing agent, we examined the literature data to evaluate the possible reaction pathway. Based on the experimentally determined oxidation and reduction potentials of the excited state of 3DPAFIPN (*E*_1/2_(PC^·+^/PC*) = −1.38 V, *E*_1/2_ (PC*/PC^·−^) = 1.09 V) [33], together with the reduction potential of TFAA (*E*_red_ = −1.2 V) [34] and the oxidation potential of TMB (*E*_red_ = 1.43 V) [35], it can be concluded that in our reaction the photocatalyst acts primarily as an electron donor. Accordingly, for the pair of TFAA and TMB, reduction of TFAA is thermodynamically favorable (Δ*E* = 0.18 V), whereas the oxidation of TMB is disfavored (Δ*E* = −0.34 V). We next assessed the theoretical feasibility of the proposed mechanism by means of DFT and TDDFT calculations. The results revealed that the reduction of TFAA, followed by the generation of the CF_3_ radical, is an endothermic process. The subsequent attack of the electrophilic CF_3_ radical on the benzene ring, accompanied by its oxidation, proceeds through exothermic steps. The proton transfer to the solvent constitutes the most favorable step, resulting in an overall free energy change (Δ*G*) of −17.8 kcal mol^−1^ for the catalytic cycle, thereby confirming the energetic feasibility of the proposed mechanism (Scheme 4).

**Scheme 5:**
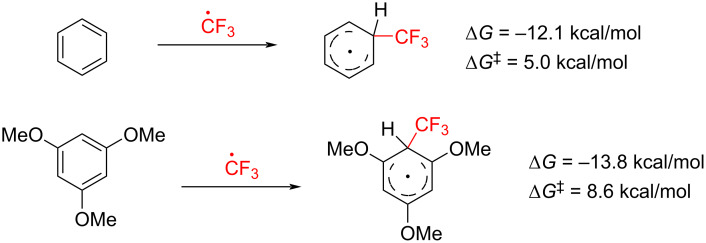
Addition of CF_3_ radical to benzene and TMB.

Furthermore, the scope analysis revealed that trifluoromethylation of benzene proceeds less efficiently than the corresponding reaction with TMB. We therefore hypothesized that the addition of the electrophilic CF_3_ radical to the aromatic ring may differ in energetic profile between the two substrates (Scheme 5).

However, DFT calculations showed that the thermodynamic parameters for both processes are comparable, indicating that other elementary steps may be responsible for this outcome in the case of substituted benzenes (Scheme 5).

## Conclusion

A metal- and base-free photocatalytic trifluoromethylation of (hetero)arenes has been developed using an organic photocatalyst, visible light, and trifluoroacetic anhydride as an inexpensive and atom-efficient CF_3_ source. The reaction proceeds in ethyl acetate, a renewable and environmentally benign solvent, and tolerates a broad range of aromatic substrates. The method is readily scalable via a continuous-flow operation, highlighting its potential for the sustainable synthesis of CF_3_-containing molecules.

## Supporting Information

File 1 Experimental part, copies of NMR spectra and computational data.

## Data Availability

All data that supports the findings of this study is available in the published article and/or the supporting information of this article.

## References

[1] Nair A S, Singh A K, Kumar A, Kumar S, Sukumaran S, Koyiparambath V P, Pappachen L K, Rangarajan T M, Kim H, Mathew B (2022). Processes.

[2] Novás M, Matos M J (2025). Molecules.

[3] Morales‐Salazar I, Islas‐Jácome P, Herrera‐Zuñiga L D, Couve‐Bonnaire S, Jubault P, González‐Zamora E, Bouillon J-P, Islas‐Jácome A (2025). Eur J Org Chem.

[4] Mandal D, Maji S, Pal T, Sinha S K, Maiti D (2022). Chem Commun.

[5] Chen J-Y, Huang J, Sun K, He W-M (2022). Org Chem Front.

[6] Sumii Y, Shibata N (2023). Chem Rec.

[7] Li L, Mu X, Liu W, Wang Y, Mi Z, Li C-J (2016). J Am Chem Soc.

[8] Purushotam, Bera A, Banerjee D (2023). Org Biomol Chem.

[9] Shaw R, Sihag N, Bhartiya H, Yadav M R (2024). Org Chem Front.

[10] Straathof N J W, Gemoets H P L, Wang X, Schouten J C, Hessel V, Noël T (2014). ChemSusChem.

[11] Su Y, Kuijpers K P L, König N, Shang M, Hessel V, Noël T (2016). Chem – Eur J.

[12] Abdiaj I, Bottecchia C, Alcazar J, Nol T (2017). Synthesis.

[13] Beatty J W, Douglas J J, Miller R, McAtee R C, Cole K P, Stephenson C R J (2016). Chem.

[14] Nagib D A, MacMillan D W C (2011). Nature.

[15] Beatty J W, Douglas J J, Cole K P, Stephenson C R J (2015). Nat Commun.

[16] Song Y, Zheng B, Yang S, Li Y, Liu Q, Pan L (2023). Org Lett.

[17] Louvel D, Souibgui A, Taponard A, Rouillon J, ben Mosbah M, Moussaoui Y, Pilet G, Khrouz L, Monnereau C, Vantourout J C (2022). Adv Synth Catal.

[18] Qi J, Xu J, Ang H T, Wang B, Gupta N K, Dubbaka S R, O’Neill P, Mao X, Lum Y, Wu J (2023). J Am Chem Soc.

[19] Chen Y, He Y, Gao Y, Xue J, Qu W, Xuan J, Mo Y (2024). Science.

[20] Bazyar Z, Hosseini-Sarvari M (2019). Org Process Res Dev.

[21] Calvo-Flores F G, Monteagudo-Arrebola M J, Dobado J A, Isac-García J (2018). Top Curr Chem.

[22] Henderson R K, Jiménez-González C, Constable D J C, Alston S R, Inglis G G A, Fisher G, Sherwood J, Binks S P, Curzons A D (2011). Green Chem.

[23] Spennacchio M, Bernús M, Stanić J, Mazzarella D, Colella M, Douglas J J, Boutureira O, Noël T (2024). Science.

[24] Nyuchev A V, Wan T, Cendón B, Sambiagio C, Struijs J J C, Ho M, Gulías M, Wang Y, Noël T (2020). Beilstein J Org Chem.

[25] Govaerts S, Nyuchev A, Noel T (2020). J Flow Chem.

[26] Boronin E N, Svetlakova M M, Vorobyov I I, Malysheva Y B, Polushtaytsev Y V, Mensov S N, Vorotyntsev A V, Fedorov A Y, Noël T, Nyuchev A V (2024). React Chem Eng.

[27] Wan T, Capaldo L, Laudadio G, Nyuchev A V, Rincón J A, García‐Losada P, Mateos C, Frederick M O, Nuño M, Noël T (2021). Angew Chem, Int Ed.

[28] Cole J P, Chen D-F, Kudisch M, Pearson R M, Lim C-H, Miyake G M (2020). J Am Chem Soc.

[29] Zhou C, Lei T, Wei X-Z, Ye C, Liu Z, Chen B, Tung C-H, Wu L-Z (2020). J Am Chem Soc.

[30] Haynes W M (2014). CRC Handbook of Chemistry and Physics.

[31] Duan M, Shao Q, Zhou Q, Baran P S, Houk K N (2024). Nat Commun.

[32] Fernandes A J, Giri R, Houk K N, Katayev D (2024). Angew Chem, Int Ed.

[33] Speckmeier E, Fischer T G, Zeitler K (2018). J Am Chem Soc.

[34] Zhang K, Rombach D, Nötel N Y, Jeschke G, Katayev D (2021). Angew Chem, Int Ed.

[35] Ohkubo K, Mizushima K, Iwata R, Fukuzumi S (2011). Chem Sci.

