# Supplementary material for: Photoorganocatalytic trifluoromethylation of (het)arenes in green conditions
Source: Beilstein J Org Chem. 2026 Apr 30;22:662–71. doi: 10.3762/bjoc.22.50 (PMC13159295; doi:10.3762/bjoc.22.50)
Supplement: File 1 — Experimental part, copies of NMR spectra and computational data. [file Beilstein_J_Org_Chem-22-662-s001.pdf]

BEILSTEIN JOURNAL OF ORGANIC CHEMISTRY

Supporting Information

for

Photoorganocatalytic trifluoromethylation of (het)arenes in green conditions

Egor N. Boronin, Svetlana E. Kaurkina, Milena M. Svetlakova, Anton S. Bolshakov, Maxim V. Arsenyev, Vasilii F. Otvagin, Alexey Yu. Fedorov, Timothy Noël and Alexander V. Nyuchev

Beilstein J. Org. Chem. **2026,** *22,* 662–671. doi:10.3762/bjoc.22.50

Experimental part, copies of NMR spectra and computational data

License and Terms: This is a supporting information file under the terms of the Creative Commons Attribution License (https://creativecommons.org/licenses/by/4.0). Please note that the reuse, redistribution and reproduction in particular requires that the author(s) and source are credited and that individual graphics may be subject to special legal provisions.
The license is subject to the *Beilstein Journal of Organic Chemistry* terms and conditions: (https://www.beilstein-journals.org/bjoc/terms)

Table of contents

1. Materials and methods S2

2. General procedure S7

3. Optimization S10

4. Radical trapping S13

5. Scaling-up S16

6. Photophysical investigations S21

7. Stern–Volmer quenching studies S21

8. Synthesis of starting materials S25

9. Scope and characterization of products S26

10. NMR spectra S57

11. Computational data S104

12. References S120

1. Materials and methods

Commercially available reagents were purchased from Sigma-Aldrich, Alfa Aesar, Abcr, Acros Organics and used without further purification. Other substrates were prepared by using the reported procedures and purified through column chromatography respectively. The catalyst 3DPAFIPN was prepared according to a published procedure [1]. Solvents were purified according to standard procedures. Ethyl acetate (EtOAc) was purified by distillation over phosphorus(V) oxide and stored over molecular sieves 4 Å under an argon atmosphere.

Analytical thin-layer chromatography (TLC) was performed on aluminum plates coated with 0.20 mm silica gel 60 with fluorescent indicator UV_{254} (Macherey-Nagel). Visualization of developed plates was performed under UV light (254 nm, 365 nm) and/or vanillin stains. Column chromatography was performed using Silica 60 (0.063–0.2 mm, Macherey-Nagel).

^{1}H NMR spectra were recorded at 400 MHz (Agilent DD2 400) in $CDCl_3$ (δ = 7.26 ppm) and DMSO-d_6 (δ = 2.50 ppm) and referenced internally to the corresponding residual solvent signals. ^{13}C NMR spectra were recorded at 101 MHz, chemical shifts were reported in ppm on the δ scale relative to $CDCl_3$ (δ = 77.16 ppm). For quantitative ^{19}F NMR, 0.3 mL of resulting solution was transferred to NMR tube and diluted with 0.3 mL of deuterated solvents ($CDCl_3$ or DMSO-d_6). Quantitative q^{19}F NMR spectra were recorded on 4 scans and d1 = 25 s. Chemical shifts are reported in parts per million (ppm) relative to internal standard $C_6H_5CF_3$ (−62.9 ppm in $CDCl_3$ or −62.2 ppm in DMSO-d_6).

Coupling constants are reported in Hz with multiplicities denoted as s (singlet), d (doublet), t (triplet), q (quartet), hept (heptet), m (multiplet).

Fluorescence measurements were acquired at room temperature using a Shimadzu RF-6000 spectrofluorophotometer. The absorbance spectrum of 3DPAFIPN was recorded using a Shimadzu UV-1800 UV–vis spectrophotometer.

GC–MS spectra ware recorded using an QP 2010 plus (Shimadzu) equipped with an Rtx-5MS capillary column (length 60 m; inner diameter 0.32 mm). The injector temperature was set at 250 °C, the detector temperature was 250 °C.

The photochemical reactor consists of a blue diode strip 450 nm (120 diodes/meter SMD 5054 chip, 12 V, 82 klx, total electric power 30–34 W). The reactor supplied a powerful fan (FANOVER, FD9225S12M, 4.8 W, 3000 rpm). 3 m of LED strip were coiled in a 3D-printed (PLA) reactor with an inner diameter of 10 cm [1].

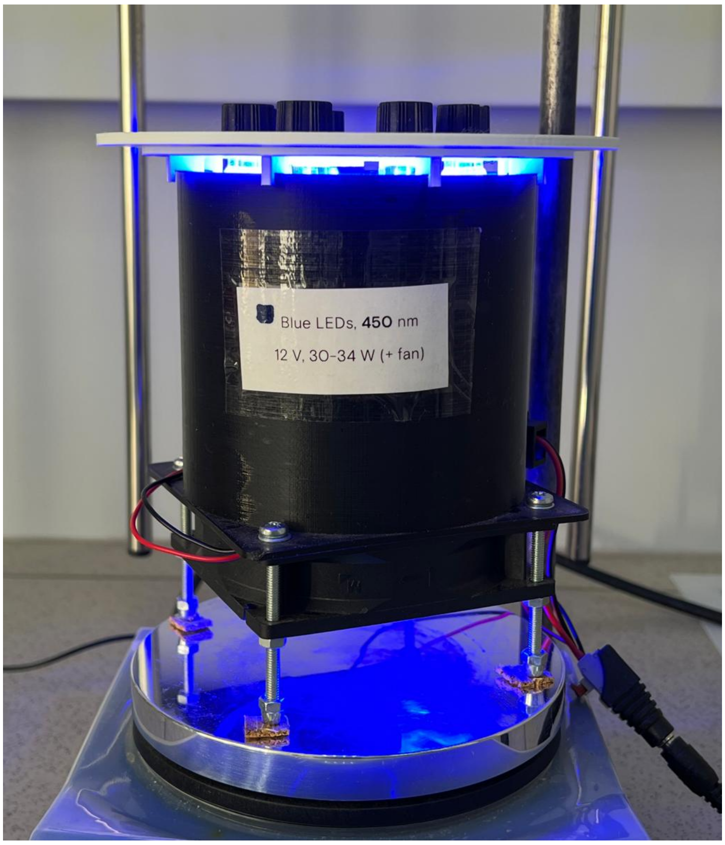

Figure S1. The 3D-printed photoreactor (blue diodes, 450 nm)

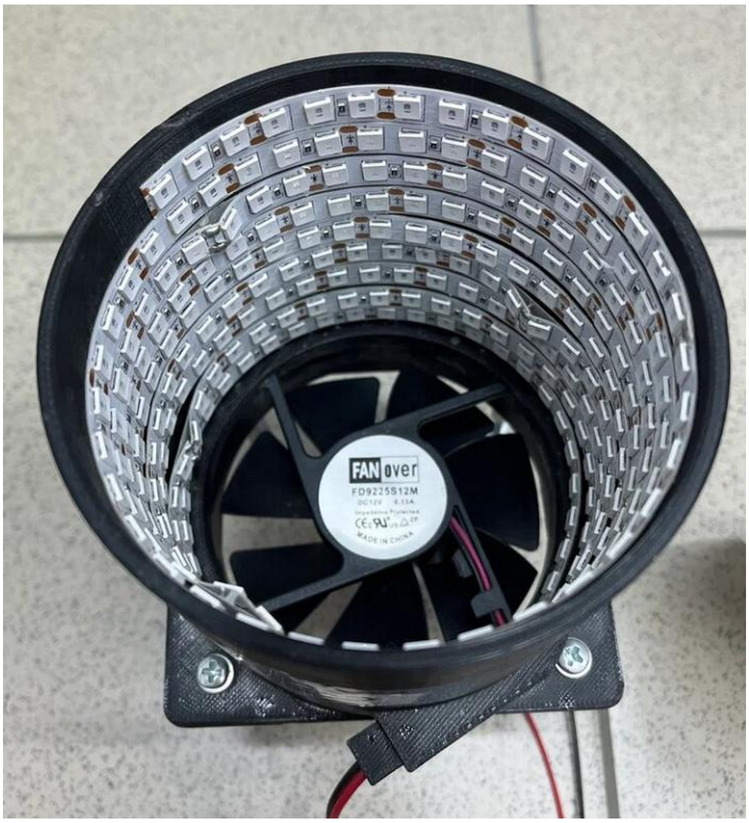

Figure S2. An inside of 3D-printed photoreactor with cooling fan in “off” state

The vial holder (lid) is designed to leave a gap for effective cooling by the air flow from the fan. The temperature inside the reactor (Figure S3) is slightly higher (24.2 °C) than environment temperature (23.5 °C).

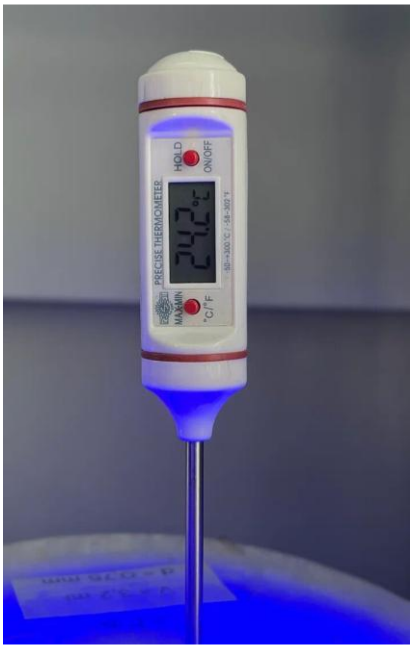

Figure S3. Temperature measurement inside the photoreactor

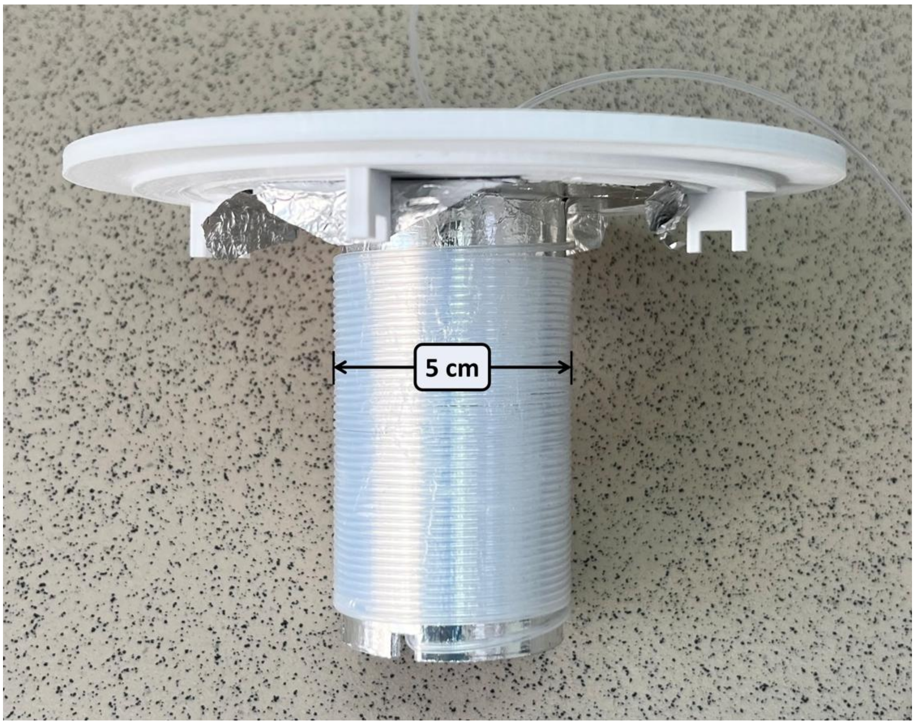

Figure S4. Flow microreactors made of fluorinated ethylene propylene (FEP) tubes (i.d. = 0.75 mm, V = 3.0 mL)

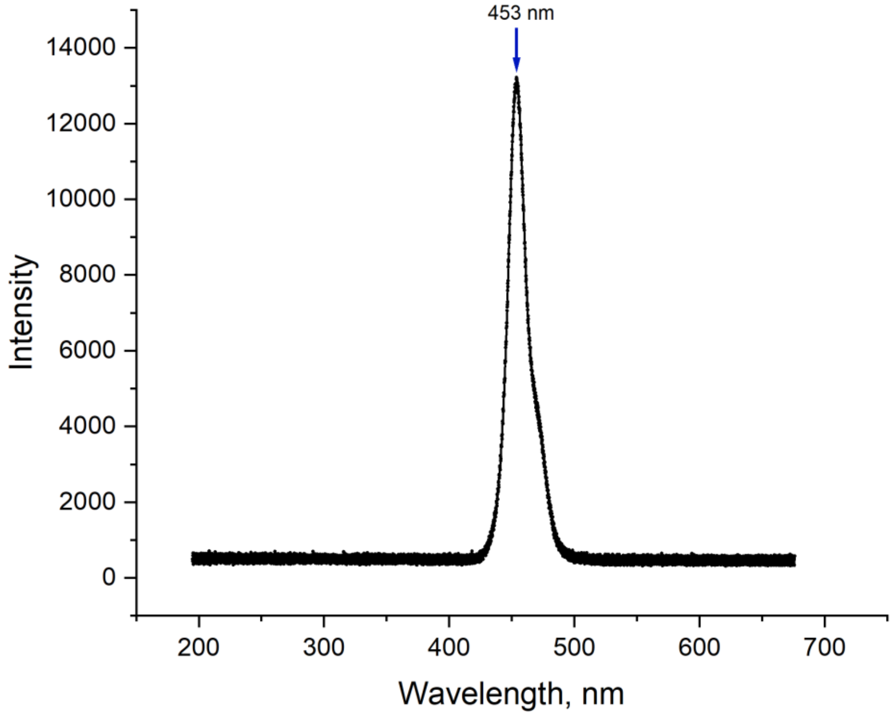

Figure S5. The emission spectrum of the blue LEDs strip (λ_{max}= 453 nm)

Emission spectra of other LEDs:

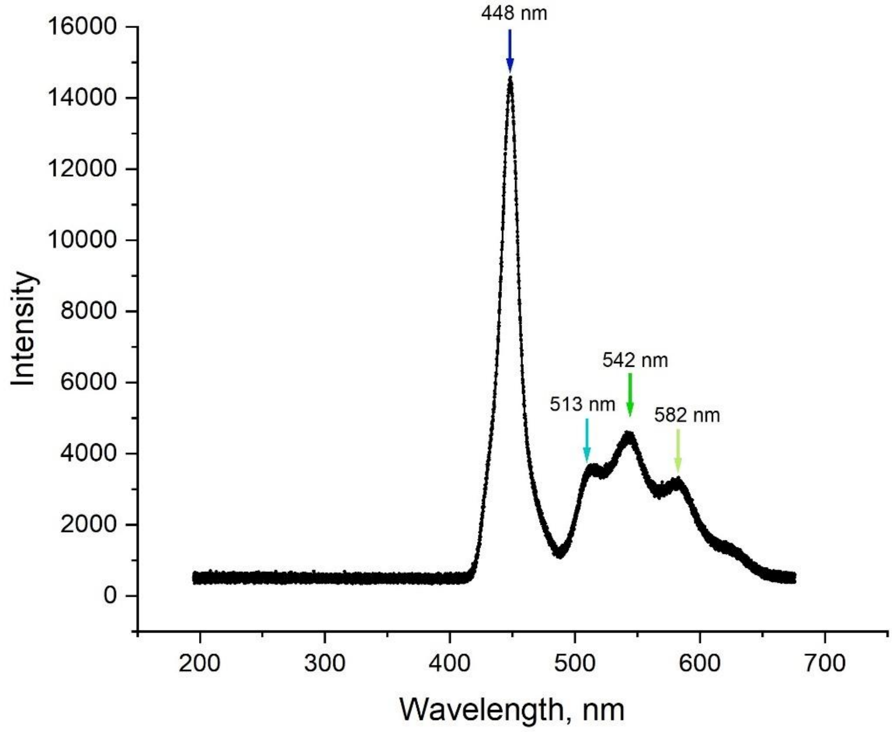

Figure S6. The emission spectrum of the white LEDs strip (λ_{max}= 448 nm)

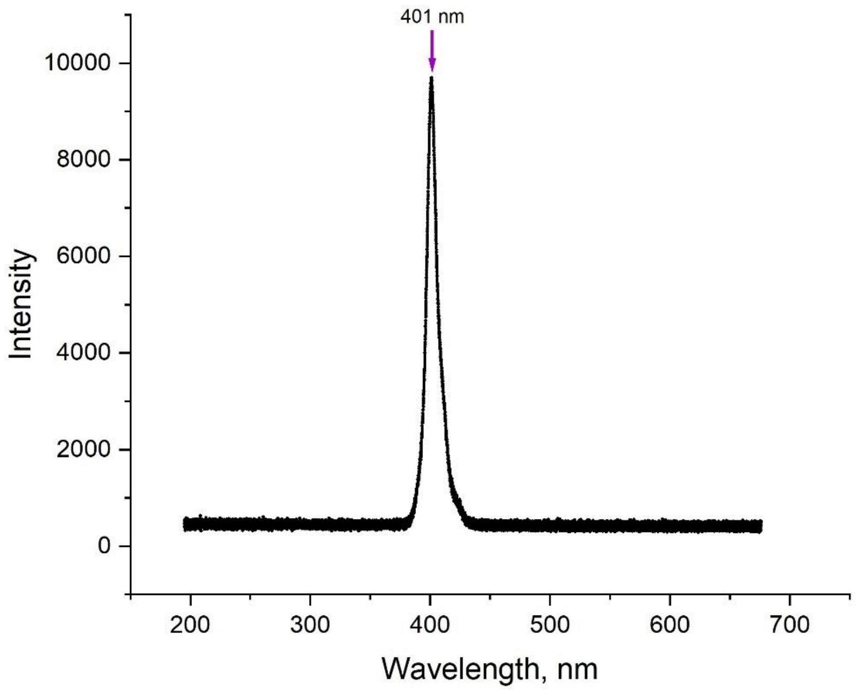

Figure S7. The emission spectrum of the violet LEDs strip (λ_{max}= 401 nm)

Table S1. Comparison of atom efficiency and prices of the CF_3 sources

	CF_3-source	CAS	Product No.	Price		Price# (USD/mol CF_3)	Atom efficiency* (%)
1	TFA	76-05-1	8.0826	539	USD per 1 L	41	60.5
2	TFAA	407-25-0	8.08261	489	USD per 500 mL	139	32.9§
3	CF_3I	2314-97-8	171441	499	USD per 100 g	978	35.2
4	TfCl	421-83-0	164798	289	USD per 25 g	1948	41.0
5	Langlois reagent	2926-29-6	743232	275	USD per 25 g	1717	44.2
6	Ruppert – Prakash reagent ($TMSCF_3$)	81290-20-2	488712	401	USD per 25 mL	2371	48.5
7	Umemoto reagent	131880-16-5	483869	166	USD per 1 g	56452	20.3
8	Togni's Reagent	887144-97-0	696641	938	USD per 5 g	61925	20.9

* – Atom efficiency calculated as ratio of M(CF_3) = 69.01 g/mol to molar weight of corresponding CF_3-source.

# – Prices in US dollars per 1 mol are given according to Sigma-Aldrich web site and actual for USA on 4th February, 2026.

§ – In case the only CF_3-group transfer into product.

Table S2. Comparison of the cost of photocatalysts

	Photocatalyst	CAS	Product No.	Price		Price (USD/mmol)
1	$Ir(ppy)_3$	94928-86-6	688096	443	USD per 250 mg	1160
2	$[Ru(phen)_3]Cl_2$	23570-43-6	904767	227	USD per 250 mg	647
3	3DPAFIPN	2260543-73-3	908177	441	USD per 500 mg	571
4	Tetrafluoroisophthalonitrile (3DPAFIPN precursor)	2377-81-3	CIAH987F07E6	11	USD per 1000 mg	2.2
5	3DPAFIPN (synthesized; taking into account the costs)	2260543-73-3	908177	15	USD per 500 mg	19.4

2. General procedure

For solid substrates: A 7.5 mL Pyrex tube (No. 99447) equipped with a magnetic stirring bar was charged with 3DPAFIPN (0.02 equiv, 0.006 mmol, 3.9 mg) and the solid substrate (1 equiv, 0.3 mmol). Then the atmosphere inside the vial was changed into argon. Dry ethyl acetate (3 mL) was added using a syringe under argon atmosphere via PTFE/silicone septa and then CF_3 reagent TFAA (3 equiv, 0.9 mmol, 125 µL) was added. A stream of argon was bubbled through the reaction mixture for 1 minute in an ultrasonic bath. The reaction vial was placed in the photoreactor zone (blue LEDs, λ = 450 nm) and irradiated for 6 h. After the reaction, 20 µL (23.8 mg, 0.163 mmol) of benzotrifluoride ($C_6H_5CF_3$) was added as standard to the mixture and the mixture stirred for 1 minute. For quantitative ^{19}F NMR 0.3 mL of the resulting solution was transferred to an NMR tube and diluted with 0.3 mL of deuterated solvent (DMSO-d_6 or $CDCl_3$).

For liquid substrates: The liquid substrate was added using a micropipette immediately after dry ethyl acetate. A 7.5 mL Pyrex tube (No. 99447) equipped with a magnetic stirring bar was charged with 3DPAFIPN (0.02 equiv, 0.006 mmol, 3.9 mg). Then the atmosphere inside the vial was changed into argon. Dry ethyl acetate (3 mL) was added using syringe under argon atmosphere via PTFE/silicone septa. Then using a micropipette, the liquid substrate (1 equiv, 0.3 mmol) and CF_3 reagent TFAA (3 equiv, 0.9 mmol, 125 µL) were added. A stream of argon was bubbled through the reaction mixture for 1 minute in an ultrasonic bath. The reaction vial was placed in the photoreactor zone (blue LEDs, λ = 450 nm) and irradiated for 6 h. After the reaction, 20 µL (23.8 mg, 0.163 mmol) of benzotrifluoride ($C_6H_5CF_3$) was added as standard to the mixture and the mixture stirred for 1 minute. For quantitative ^{19}F NMR 0.3 mL of the resulting solution

was transferred to an NMR tube and diluted with 0.3 mL of deuterated solvent (DMSO-d_6 or $CDCl_3$).

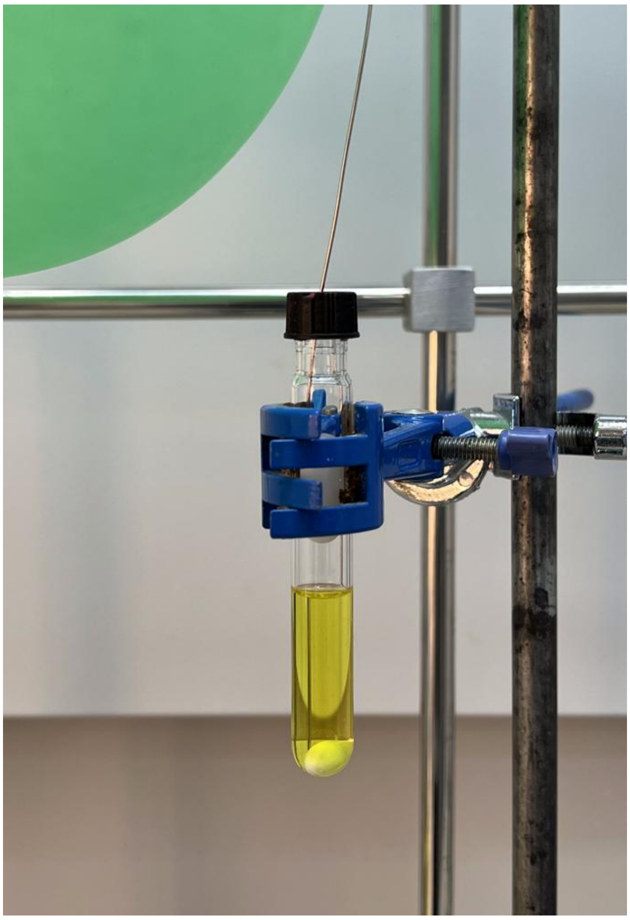

Figure S8. The reaction mixture in a 7.5 mL Pyrex tube

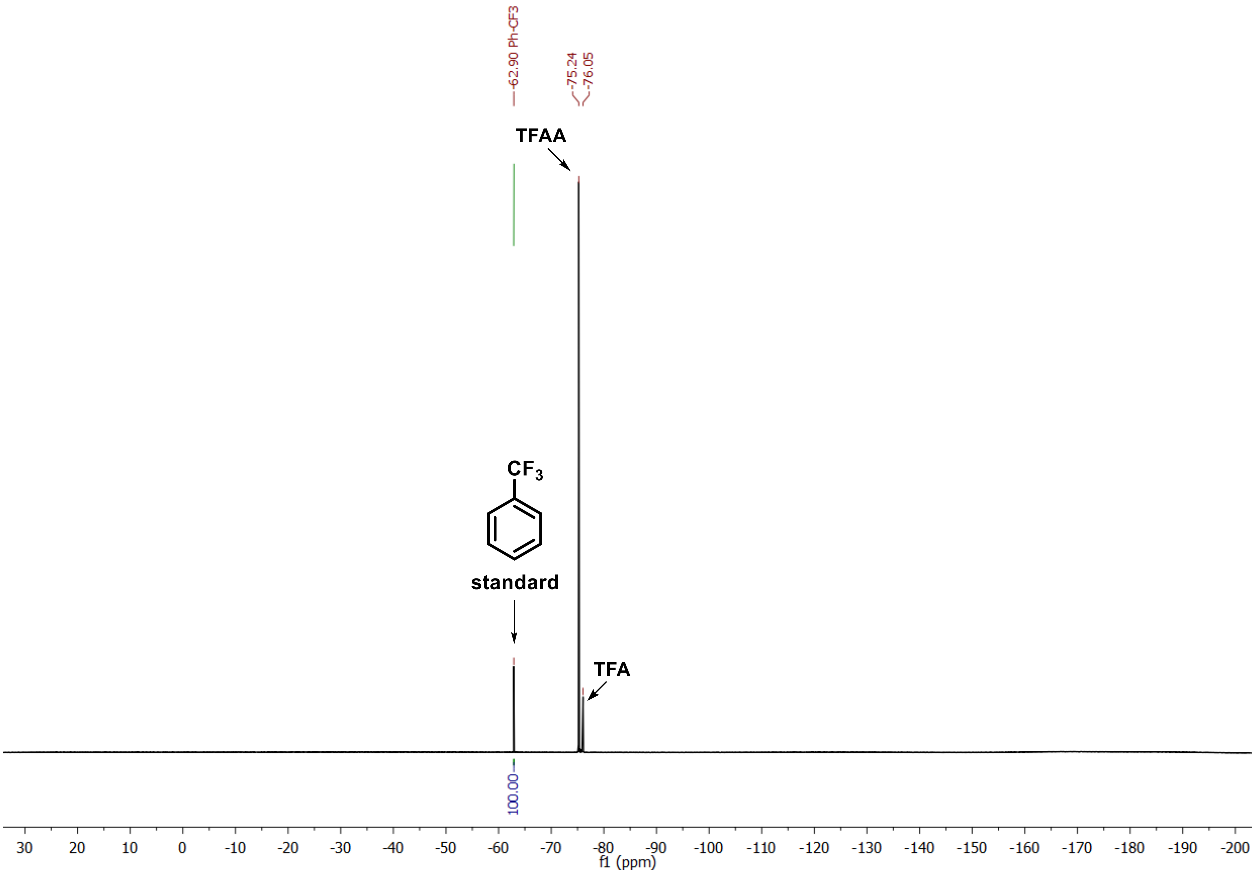

^{19}F NMR spectrum of standard (benzotrifluoride) in $CDCl_3$/EtOAc with TFAA

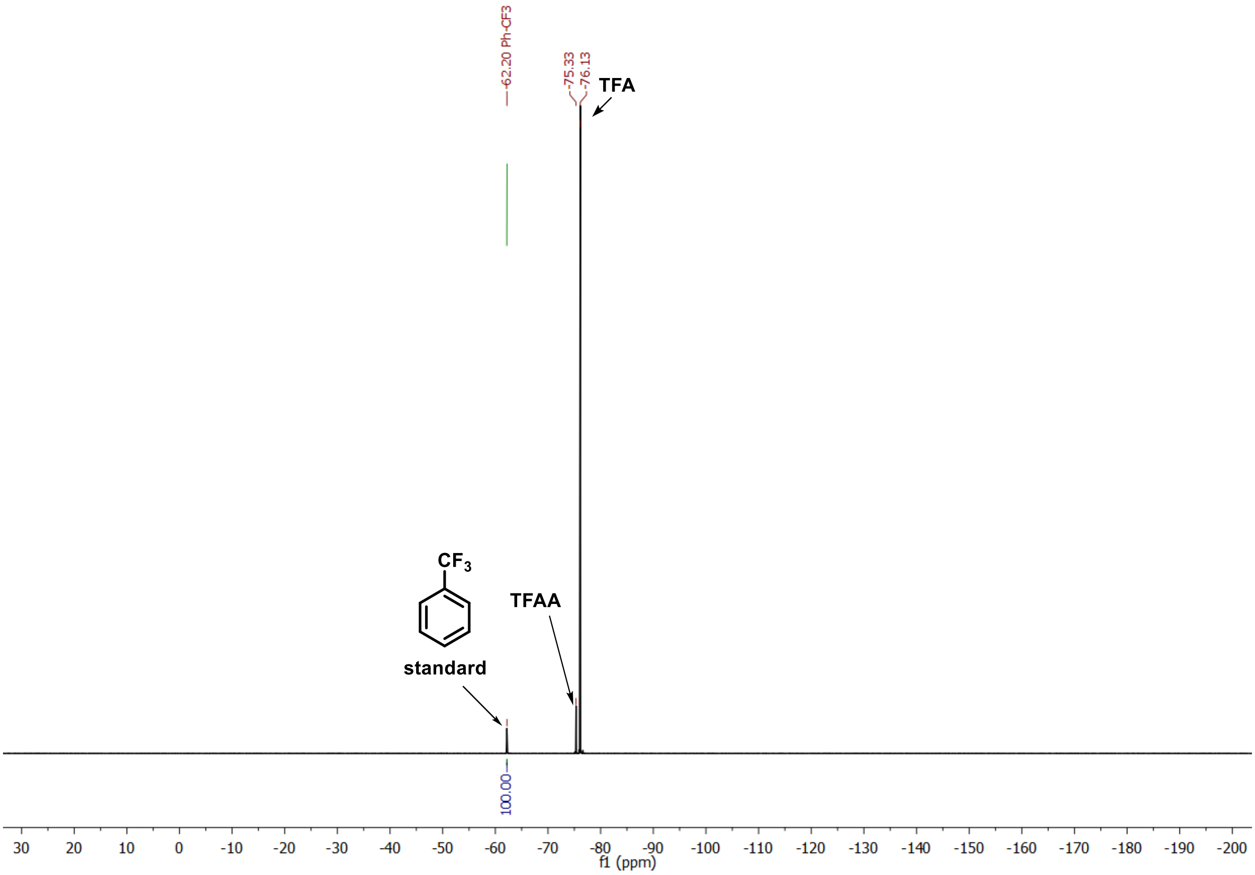

^{19}F NMR spectrum of standard (benzotrifluoride) in DMSO-d_6/EtOAc with TFAA

3. Optimization

Optimal conditions:

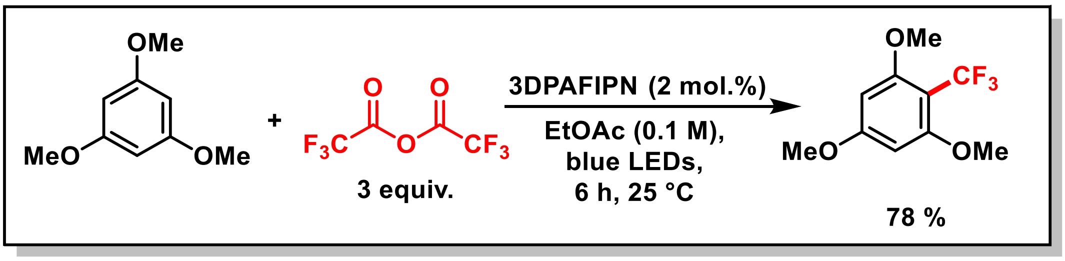

Scheme S1.

Table S3. Screening of organic photocatalysts and light source

Entry	Light source	Photocatalyst	^{19}F NMR yield, %
1	White LEDs	-	-
2		Eosin Y (1 mol.%)	-
3		Rhodamine 6G (1 mol.%)	-
4		Rhodamine B (1 mol.%)	-
5		Birch O-PC™ C0103 (1 mol.%)	-
6		Riboflavin (1 mol.%)	-
7		Methylene blue (1 mol.%)	-
8		4CzTPN (1 mol.%)	-
9		Tetrahydroxyphenyl porphyrin (1 mol.%)	-
10		4CzIPN (1 mol.%)	5
11	White LEDs	3DPAFIPN (1 mol.%)	41
12	Violet LEDs	3DPAFIPN (1 mol.%)	50
13	Blue LEDs	3DPAFIPN (1 mol.%)	54
14	White LEDs	3DPAFIPN (2 mol.%)	64
15	Violet LEDs	3DPAFIPN (2 mol.%)	64
16	Blue LEDs	3DPAFIPN (2 mol.%)	78
17	Blue LEDs	3DPAFIPN (5 mol.%)	87

Conditions: 1,3,5-trimethoxybenzene (0.3 mmol), 3 equiv of TFAA, EtOAc (0.1 M), 6 h, 25 °C

Table S4. Screening of solvents

Entry	Solvent	^{19}F NMR yield, %
1	$CHCl_3$	-
2	DMF	-
3	DMSO	-
4	MeCN	traces
5	Acetone	4
6	THF	6
7	MTBE	27
8	BuOAc	28
**9**	**EtOAc**	**78**

Conditions: 2 mol % 3DPAFIPN, 3 equiv of TFAA, C = 0.1 M, blue LEDs, 6 h, 25 °C

Table S5. Optimization of reaction time

Entry	Time	^{19}F NMR yield, %
1	1 h	27
2	2 h	46
3	4 h	68
**4**	**6 h**	**78**
5	12 h	57

Conditions: 2 mol % 3DPAFIPN, 3 equiv of TFAA, EtOAc (0.1 M), blue LEDs, 25 °C

Table S6. Optimization of amount of TFAA

Entry	Equiv. of TFAA	^{19}F NMR yield, %
1	1	28
2	2	47
**3**	**3**	**78**
4	3 + 1 (after 2 h)	72
5	5	74
6	10	77

Conditions: 2 mol % 3DPAFIPN, EtOAc (0.1 M), blue LEDs, 6 h, 25 °C

Table S7. Various controlled experiments

Entry	Deviations from optimal conditions	Yield, %
1	No PC	-
2	No light	-
3	On air	-
4	C = 0.05 M	15
5	50 ℃	68
6	3 equiv. TFA instead of TFAA	-
7	+ 1 equiv. of TEMPO	-
8	+ 1 equiv. of *para*-quinone	-
9	+ 1 equiv. of Et_3N	2
10	+ 3 equiv. TFA	72

Optimal conditions: 2 mol % 3DPAFIPN, 3 equiv TFAA, EtOAc (0.1 M), blue LEDs, 6 h, 25 °C

4. Radical trapping

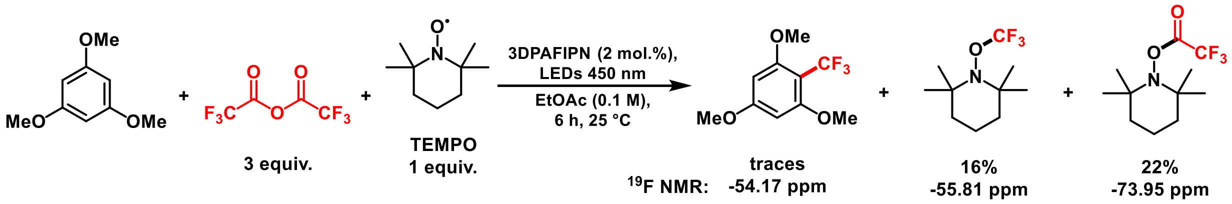

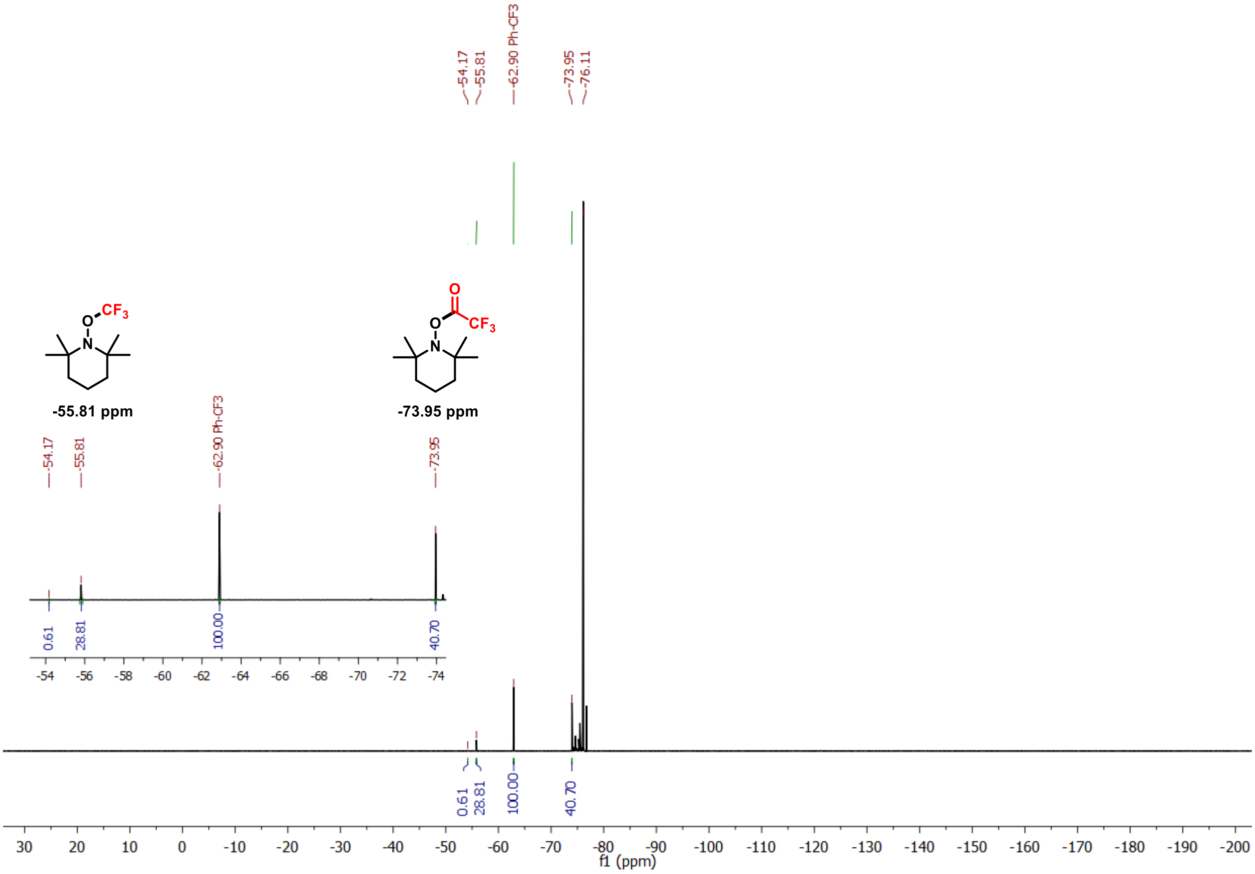

^{19}F NMR spectra of radical trapping with TEMPO

The product **TEMPO–CF$_3$** was further confirmed by GC–MS analysis. **GC–MS** (EI): *m/z* calcd for $C_{10}H_{18}F_3NO$ $[M]^+$ 225; found 225. The product peak was observed at 10.53 min.

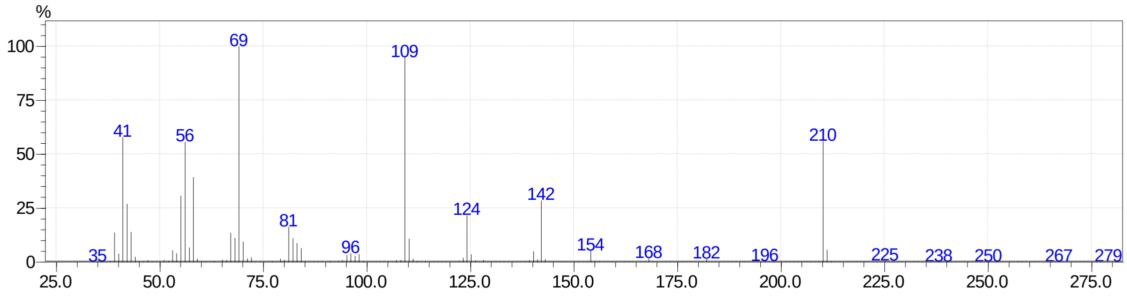

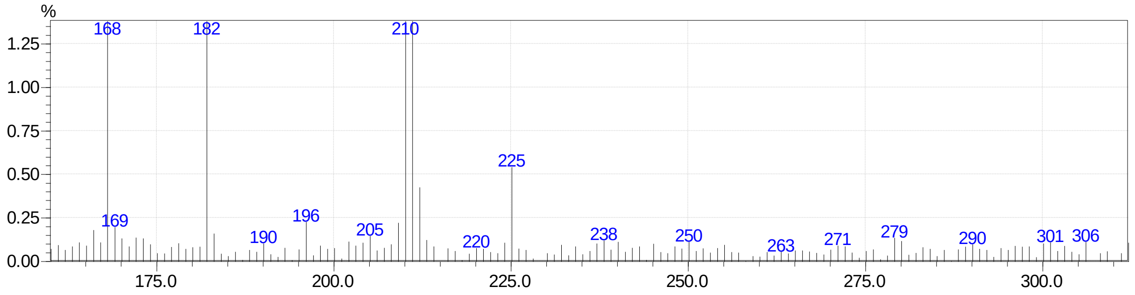

The product **TEMPO–C(O)CF$_3$** was further confirmed by GC–MS analysis. **GC–MS** (EI): *m/z* calcd for $C_{11}H_{18}F_3NO_2$ $[M]^+$ 253; found 253. The product peak was observed at 13.19 min.

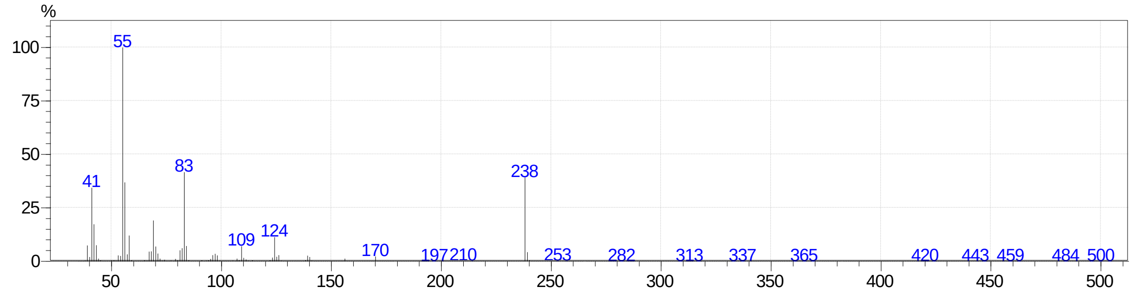

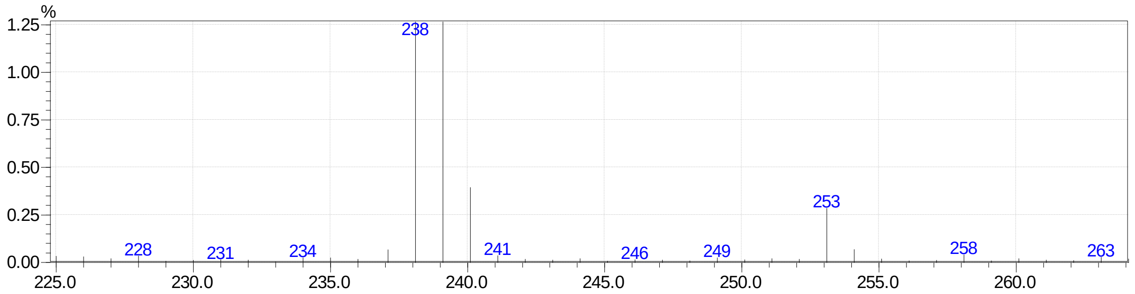

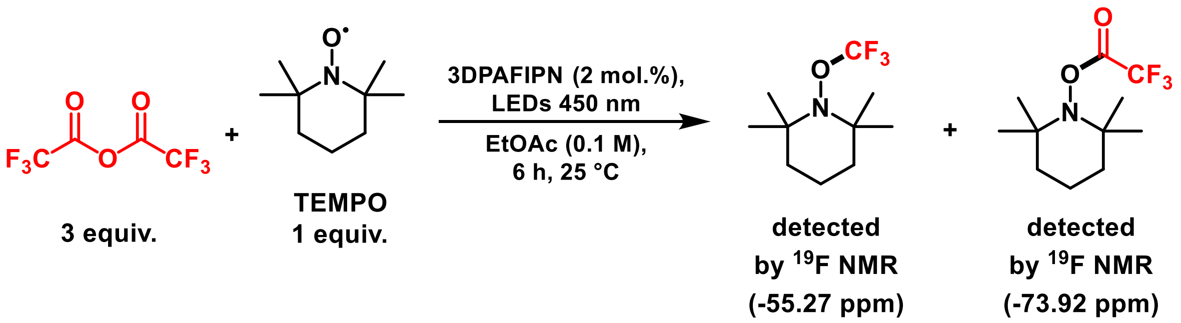

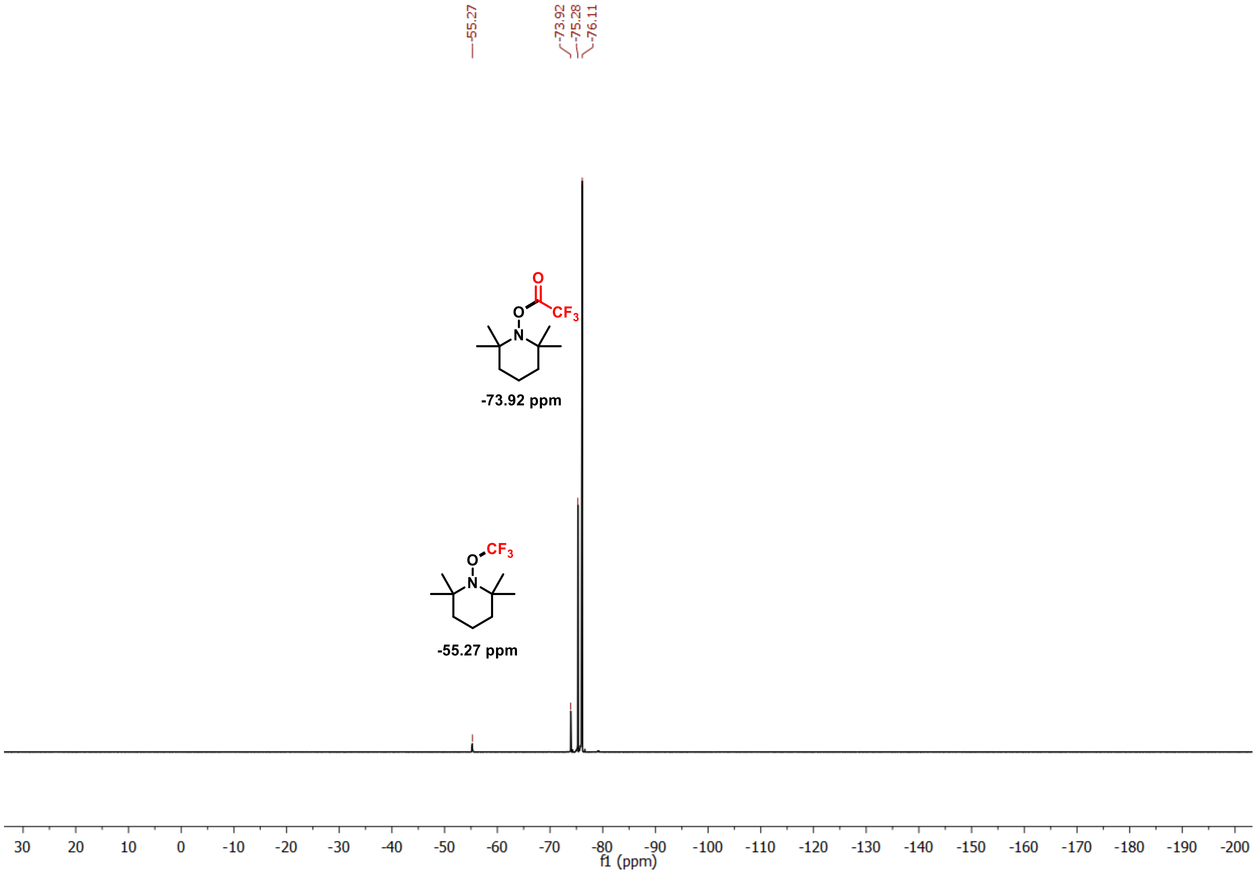

^{19}F NMR spectra of radical trapping with TFAA and TEMPO

5. Scaling-up

For scaling-up the photochemical reaction, two methods were used: increasing the number of identical tubes or using a reaction vessel with a larger diameter and size according to the General procedure.

1) Trifluoromethylation of 403 mg of 1,3,5-trimethoxybenzene in 8 vials

Each of 8 Pyrex tube (7.5 mL, No. 99447, i.d. = 11 mm) equipped with a magnetic stirring bar were charged with 3DPAFIPN (0.02 equiv, 0.006 mmol, 3.9 mg) and 1,3,5-trimethoxybenzene (50.4 mg, 1 equiv, 0.3 mmol). Then the atmosphere inside the vials were changed into argon. Dry ethyl acetate (3 mL) was added using syringe under argon atmosphere via PTFE/silicone septa and then TFAA (3 equiv, 0.9 mmol, 125 µL) was added. A stream of argon was bubbled through the reaction mixture for 1 minute in an ultrasonic bath. Eight reaction vials were placed in the photoreactor zone (blue LEDs, λ = 450 nm) and irradiated for 6 h. After the reaction, the mixture was combined, and 160 µL (190.4 mg, 1.304 mmol) of benzotrifluoride ($C_6H_5CF_3$) was added as standard and the mixture stirred for 1 minute. For quantitative ^{19}F NMR 0.3 mL of the resulting solution was transferred to NMR tube and diluted with 0.3 mL of deuterated solvent (DMSO-d_6 or $CDCl_3$). ^{19}F NMR yield was 67%.

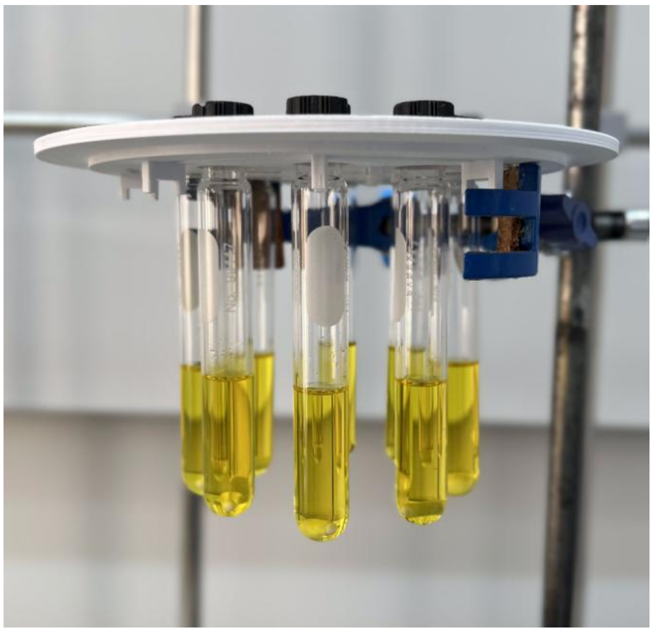

Figure S9. The reaction mixture in 8 pcs of 7.5 mL Pyrex tube (i.d. = 11 mm)

2) Trifluoromethylation of 403 mg of 1,3,5-trimethoxybenzene in Schlenk flask

A 50 mL Schlenk flask (borosilicate, i.d. = 30 mm) equipped with a magnetic stirring bar was charged with 3DPAFIPN (0.02 equiv, 0.048 mmol, 31.1 mg) and 1,3,5-trimethoxybenzene (403 mg, 1 equiv, 2.4 mmol). Then the atmosphere inside the vials were changed into argon via

vacuum pump and argon balloon. Dry ethyl acetate (24 mL) was added using syringe under argon atmosphere via rubber septa and then TFAA (3 equiv, 7.2 mmol, 1.0 mL) was added. A stream of argon was bubbled through the reaction mixture for 1 minute in an ultrasonic bath. Then the flask was placed in the photoreactor zone (blue LEDs, λ = 450 nm) and irradiated for 6 h. After the reaction, 160 μL (190.4 mg, 1.304 mmol) of benzotrifluoride ($C_6H_5CF_3$) as standard was added and the mixture stirred for 1 minute. For quantitative ^{19}F NMR 0.3 mL of the resulting solution was transferred to NMR tube and diluted with 0.3 mL of deuterated solvent (DMSO-d_6 or $CDCl_3$). ^{19}F NMR yield was 51%.

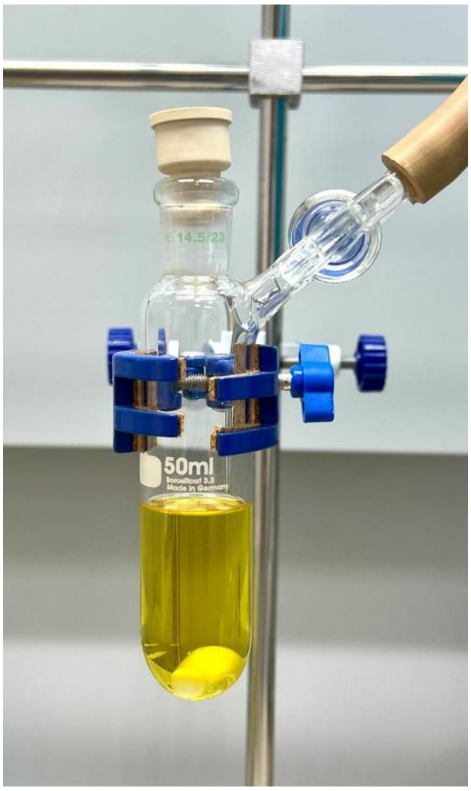

Figure S10. The reaction mixture (24 mL) in a 50 mL flask (i.d. = 30 mm)

The reaction of 1,3,5-trimethoxybenzene (403 mg, 2.4 mmol, 1 equiv) afforded product **1** in 51% yield (^{19}F NMR). Purification by silica gel column chromatography (cyclohexane/EtOAc, 100/0 to 95/5) gave product **1** (220 mg, 39%).

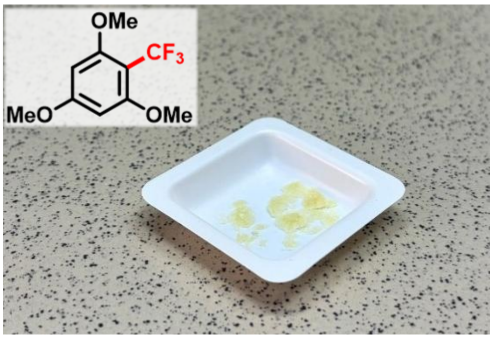

Figure S11. Crystals of 1,3,5-trimethoxy-2-(trifluoromethyl)benzene (**1**)

^{1}H NMR (400 MHz, $CDCl_3$) of 1,3,5-trimethoxy-2-(trifluoromethyl)benzene (**1**)

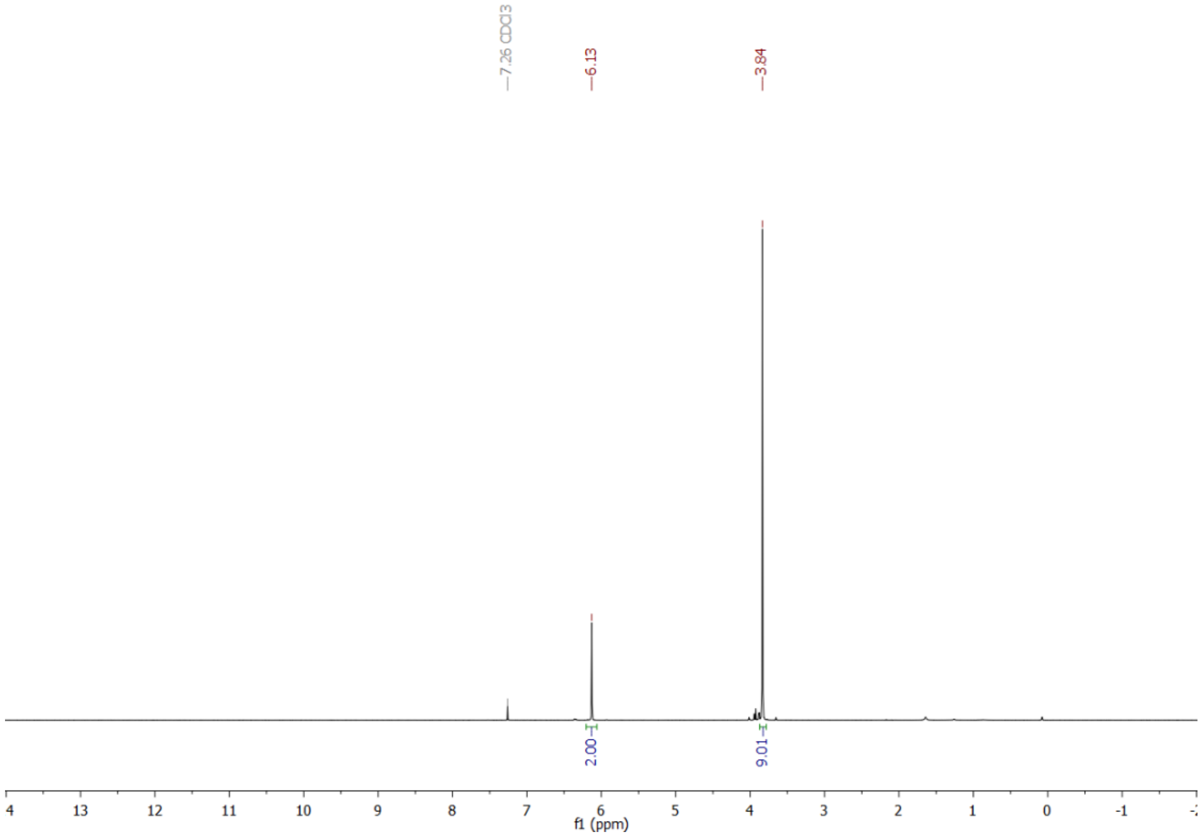

^{19}F NMR (376 MHz, $CDCl_3$) of 1,3,5-trimethoxy-2-(trifluoromethyl)benzene (**1**)

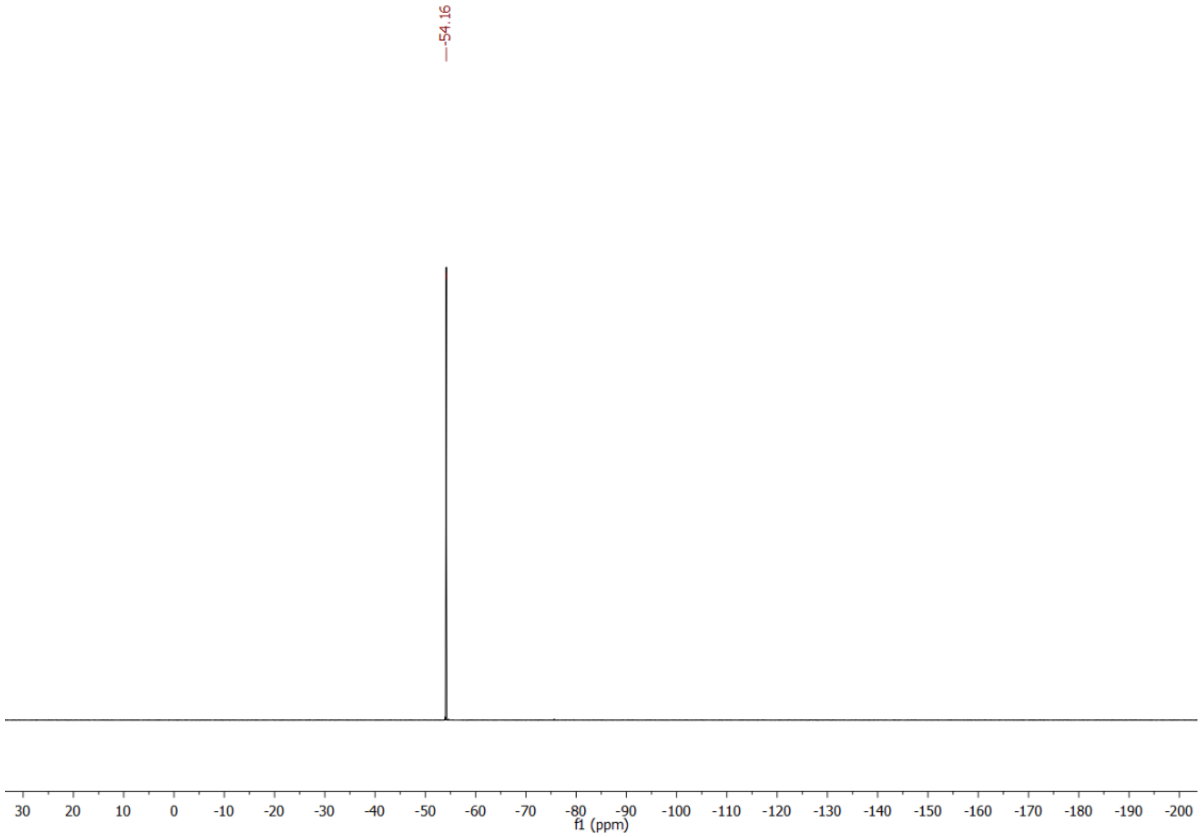

Table S8. Trifluoromethylation of 403 mg of 1,3,5-trimethoxybenzene

Vessels	^{19}F NMR yield, %
8 vials	67
Schlenk flask	51

Conditions: 403 mg (2.4 mmol) of 1,3,5-trimethoxybenzene, EtOAc (0.1 M), 2 mol % 3DPAFIPN, blue LEDs, 6 h, 25 °C

3) Trifluoromethylation of 1000 mg of 1,3,5-trimethoxybenzene in Schlenk flask

A 100 mL Schlenk flask (borosilicate, i.d. = 38 mm) equipped with a magnetic stirring bar were charged with 3DPAFIPN (0.02 equiv, 0.12 mmol, 77.8 mg) and 1,3,5-trimethoxybenzene (1000 mg, 1 equiv, 6.0 mmol). Then the atmosphere inside the vials was changed into argon via vacuum pump and argon balloon. Dry ethyl acetate (60 mL) was added using syringe under argon atmosphere via rubber septa and then TFAA (3 equiv, 18.0 mmol, 2.5 mL) was added. A stream of argon was bubbled through the reaction mixture for 1 minute in an ultrasonic bath. Then the flask was placed in the photoreactor zone (blue LEDs, λ = 450 nm) and irradiated for 6 h. After the reaction, 400 µL (476 mg, 3.26 mmol) of benzotrifluoride ($C_6H_5CF_3$) was added as standard and the mixture stirred for 1 minute. For quantitative ^{19}F NMR 0.3 mL of the resulting solution was transferred to NMR tube and diluted with 0.3 mL of deuterated solvent (DMSO-d_6 or $CDCl_3$). ^{19}F NMR yield was 51%.

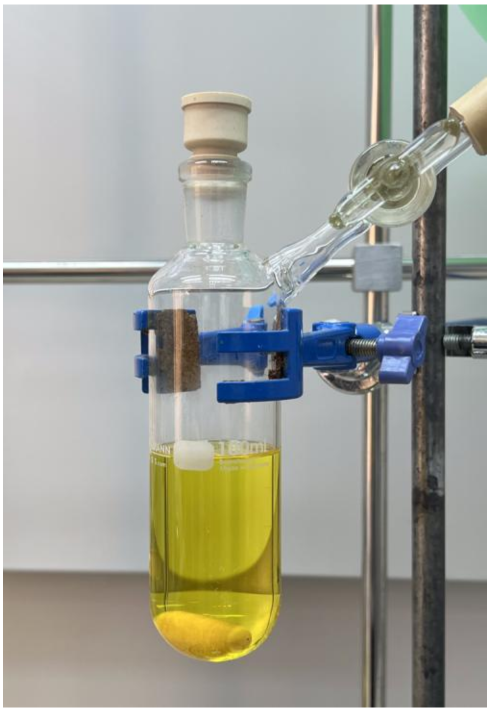

Figure S12. The reaction mixture (60 mL) in a 100 mL flask (i.d. = 38 mm)

Table S9. Trifluoromethylation of 1,3,5-trimethoxybenzene in flask

Scale	^{19}F NMR yield, %
Schlenk flask (403 mg, 24 mL, I.D. = 30 mm)	51
Schlenk flask (1000 mg, 60 mL, I.D. = 38 mm)	51

Conditions: EtOAc (0.1 M), 2 mol.% 3DPAFIPN, blue LEDs, 6 h, 25 °C

4) *Trifluoromethylation of 50.4 mg of 1,3,5-trimethoxybenzene in flow (i.d. = 0.75 mm) with 3 mol % of 3DPAFIPN*

A 10 mL Schlenk flask equipped with a magnetic stirring bar was charged with 3DPAFIPN (0.03 equiv, 0.009 mmol, 5.9 mg) and 1,3,5-trimethoxybenzene (50.4 mg, 1 equiv, 0.3 mmol). Then the atmosphere inside the vial was changed into argon via vacuum pump and argon balloon. Dry ethyl acetate (3 mL) was added using syringe under argon atmosphere via rubber septa and then TFAA (3 equiv, 0.9 mmol, 125 µL) was added. A stream of argon was bubbled through the reaction mixture for 1 minute in an ultrasonic bath. Then the solution was loaded into a 5 mL plastic syringe (B.Braun). The syringe was then fitted to a syringe pump and connected to a 3 mL FEP microreactor coil (internal diameter of 750 µm, Figure S4) via a Luer adapter, previously flushed with dry ethyl acetate. The flow rate (mL/min) was set to obtain a residence time of 30 minutes. After the photoirradiation zone, the reaction mixture was collected in a flask. When the syringe was fully empty, again dry ethyl acetate was loaded into a syringe and injected to collect all in a round-bottomed flask. Then 20 µL (23.8 mg, 0.163 mmol) of benzotrifluoride ($C_6H_5CF_3$) as standard was added as standard to the mixture and the mixture stirred for 1 minute. For quantitative ^{19}F NMR 0.3 mL of the resulting solution was transferred to NMR tube and diluted with 0.3 mL of deuterated solvent (DMSO-d_6 or $CDCl_3$). ^{19}F NMR yield was 71 %.

Green metrics

The adopted green metrics have been calculated employing the following equations [1-4]:

$$\textbf{STY}\ \text{(Space Time Yield)} = \frac{\text{mass of product}}{\text{reactor volume} \times \text{reaction time}}$$

$$\textbf{Productivity} = \frac{\text{mass of product}}{\text{reaction time}}$$

Entry	Amount of TMB	Reaction vessel	Time	^{19}F NMR yield, %	Productivity, mg/h	STY, g L^{-1} h^{-1}
1	8 vials × 0.3 mmol (2.4 mmol, 403 mg)	8 vials (i.d. = 11 mm)	6 h	67	63	1.05
2	2.4 mmol (403 mg)	Schlenk flask (i.d. = 30mm)	6 h	51	48	0.96
3	6.0 mmol (1000 mg)	Schlenk flask (i.d. = 38 mm)	6 h	51	120	1.20
4	50.4 mg (TMB is contained in 1 reactor volume)	Flow setup, *3 mol % PC* (i.d. = 0.75 mm, *V* = 3 mL)	0.5 h	71	101	33.51

6. Photophysical investigations

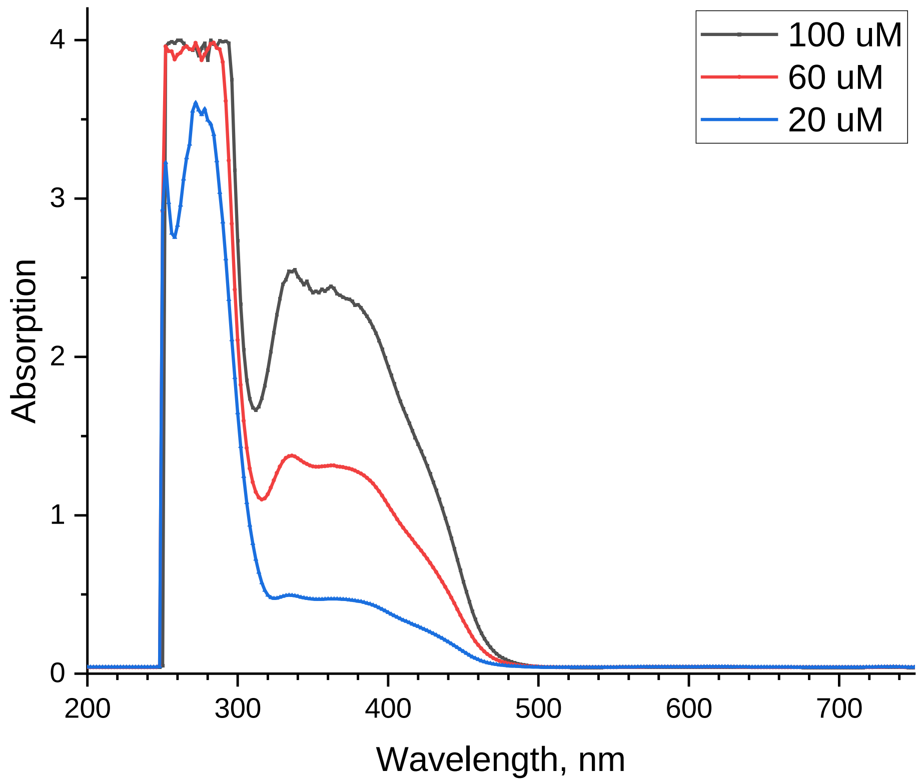

Figure S13. Absorption spectra of 3DPAFIPN at various concentrations in EtOAc

7. Stern–Volmer quenching studies

The quenching efficiency can be defined as Stern–Volmer relationship:

$$\frac{I_0}{I} - 1 = k_q\tau_0 \cdot [quencher]$$

Where I_0 being the luminescence intensity in the absence of any quencher, I is the luminescence intensity in the presence of a predefined quencher concentration while τ_0 being

the excited state lifetime of the photocatalyst which has been previously reported as τ_0 = 4.2 ns for 3DPAFIPN [5].

Preparation of stock solutions for Stern–Volmer measurements:

3DPAFIPN: a stock solution of photocatalyst (0.02 mM) was prepared by dissolving 0.156 mg (0.24 μmol) in 12 mL EtOAc in Schlenk flask under argon atmosphere.

Trifluoroacetic anhydride (TFAA): a stock solution of TFAA (40 mM) was prepared by dissolving 66.8 μL (100.8 mg, 0.48 mmol) in 12 mL EtOAc in Schlenk flask under argon atmosphere.

1,3,5-Trimethoxybenzene (TMB): A stock solution of TMB (40 mM) was prepared by dissolving 80.8 mg (0.48 mmol) in 12 mL EtOAc in Schlenk flask under argon atmosphere.

Quartz cuvettes (3.5 mL, 10 mm path length, silica cap) were filled with the photocatalyst stock solution (3 mL), the relevant amount of the reagent stock solution (or pure reagent) and EtOAc to obtain a total volume of 3.5 mL. All the prepared solutions were degassed for 5 minutes with a stream of argon. The solution was excited at a wavelength of 450 nm and the emission spectra was recorded from 456 to 700 nm.

1) *TFAA*. First the influence on the emission spectrum of the catalyst by TFAA was investigated.

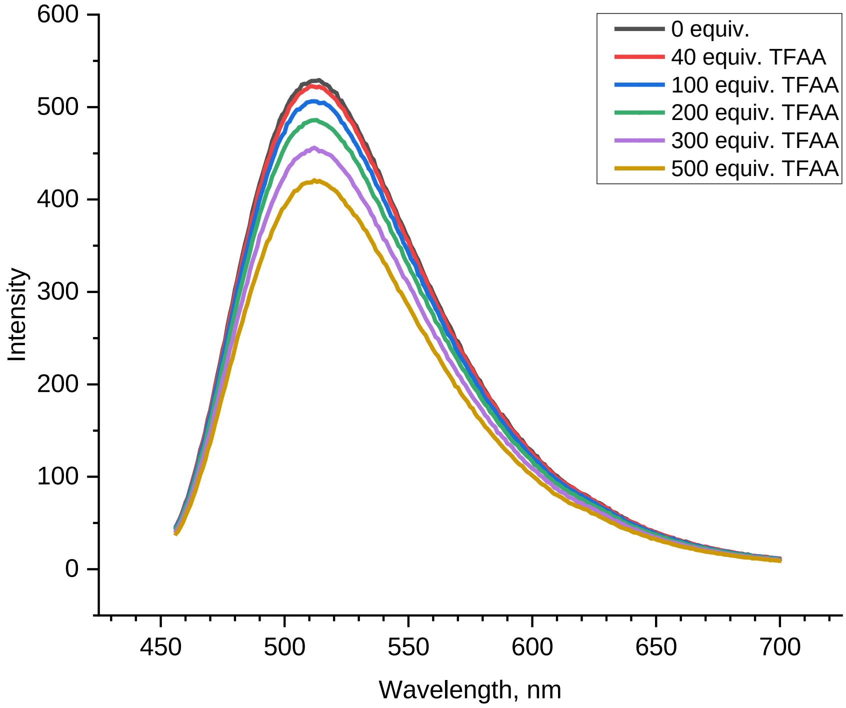

Figure S14. Emission quenching of 3DPAFIPN with TFAA (EtOAc, 0.02 mM 3DPAFIPN, excitation wavelength = 450 nm, λ_{em} = 456-700 nm, $\lambda_{max.em}$ = 512 nm)

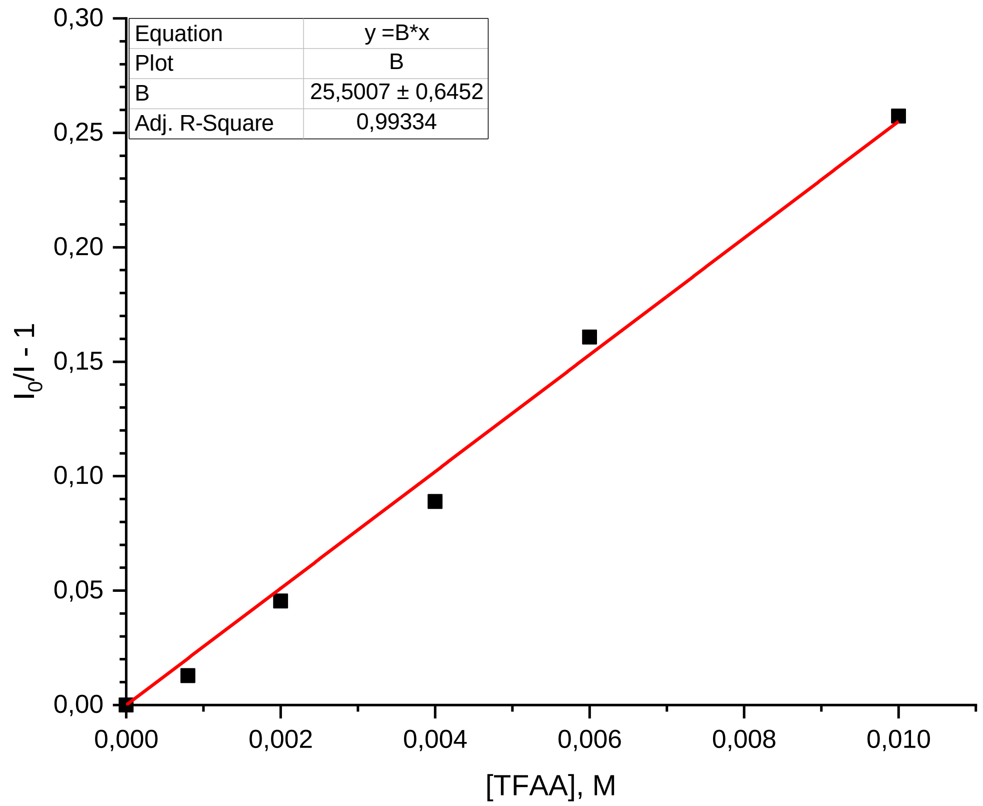

Figure S15. Stern–Volmer plot of the above data ($y = 25.5007 \cdot x$). The quenching constant K_q = 6.07·10^9 M^{-1}·s^{-1} was determined using the Stern–Volmer equation and a lifetime of as τ_0 = 4.2 ns for 3DPAFIPN

2) *1,3,5-Trimethoxybenzene (TMB)*. First the influence on the emission spectrum of the catalyst by TFAA was investigated.

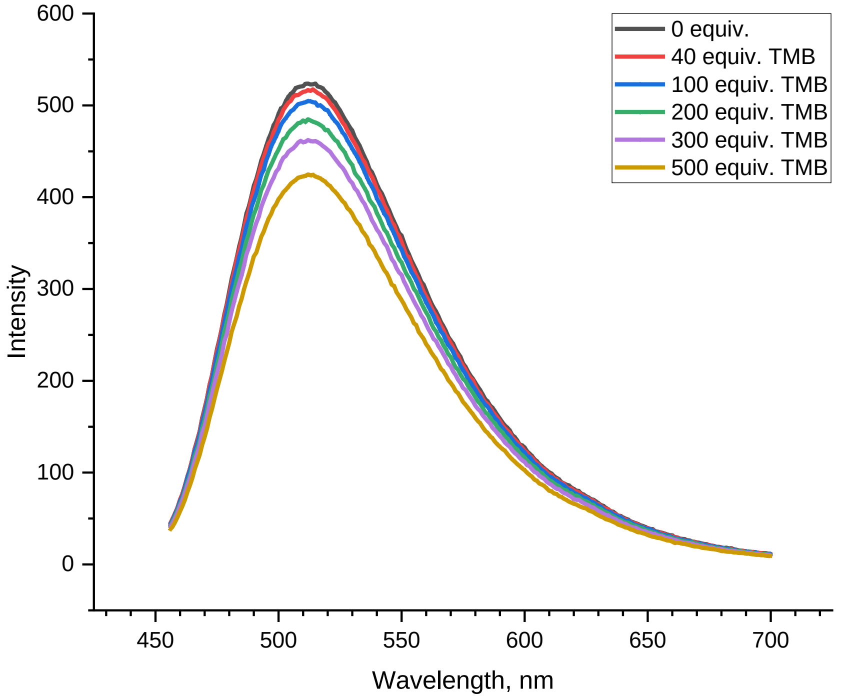

Figure S16. Emission quenching of 3DPAFIPN with 1,3,5-trimethoxybenzene (EtOAc, 0.02 mM 3DPAFIPN, excitation wavelength = 450 nm, λ_{em} = 456-700 nm, $\lambda_{max.em}$ = 512 nm)

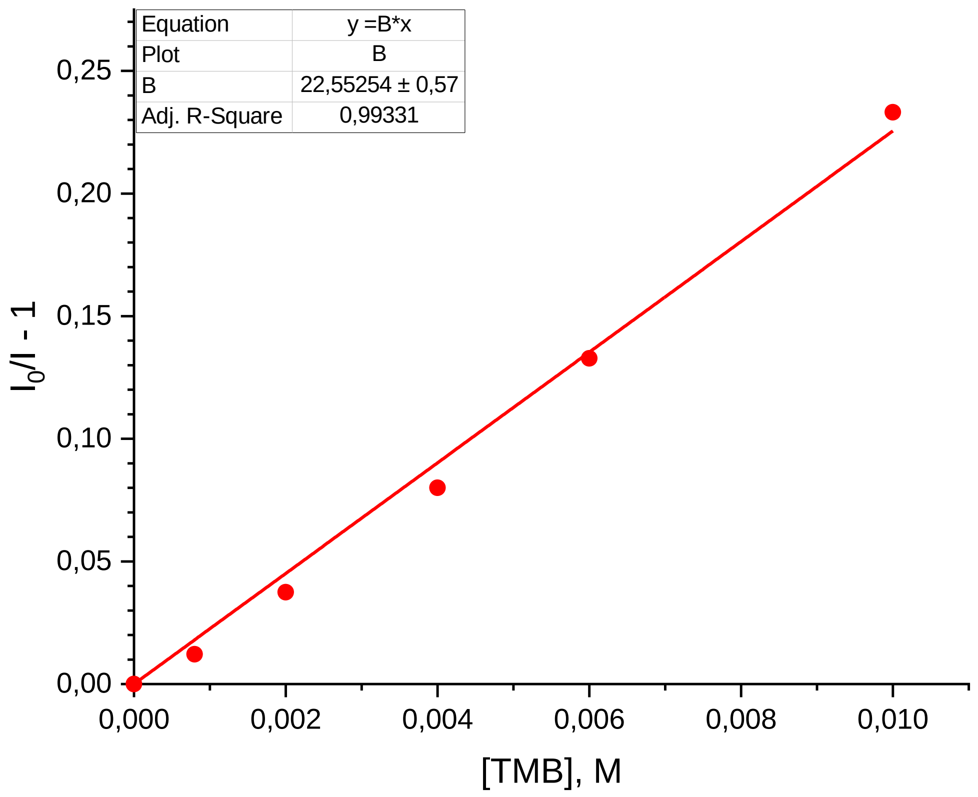

Figure S17. Stern–Volmer plot of the above data ($y = 22.55254 \cdot x$). The quenching constant K_q = 5.37·10^{9} M^{-1}·s^{-1} was determined using the Stern–Volmer equation and a lifetime of as τ_0 = 4.2 ns for 3DPAFIPN

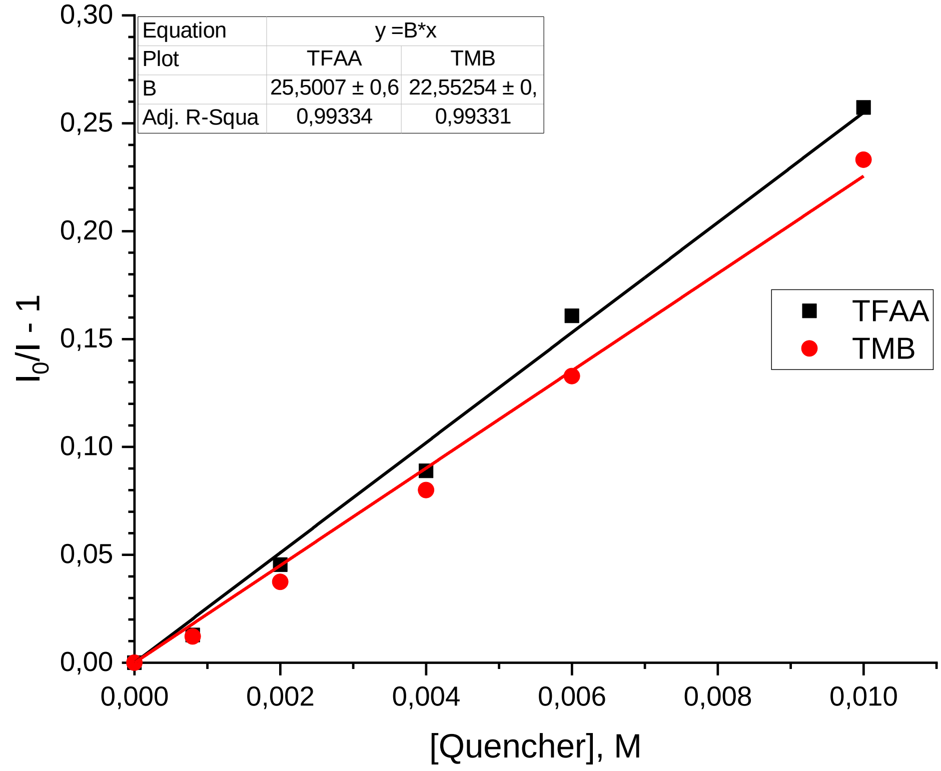

Figure S18. Stern–Volmer plots of both TFAA and TMB

8. Synthesis of starting materials

General procedure for methylation of phenols

In a round-bottomed flask the corresponding phenol (5 mmol, 1 equiv) was dissolved in acetone (50 mL). Then potassium carbonate (3.1 g, 22.5 mmol, 4.5 equiv) and dimethylsulfate (1.42 mL, 1.89 g, 15.0 mmol, 3.0 equiv) were added (* for one –OH group). The reaction mixture was refluxed for 18 h, progress of reaction was monitored by TLC. The mixture was quenched with ammonia (15% aq., 50 mL). The aqueous layer was extracted with ethyl acetate (3 × 50 mL). The organic layer was washed with brine (50 mL) and dried over sodium sulfate. The crude product was purified by silica gel column chromatography using EtOAc/CyH.

For known compounds, the spectra completely matched that reported in the literature [6–11].

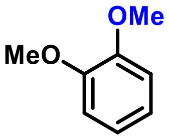

A1, 71 %
[6]

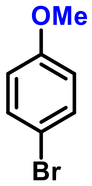

A2, 77 %
[7]

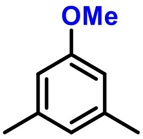

A3, 78 %
[8]

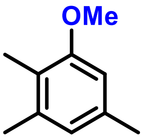

A4, 90 %
[9]

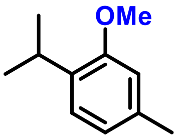

A5, 73 %
[10]

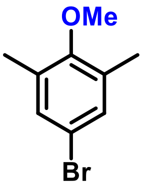

A6, 93 %
[7]

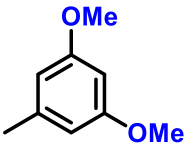

A8, 87 %
[11]

9. Scope and characterization of products

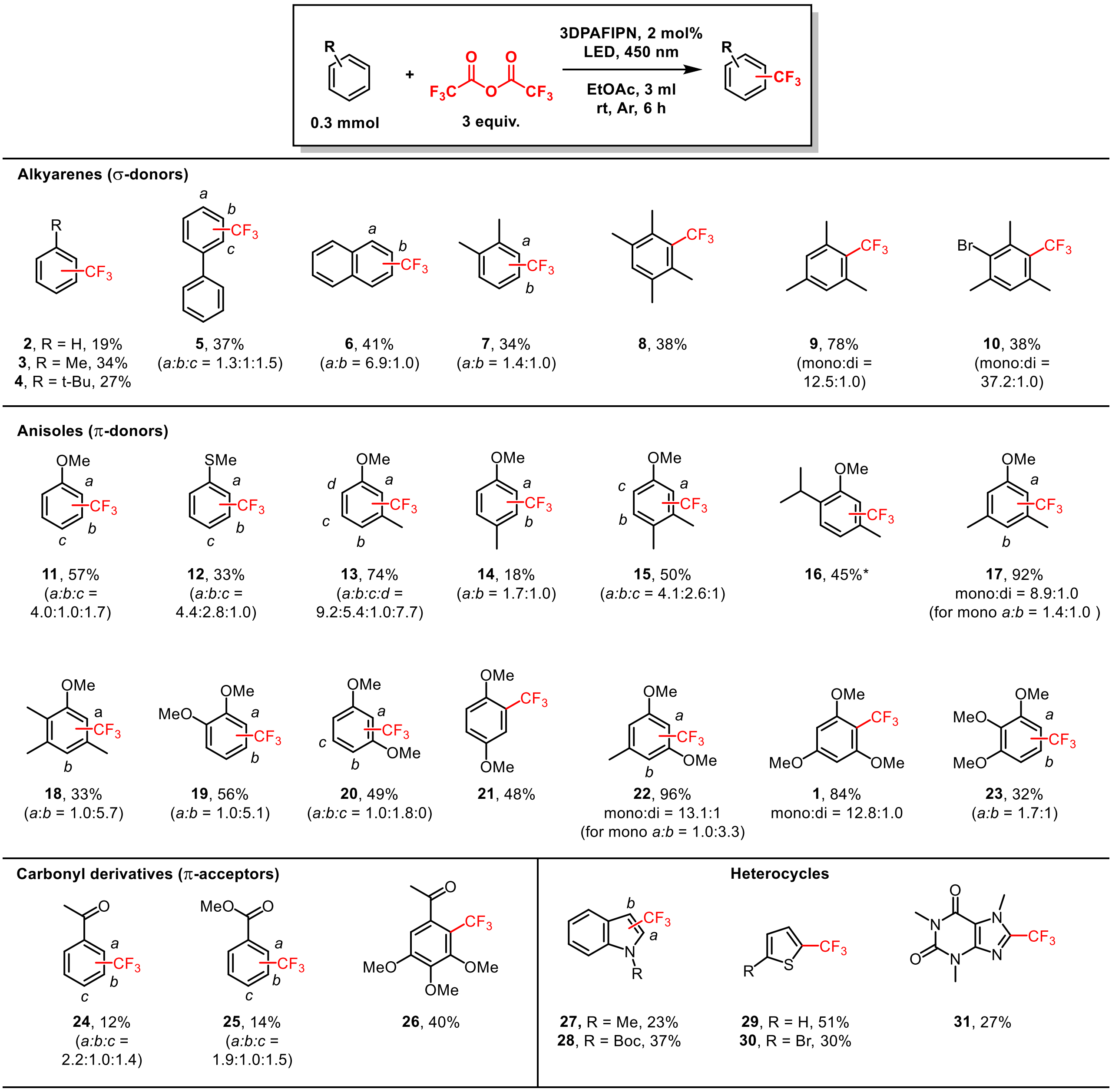

Scheme S2.

Haloarenes (σ-acceptors)

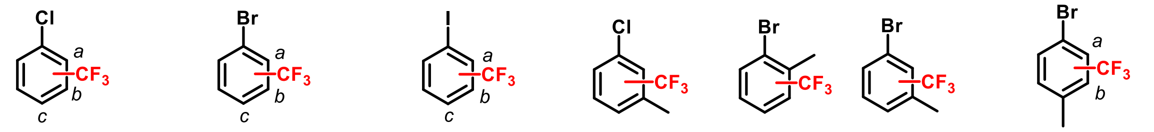

32, 17% (*a*:*b*:*c* = 1.3:2.6:1.0) | **33**, 13% (*a*:*b*:*c* = 2.4:1.3:1.0) | **34**, 16% (*a*:*b*:*c* = 2.0:1.0:1.1) | **35**, 20% | **36**, 10% | **37**, 13% | **38**, 16% (*a*:*b* = 1.0:1.4)

Brominated anisoles and methylanisoles

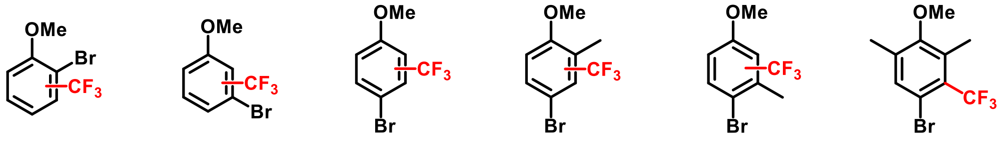

39, 26% | **40**, 30% | **41**, 22% | **42**, 12% | **43**, 42% | **44**, 22%

Scheme S3. Reaction with halobenzenes and halotoluenes

Unreactive substrates:

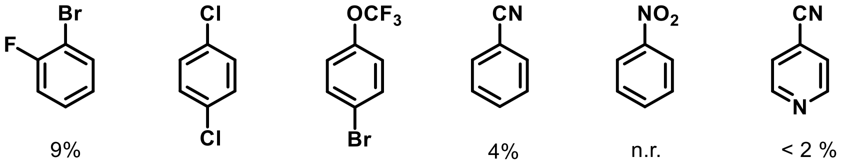

9% | 4% | < 2 % | 4% | n.r. | < 2 %

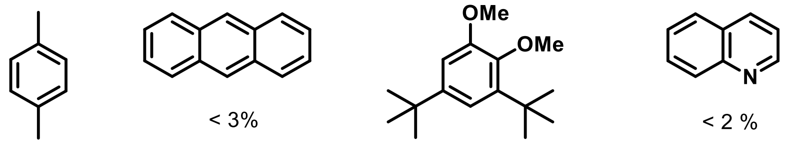

4% | < 3% | < 3 % | < 2 %

Scheme S4.

Don't react via the direct trifluoromethylation pathway (reactions proceed differently):

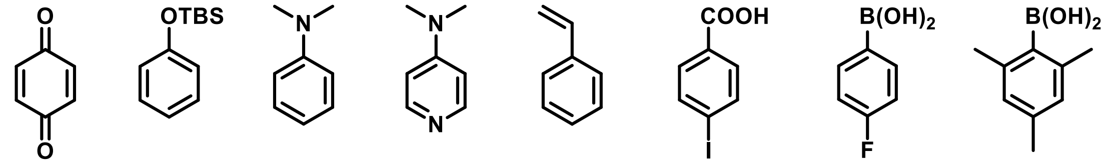

Scheme S5.

Contain a reactive fragment O–H, N–H which give esters and amides:

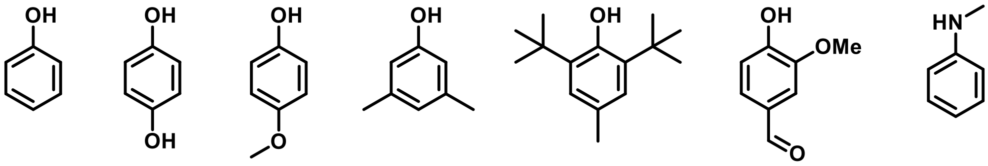

Scheme S6.

(1) Trifluoromethylation of 1,3,5-trimethoxybenzene

The reaction was carried out using 1,3,5-trimethoxybenzene (50.4 mg, 0.3 mmol) according to the General procedure.

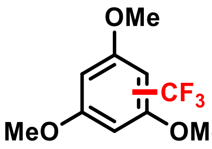

^{19}F NMR yield: 84%

(mono-CF_3:di-CF_3 = 12.8:1.0)

(1) 1,3,5-Trimethoxy-2-(trifluoromethyl)benzene

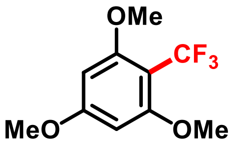

^{19}F NMR (376 MHz, $CDCl_3$/EtOAc): δ = −54.17 (s, 3F).

The data matched that of reported in the literature: δ = −54.14 ($CDCl_3$) [12].

(1a) 1,3,5-Trimethoxy-2,4-bis(trifluoromethyl)benzene

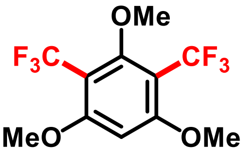

^{19}F NMR (376 MHz, $CDCl_3$/EtOAc): δ −55.51 (s, 6F).

The data matched that of reported in the literature: δ = −55.49 ($CDCl_3$) [12].

(2) Trifluoromethylation of benzene

The reaction was carried out using benzene (23.4 mg, 0.3 mmol) and 1-bromo-3-(trifluoromethyl)benzene ($BrC_6H_4CF_3$) as standard (20 µL, 32.4 mg, 0.144 mmol) according to the modified General procedure.

(2) (Trifluoromethyl)benzene (benzotrifluoride)

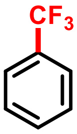

^{19}F NMR yield: 19 %

^{19}F NMR (376 MHz, DMSO-d_6/EtOAc): δ −62.20 (s, 3F).

The data matched that of reported in the literature: δ = −62.91 ($CDCl_3$) [13].

(3) Trifluoromethylation of toluene

The reaction was carried out using methylbenzene (27.6 mg, 0.3 mmol) according to the General procedure.

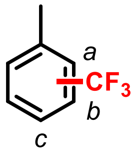

2 mol % 3DPAFIPN – **^{19}F NMR yield: 34%**

(*a*:*b*:*c* = 3.1:1.3:1.0)

(3a) 1-Methyl-2-(trifluoromethyl)benzene

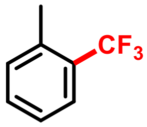

^{19}F NMR (376 MHz, DMSO-d_6/EtOAc): δ −61.22 (s, 3F).

The data matched that of reported in the literature: δ = −61.7 ($CDCl_3$) [14].

(3b) 1-Methyl-3-(trifluoromethyl)benzene

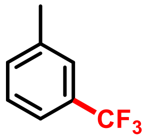

^{19}F NMR (376 MHz, DMSO-d_6/EtOAc): δ −62.12 (s, 3F).

The data matched that of reported in the literature: δ = −62.67 ($CDCl_3$) [14].

(3c) 1-Methyl-4-(trifluoromethyl)benzene

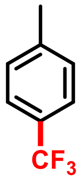

^{19}F NMR (376 MHz, DMSO-d_6/EtOAc): δ −61.77 (s, 3F).

The data matched that of reported in the literature: δ = −62.27 ($CDCl_3$) [14].

(4) Trifluoromethylation of *tert*-butylbenzene

The reaction was carried out using *tert*-butylbenzene (40.2 mg, 0.3 mmol) according to the General procedure.

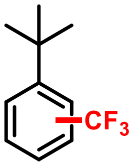

^{19}F NMR yield of mixture of isomers: 27%

(5) Trifluoromethylation of biphenyl

The reaction was carried out using biphenyl (46.2 mg, 0.3 mmol) according to the General procedure.

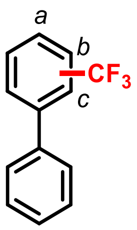

^{19}F NMR yield: 37%

(*a*:*b*:*c* = 1.3:1.0:1.5)

(5a) 4-(Trifluoromethyl)-1,1'-biphenyl

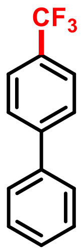

^{19}F NMR (376 MHz, DMSO-d_6/EtOAc): δ −61.92 (s, 3F).

The data matched that of reported in the literature: δ = −62.4 ($CDCl_3$) [15].

(5b) 3-(Trifluoromethyl)-1,1'-biphenyl

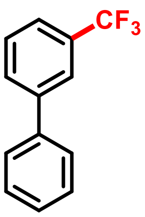

^{19}F NMR (376 MHz, DMSO-d_6/EtOAc): δ −62.08 (s, 3F).

The data matched that of reported in the literature: δ = −62.6 ($CDCl_3$) [16].

(5c) 2-(Trifluoromethyl)-1,1'-biphenyl

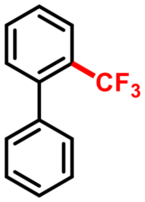

^{19}F NMR (376 MHz, DMSO-d_6/EtOAc): δ = −56.26 (s, 3F).

The data matched that of reported in the literature: δ = −56.8 ($CDCl_3$) [15].

(6) Trifluoromethylation of naphthalene

The reaction was carried out using naphthalene (38.4 mg, 0.3 mmol) according to the General procedure.

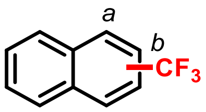

^{19}F NMR yield: 41%

(*a*:*b* = 6.8:1.0)

(6a) 1-(Trifluoromethyl)naphthalene

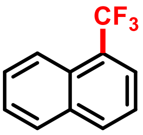

^{19}F NMR (376 MHz, DMSO-d_6/EtOAc) δ = −59.39 (d, *J* = 1.7 Hz, 3F).

The data matched that of reported in the literature: δ = −59.7 ($CDCl_3$) [16,17].

(6b) 2-(Trifluoromethyl)naphthalene

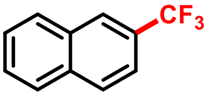

^{19}F NMR (376 MHz, DMSO-d_6/EtOAc) δ = −61.70 (s, 3F).

The data matched that of reported in the literature: δ = −62.8 ($CDCl_3$) [18].

(7) Trifluoromethylation of 1,2-dimethylbenzene (*o*-xylene)

The reaction was carried out using 1,2-dimethylbenzene (31.8 mg, 0.3 mmol) according to the General procedure.

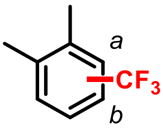

^{19}F NMR yield: 34%

(*a*:*b* = 1.0:1.4)

(7a) 1,2-Dimethyl-3-(trifluoromethyl)benzene

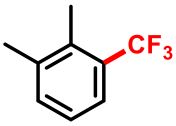

^{19}F NMR (376 MHz, DMSO-d_6/EtOAc): δ = −59.9 (s, 3F).

The data matched that of reported in the literature: δ = −60.4 ($CDCl_3$) [19].

(7b) 1,2-Dimethyl-4-(trifluoromethyl)benzene

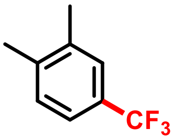

^{19}F NMR (376 MHz, DMSO-d_6/EtOAc): δ = −61.71 (s, 3F).

The data matched that of reported in the literature: δ = −62.3 ($CDCl_3$) [19].

(8) Trifluoromethylation of 1,2,4,5-Tetramethylbenzene (durene)

The reaction was carried out using 1,2,4,5-tetramethylbenzene (40.2 mg, 0.3 mmol) according to the General procedure.

1,2,4,5-Tetramethyl-3-(trifluoromethyl)benzene

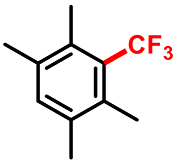

^{19}F NMR yield: 38%.

^{19}F NMR (376 MHz, DMSO-d_6/EtOAc): δ = −51.35 (hept, J = 3.2 Hz, 3F).

The data matched that of reported in the literature: δ = −51.74 ($CDCl_3$) [20].

(9) Trifluoromethylation of 1,3,5-trimethylbenzene (mesitylene)

The reaction was carried out using 1,3,5-trimethylbenzene (36 mg, 0.3 mmol) according to the General procedure.

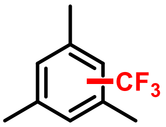

^{19}F NMR yield: 78%

(mono-CF_3:di-CF_3 = 12.5:1.0)

(9a) 1,3,5-Trimethyl-2-(trifluoromethyl)benzene

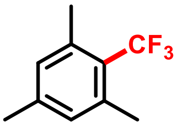

^{19}F NMR (376 MHz, $CDCl_3$/EtOAc): δ = −53.80 (p, *J* = 3.7 Hz, 3F).

The data matched that of reported in the literature: δ = −53.76 ($CDCl_3$) [21].

(9aa) 1,3,5-Trimethyl-2,4-bis(trifluoromethyl)benzene

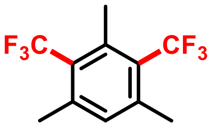

^{19}F NMR (376 MHz, $CDCl_3$/EtOAc): δ = −53.00 (p, *J* = 3.4 Hz, 3F).

The data matched that of reported in the literature: δ = −52.97 ($CDCl_3$) [21].

(10) Trifluoromethylation of 2-bromo-1,3,5-trimethylbenzene

The reaction was carried out using 2-bromo-1,3,5-trimethylbenzene (59.7 mg, 0.3 mmol) according to the General procedure.

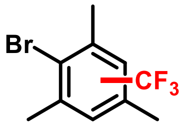

^{19}F NMR yield: 38%

(mono-CF_3:di-CF_3 = 37.2:1.0)

(10a) 2-Bromo-1,3,5-trimethyl-4-(trifluoromethyl)benzene

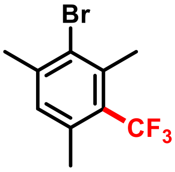

^{19}F NMR (376 MHz, $CDCl_3$/EtOAc): δ = −53.12 to −58.19 (m, 3F).

The data matched that of reported in the literature: δ = −54.10 ($CDCl_3$) [22].

(10aa) 1-Bromo-2,4,6-trimethyl-3,5-bis(trifluoromethyl)benzene

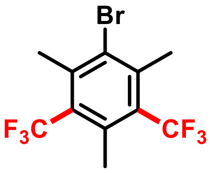

^{19}F NMR (376 MHz, $CDCl_3$/EtOAc): δ −52.35 to −52.42 (m, 6F).

(11) Trifluoromethylation of anisole

The reaction was carried out using methoxybenzene (32.4 mg, 0.3 mmol) according to the General procedure.

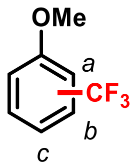

^{19}F NMR yield: 57%

(*a*:*b*:*c* = 4.0:1.0:1.7)

(11a) 1-Methoxy-2-(trifluoromethyl)benzene

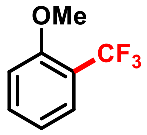

^{19}F NMR (376 MHz, $CDCl_3$/EtOAc): δ = −62.58 (s, 3F).

The data matched that of reported in the literature: δ = −62.4 ($CDCl_3$) [12].

(11b) 1-Methoxy-3-(trifluoromethyl)benzene

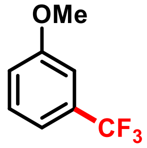

^{19}F NMR (376 MHz, $CDCl_3$/EtOAc): δ = −62.88 (s, 3F).

The data matched that of reported in the literature: δ = −62.8 ($CDCl_3$) [12].

(11c) 1-Methoxy-4-(trifluoromethyl)benzene

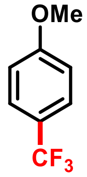

^{19}F NMR (376 MHz, $CDCl_3$/EtOAc): δ = −61.63 (s, 3F).

The data matched that of reported in the literature: δ = −61.5 ($CDCl_3$) [12].

(12) Trifluoromethylation of thioanisole

The reaction was carried out using thioanisole (36.9 mg, 0.3 mmol) according to the General procedure.

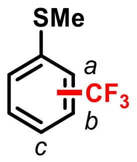

^{19}F NMR yield: 33%

(*a*:*b*:*c* = 4.4:2.8:1.0)

(12a) Methyl(2-(trifluoromethyl)phenyl)sulfane

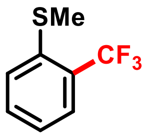

^{19}F NMR (376 MHz, $CDCl_3$/EtOAc): δ = −61.94 (s, 3F).

The data matched that of reported in the literature: δ = −61.5 ($CDCl_3$) [23].

The data matched that of reported in the literature: δ = −61.4 ($CDCl_3$) [12].

(12b) Methyl(3-(trifluoromethyl)phenyl)sulfane

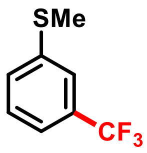

^{19}F NMR (376 MHz, $CDCl_3$/EtOAc): δ = −62.47 (s, 3F).

The data matched that of reported in the literature: δ = −61.8 ($CDCl_3$) [12].

(12c) Methyl(4-(trifluoromethyl)phenyl)sulfane

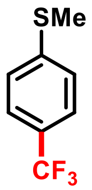

^{19}F NMR (376 MHz, $CDCl_3$/EtOAc): δ = −63.03 (s, 3F).

The data matched that of reported in the literature: δ = −62.3 ($CDCl_3$) [23].

The data matched that of reported in the literature: δ = −62.4 ($CDCl_3$) [12].

(13) Trifluoromethylation of 1-methoxy-3-methylbenzene (3-methylanisole)

The reaction was carried out using 1-methoxy-3-methylbenzene (36.6 mg, 0.3 mmol) according to the General procedure.

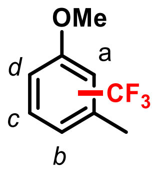

^{19}F NMR yield: 74%

(*a*:*b*:*c*:*d* = 9.2:5.4:1.0:7.7)

(13a) 1-Methoxy-3-methyl-2-(trifluoromethyl)benzene

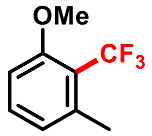

^{19}F NMR (376 MHz, DMSO-*d*$_6$/EtOAc): δ = −54.23 (q, J = 3.7 Hz, 3F).

The data matched that of reported in the literature: δ = −53.74 ($CDCl_3$) [24].

(13b) 4-Methoxy-2-methyl-1-(trifluoromethyl)benzene

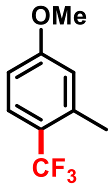

^{19}F NMR (376 MHz, DMSO-*d*$_6$/EtOAc): δ = −61.35 (s, 3F).

The data matched that of reported in the literature: δ = −60.72 ($CDCl_3$) [24].

(13c) 1-Methoxy-3-methyl-5-(trifluoromethyl)benzene

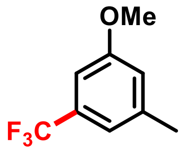

^{19}F NMR (376 MHz, DMSO-*d*$_6$/EtOAc): δ = −62.14 (s, 3F).

The data matched that of reported in the literature: δ = −62.7 ($CDCl_3$) [25].

(13d) 2-Methoxy-4-methyl-1-(trifluoromethyl)benzene

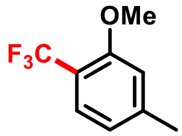

^{19}F NMR (376 MHz, DMSO-*d*$_6$/EtOAc): δ = −59.70 (s, 3F).

The data matched that of reported in the literature: δ = −59.11 ($CDCl_3$) [24].

(14) Trifluoromethylation of 1-methoxy-4-methylbenzene (4-methylanisole)

The reaction was carried out using 4-methylanisole (36.6 mg, 0.3 mmol) according to the General procedure.

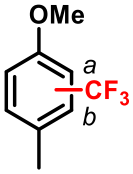

^{19}F NMR yield: 18%

(*a*:*b* = 1.7:1.0)

(14a) 1-Methoxy-4-methyl-2-(trifluoromethyl)benzene

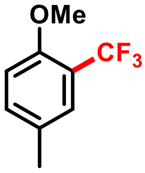

^{19}F NMR (376 MHz, DMSO-d_6/EtOAc): δ = –61.69 (s, 3F)

The data matched that of reported in the literature: δ = –62.2 ($CDCl_3$) [12].

(14b) 4-Methoxy-1-methyl-2-(trifluoromethyl)benzene

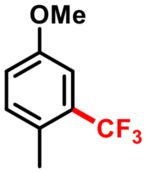

^{19}F NMR (376 MHz, DMSO-d_6/EtOAc): δ = –61.47 (d, 3F)

The data matched that of reported in the literature: δ = –61.3 ($CDCl_3$) [12].

(15) Trifluoromethylation of 4-methoxy-1,2-dimethylbenzene (3,4-dimethylanisole)

The reaction was carried out using 3,4-dimethylanisole (40.8 mg, 0.3 mmol) according to the General procedure.

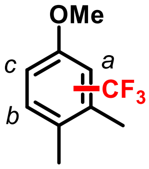

^{19}F NMR yield: 50%

(*a*:*b*:*c* = 4.1:1.0:2.6)

Isomers were identified based on the effect of through-space H–F coupling [26,27]:

(15a) 1-Methoxy-3,4-dimethyl-2-(trifluoromethyl)benzene

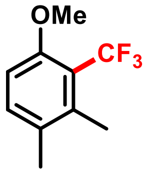

^{19}F NMR (376 MHz, DMSO-d_6/EtOAc): δ = –52.95 (q, J = 3.0 Hz, 3F).

The data matched that of reported in the literature: δ = –53.5 ($CDCl_3$) [28].

(15b) 5-Methoxy-1,2-dimethyl-3-(trifluoromethyl)benzene

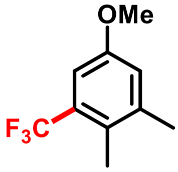

^{19}F NMR (376 MHz, DMSO-d_6/EtOAc): δ = –60.17 (d, J = 1.4 Hz, 3F).

The isomer was identified by exclusion.

(15c) 1-Methoxy-4,5-dimethyl-2-(trifluoromethyl)benzene

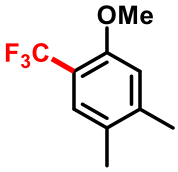

^{19}F NMR (376 MHz, DMSO-d_6/EtOAc): δ = –61.10 (s, 3F).

The data matched that of reported in the literature: δ = –61.7 ($CDCl_3$) [28].

(16) Trifluoromethylation of 1-isopropyl-2-methoxy-4-methylbenzene

The reaction was carried out using 1-isopropyl-2-methoxy-4-methylbenzene (49.2 mg, 0.3 mmol) according to the General procedure.

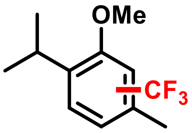

^{19}F NMR yield of mixture of isomers: 45%

Mixture (16): GC–MS (EI): m/z calcd for $C_{12}H_{15}F_3O$ $[M]^+$ 232; found 232. The products peaks were observed at 17.07 and 18.10 min.

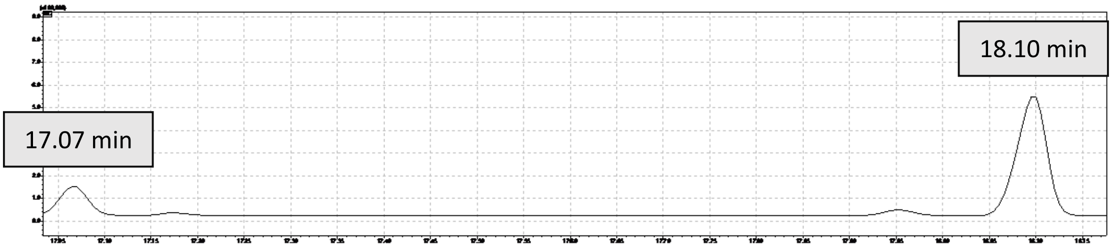

The EI–MS fragmentation spectra of compound, observed at 17.07 min:

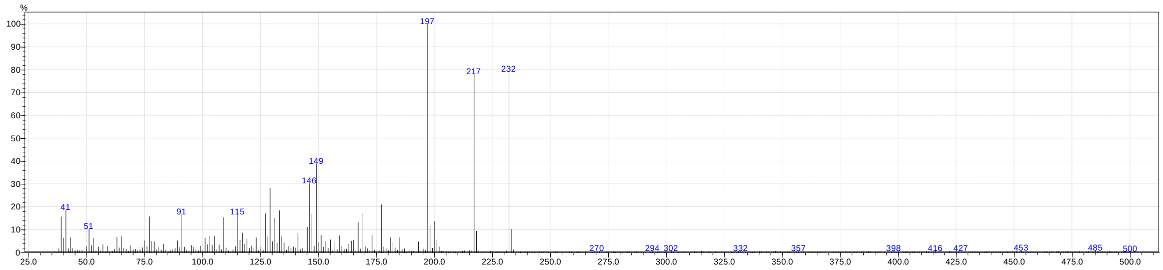

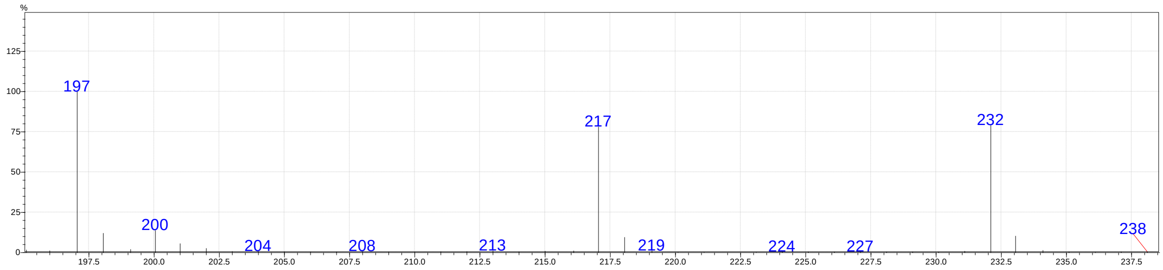

The EI–MS fragmentation spectra of compound, observed at 18.10 min:

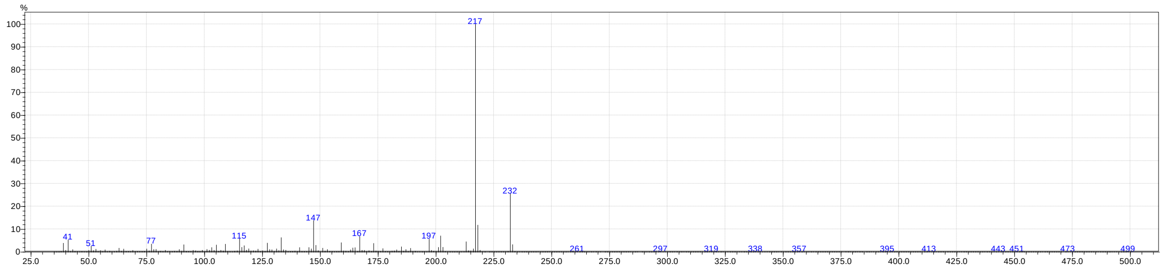

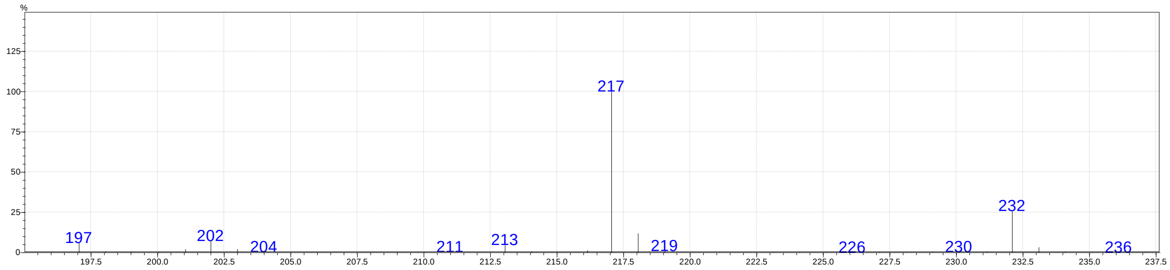

(17) Trifluoromethylation of 1-methoxy-3,5-dimethylbenzene

The reaction was carried out using 1-methoxy-3,5-dimethylbenzene (40.8 mg, 0.3 mmol) according to the General procedure.

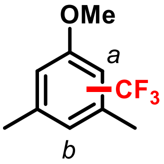

^{19}F NMR yield: 92%

(a:b = 1.4:1.0;

mono-CF_3:di-CF_3 = 8.9:1.0)

(17a) 1-Methoxy-3,5-dimethyl-2-(trifluoromethyl)benzene

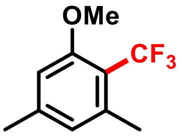

^{19}F NMR (376 MHz, $CDCl_3$/EtOAc): δ = –54.60 (q, J = 3.6 Hz, 3F).

The data matched that of reported in the literature: δ = –54.9 ($CDCl_3$) [29].

(17b) 5-Methoxy-1,3-dimethyl-2-(trifluoromethyl)benzene

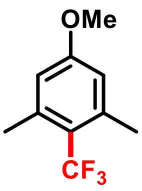

^{19}F NMR (376 MHz, $CDCl_3$/EtOAc): δ = –52.99 (hept, J = 3.4 Hz, 3F).

The data matched that of reported in the literature: δ = –53.3 ($CDCl_3$) [29].

(17ab) 1-Methoxy-3,5-dimethyl-2,4-bis(trifluoromethyl)benzene

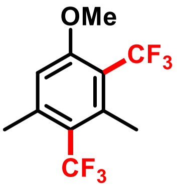

^{19}F NMR (376 MHz, $CDCl_3$/EtOAc): δ = –51.87 to –51.92 (m, 3F), –53.45 (q, 3F).

GC-MS (EI): m/z calcd for $C_{11}H_{10}F_6O$ $[M]^+$ 272; found 272. The products peak was observed at 16.71 min.

The EI–MS fragmentation spectra of compound (**17ab**):

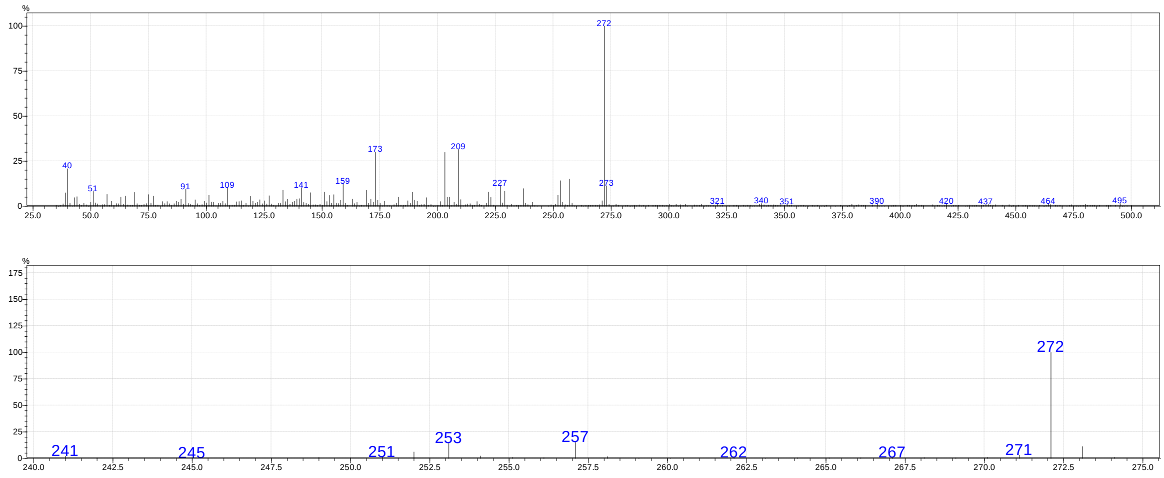

(18) Trifluoromethylation of 1-methoxy-2,3,5-trimethylbenzene

The reaction was carried out using 1-methoxy-2,3,5-trimethylbenzene (45.0 mg, 0.3 mmol) according to the General procedure.

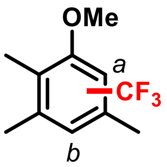

^{19}F NMR yield: 33%

(a:b = 1.0:5.7)

Isomers were identified based on the effect of through-space H–F coupling [26,27]:

(18a) 3-Methoxy-1,2,5-trimethyl-4-(trifluoromethyl)benzene

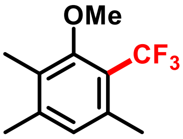

^{19}F NMR (376 MHz, DMSO-d_6/EtOAc): δ = –54.13 (q, J = 3.3 Hz, 3F).

GC–MS (EI): m/z calcd for $C_{11}H_{13}F_3O$ $[M]^+$ 218; found 218. The products peak was observed at 17.45 min.

The EI–MS fragmentation spectra of compound (**18a**):

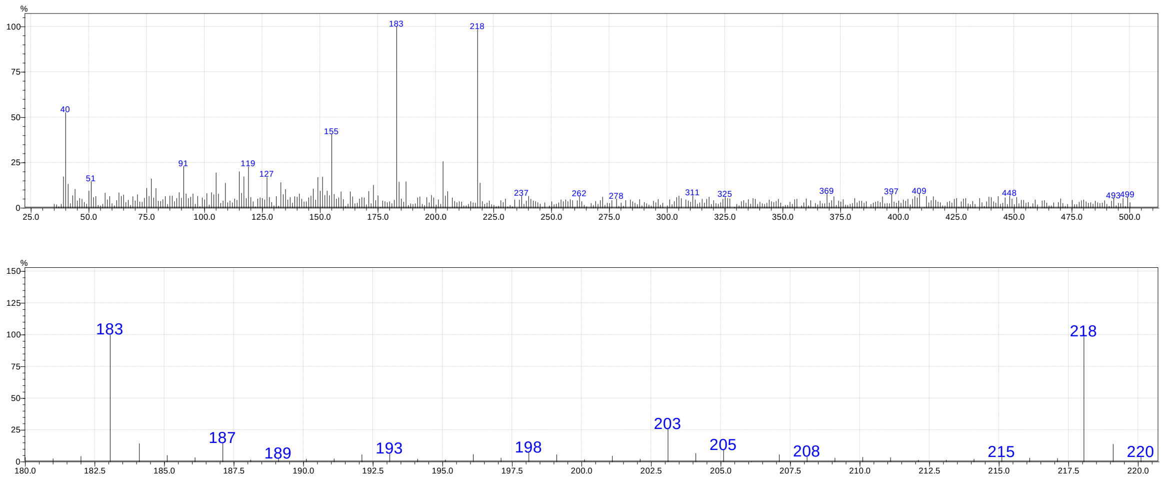

(18b) 1-Methoxy-2,3,5-trimethyl-4-(trifluoromethyl)benzene

The product (**18b**) was purified by column chromatography (eluent: 100% CyH) or HPLC and characterized (MeCN/H_2O)**:**

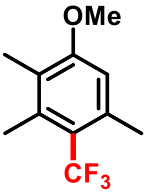

^{1}H NMR (400 MHz, $CDCl_3$): δ 6.54 (s, 1H), 3.83 (s, 1H), 2.47 (q, J = 4.1 Hz, 3H), 2.35 (q, J = 2.5 Hz, 3H), 2.14 (s, 3H).

^{19}F NMR (376 MHz, $CDCl_3$): δ = –51.60 to –51.67 (m).

^{13}C NMR (101 MHz, $CDCl_3$): δ 158.74 – 158.71 (m), 137.87 (q, J = 1.9 Hz), 135.71 (q, J = 2.1 Hz), 126.62 (q, J = 275.2 Hz), 124.44, 120.30 (q, J = 27.9 Hz), 111.52, 55.57, 22.59 (q, *J* = 4.8 Hz), 17.03 (q, J = 4.2 Hz), 11.75.

^{19}F NMR (376 MHz, DMSO-d_6/EtOAc): δ -51.02 (qq, *J* = 4.2, 2.3 Hz, 3F).

GC–MS (EI): m/z calcd for $C_{11}H_{13}F_3O$ [M+] 218; found 218. The products peak was observed at 19.02 min.

The EI–MS fragmentation spectra of compound (**18b**):

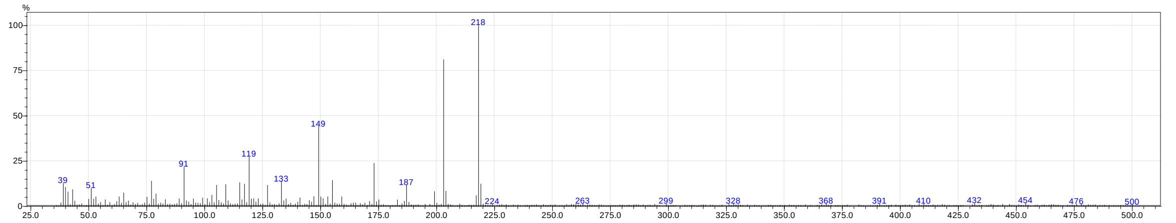

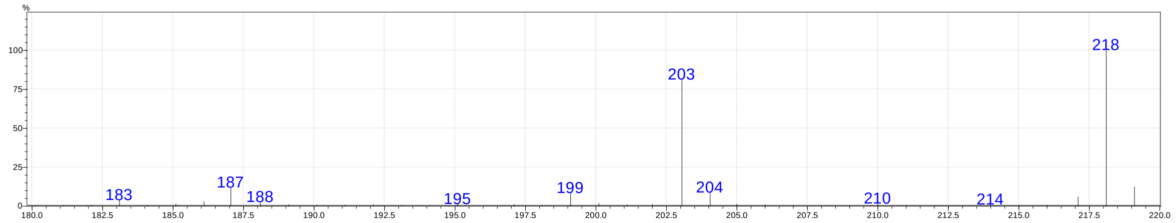

(19) Trifluoromethylation of 1,2-dimethoxybenzene

The reaction was carried out using 1,2 -dimethoxybenzene (41.4 mg, 0.3 mmol) according to the General procedure.

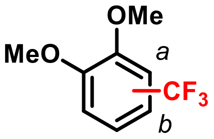

^{19}F NMR yield: 56%

(*a*:*b* = 1.0:5.1)

(19a) 1,2-Dimethoxy-3-(trifluoromethyl)benzene

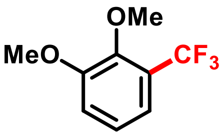

^{19}F NMR (376 MHz, DMSO-d_6/EtOAc): δ = −60.78 (s, 3F).

The data matched that of reported in the literature: δ = −61.36 ($CDCl_3$) [19] or δ = −61.3 ($CDCl_3$) [30].

(19b) 1,2-Dimethoxy-4-(trifluoromethyl)benzene

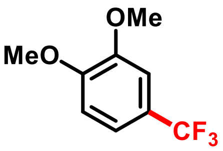

^{19}F NMR (376 MHz, DMSO-d_6/EtOAc): δ −60.82 (s, 3F).

The data matched that of reported in the literature: δ = −61.4 ($CDCl_3$) [31] or δ = −61.6 ($CDCl_3$) [32].

(20) Trifluoromethylation of 1,3-dimethoxybenzene

The reaction was carried out using 1,3-dimethoxybenzene (41.4 mg, 0.3 mmol) according to the General procedure.

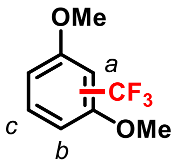

^{19}F NMR yield: 49%

(a:b:c = 1.0:1.8:0)

(20a) 1,3-Dimethoxy-2-(trifluoromethyl)benzene

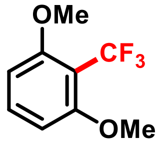

^{19}F NMR (376 MHz, DMSO-d_6/EtOAc): δ = –54.11 (s, 3F).

The data matched that of reported in the literature: δ = –54.9 ($CDCl_3$) [31].

(20b) 2,4-Dimethoxy-1-(trifluoromethyl)benzene

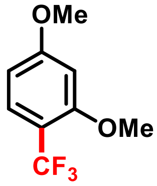

^{19}F NMR (376 MHz, DMSO-d_6/EtOAc): δ = –60.54 (s, 3F).

The data matched that of reported in the literature: δ = –61.3 ($CDCl_3$) [31].

(21) Trifluoromethylation of 1,4-dimethoxybenzene

The reaction was carried out using 1,4 -dimethoxybenzene (41.4 mg, 0.3 mmol) according to the General procedure.

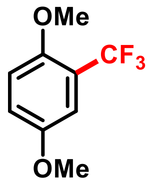

1,4-Dimethoxy-2-(trifluoromethyl)benzene

^{19}F NMR yield: 48%

^{19}F NMR (376 MHz, DMSO-d_6/EtOAc): δ = –61.79 (s, 3F).

The data matched that of reported in the literature: δ = –62.42 ($CDCl_3$) [12].

(22) Trifluoromethylation of 3,5-dimethoxytoluene

The reaction was carried out using 3,5-dimethoxytoluene (45.6 mg, 0.3 mmol) according to the General procedure.

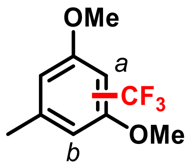

^{19}F NMR yield: 96%

(mono:bis = 13.1:1.0,

a:*b*:*ab*:*bb* = 8.8:29.4:1.0:1.9)

(22a) 1,3-Dimethoxy-5-methyl-2-(trifluoromethyl)benzene

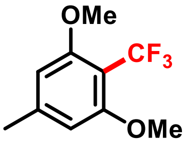

^{19}F NMR (376 MHz, DMSO-d_6/EtOAc): δ = –53.77 (s, 3F).

The data matched that of reported in the literature: δ = –54.63 ($CDCl_3$) [33].

(22b) 1,5-Dimethoxy-3-methyl-2-(trifluoromethyl)benzene

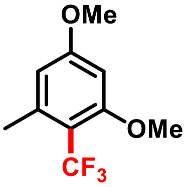

^{19}F NMR (376 MHz, DMSO-d_6/EtOAc): δ = –53.18 (q, J = 3.6 Hz, 3F).

The data matched that of reported in the literature: δ = –53.86 ($CDCl_3$) [33].

(22ab) 1,3-Dimethoxy-5-methyl-2,4-bis(trifluoromethyl)benzene

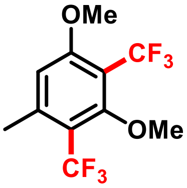

^{19}F NMR (376 MHz, DMSO-d_6/EtOAc): δ = –53.86 (q, *J* = 3.4 Hz, 3F), -56.04 (s, 3F).

GC–MS (EI): *m*/*z* calcd for $C_{11}H_{10}F_6O_2$ $[M]^+$ 288; found 288. The product peak was observed at 18.99 min.

The EI–MS fragmentation spectra of compound (**22ab**):

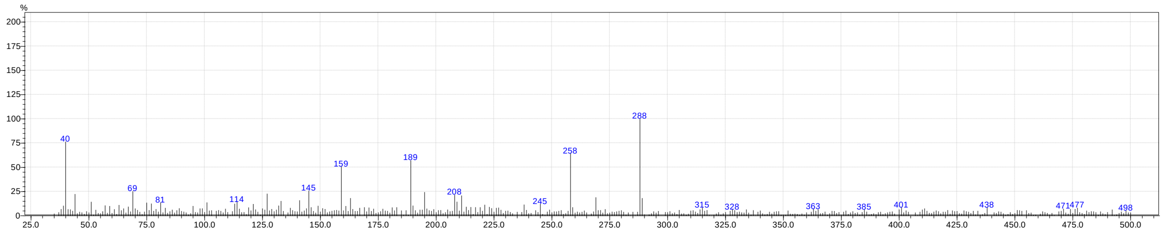

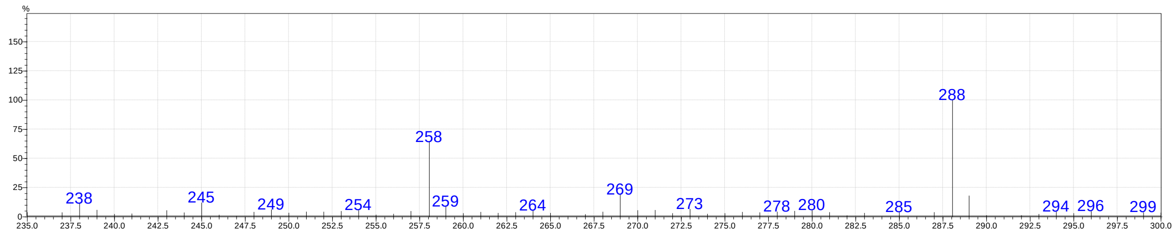

(22bb) 1,5-Dimethoxy-3-methyl-2,4-bis(trifluoromethyl)benzene

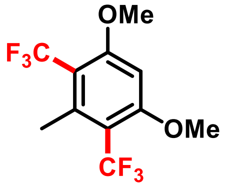

^{19}F NMR (376 MHz, DMSO-d_6/EtOAc): δ = –51.89 (q, J = 2.8 Hz, 6F).

GC–MS (EI): m/z calcd for $C_{11}H_{10}F_6O_2$ $[M]^+$ 288; found 288. The product peak was observed at 21.05 min.

The EI–MS fragmentation spectra of compound (**22bb**):

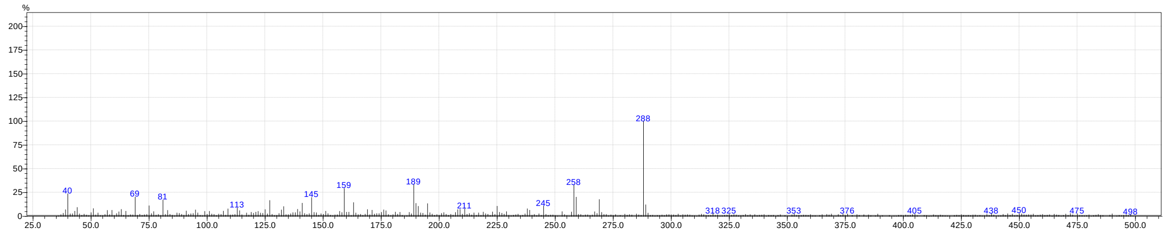

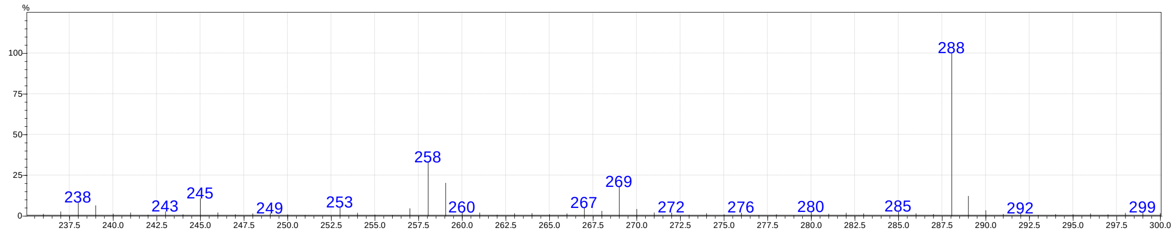

(23) Trifluoromethylation of 1,2,3-trimethoxybenzene

The reaction was carried out using 1,2,3-dimethoxybenzene (50.4 mg, 0.3 mmol) according to the General procedure.

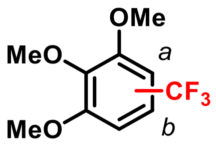

^{19}F NMR yield: 32%

(a:b = 1.7:1.0)

(23a) 1,2,3-Trimethoxy-4-(trifluoromethyl)benzene

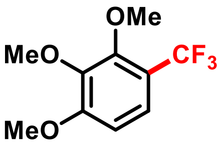

^{19}F NMR (376 MHz, DMSO-d_6/EtOAc): δ = –60.09 (s, 3F).

The data matched that of reported in the literature: δ = –60.86 ($CDCl_3$)[26].

(23b) 1,2,3-Trimethoxy-5-(trifluoromethyl)benzene

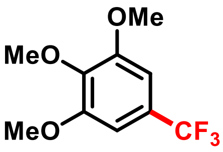

^{19}F NMR (376 MHz, DMSO-d_6/EtOAc): δ = –61.37 (s, 3F).

The data matched that of reported in the literature: δ = –62.07 ($CDCl_3$)[26].

(24) Trifluoromethylation of acetophenone

The reaction was carried out using acetophenone (36 mg, 0.3 mmol) according to the General procedure.

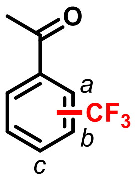

^{19}F NMR yield: 12%

(a:b:c = 2.2:1.0:1.4)

(24a) 1-(2-(Trifluoromethyl)phenyl)ethan-1-one

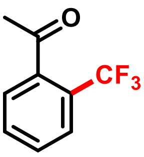

^{19}F NMR (376 MHz, DMSO-d_6/EtOAc) δ = –57.83 (s, 3F).

The data matched that of reported in the literature: δ = –58.2 ($CDCl_3$)[6].

(24b) 1-(3-(Trifluoromethyl)phenyl)ethan-1-one

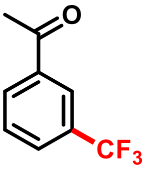

^{19}F NMR (376 MHz, DMSO-d_6/EtOAc) δ = –62.25 (s, 3F).

The data matched that of reported in the literature: δ = –62.8 ($CDCl_3$)[27].

(24c) 1-(4-(Trifluoromethyl)phenyl)ethan-1-one

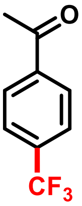

^{19}F NMR (376 MHz, DMSO-d_6/EtOAc) δ = –62.56 (s, 3F).

The data matched that of reported in the literature: δ = –63.1 ($CDCl_3$)[28].

(25) Trifluoromethylation of methyl benzoate

The reaction was carried out using methyl benzoate (40.8 mg, 0.3 mmol) according to the General procedure.

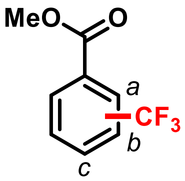

^{19}F NMR yield: 14%

(*a*:*b*:*c* = 1.9:1.0:1.5)

(25a) Methyl 2-(trifluoromethyl)benzoate

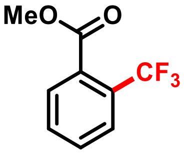

^{19}F NMR (376 MHz, $CDCl_3$/EtOAc): δ = –59.82 (s, 3F).

The data matched that of reported in the literature: δ = –59.79 ($CDCl_3$) [14].

(25b) Methyl 3-(trifluoromethyl)benzoate

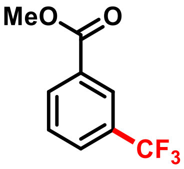

^{19}F NMR (376 MHz, $CDCl_3$/EtOAc): δ = –62.99 (s, 3F).

The data matched that of reported in the literature: δ = –62.82 ($CDCl_3$) [37].

(25c) Methyl 4-(trifluoromethyl)benzoate

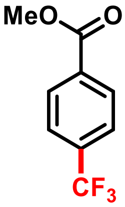

^{19}F NMR (376 MHz, $CDCl_3$/EtOAc): δ = –63.26 (s, 3F).

The data matched that of reported in the literature: δ = –63.27 ($CDCl_3$) [14].

(26) Trifluoromethylation of 1-(3,4,5-trimethoxyphenyl)ethan-1-one (3,4,5-trimethoxyacetophenone)

The reaction was carried out using 3,4,5-trimethoxyacetophenone (63 mg, 0.3 mmol) according to the General procedure.

(26) 1-(3,4,5-Trimethoxy-2-(trifluoromethyl)phenyl)ethan-1-one

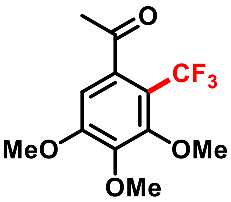

^{19}F NMR yield: 40%

^{19}F NMR (376 MHz, DMSO-d_6/EtOAc) δ = –54.72 (s, 3F).

The data matched that of reported in the literature: δ = –55.0 ($CDCl_3$) [31].

(27) Trifluoromethylation of 1-methyl-1H-indole

The reaction was carried out using 1-methyl-1H-indole (41.2 mg, 0.3 mmol) according to the General procedure.

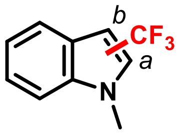

^{19}F NMR yield: 23%

(*a*:*b* = 1.0:1.1)

Isomer was identified based on the effect of through-space H–F coupling.

(27a) 1-Methyl-2-(trifluoromethyl)-1*H*-indole

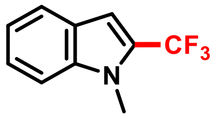

^{19}F NMR (376 MHz, DMSO-d_6/EtOAc): δ = –56.70 (qq, *J* = 5.67, 1.29 Hz, 3F).

The data matched that of reported in the literature: δ = –56.74 ($CDCl_3$) [38].

(27b) 1-Methyl-3-(trifluoromethyl)-1*H*-indole

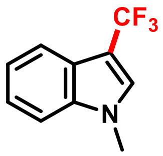

^{19}F NMR (376 MHz, DMSO-d_6/EtOAc): δ = –57.79 (s, 3F).

The data matched that of reported in the literature: δ = –56.76 ($CDCl_3$) [39].

(28) Trifluoromethylation of *tert*-butyl-1*H*-indole-1-carboxylate

The reaction was carried out using *tert*-butyl 1*H*-indole-1-carboxylate (65.1 mg, 0.3 mmol) according to the General procedure.

(28) *tert*-Butyl 3-(trifluoromethyl)-1*H*-indole-1-carboxylate

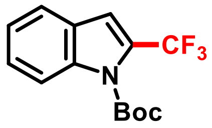

^{19}F NMR yield: 37%

^{19}F NMR (376 MHz, $CDCl_3$/EtOAc) δ = –58.32 (s, 3F).

The data matched that of reported in the literature: δ = –58.15 ($CDCl_3$) [40].

(29) Trifluoromethylation of thiophene

The reaction was carried out using thiophene (24.9 mg, 0.3 mmol) according to the General procedure.

(29) 2-(Trifluoromethyl)thiophene

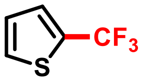

^{19}F NMR yield: 51%

^{19}F NMR (376 MHz, DMSO-d_6/EtOAc) δ = –54.43 to –54.46 (m, 3F).

The data matched that of reported in the literature: δ = –55.44 ($CDCl_3$) [41].

(30) Trifluoromethylation of 2-bromothiophene

The reaction was carried out using 2-bromothiophene (48.9 mg, 0.3 mmol) according to the General procedure.

(30) 2-Bromo-5-(trifluoromethyl)thiophene

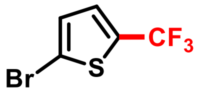

^{19}F NMR yield: 30%

^{19}F NMR (376 MHz, DMSO-d_6/EtOAc) δ = –55.34 to –55.39 (m, 3F).

The data matched that of reported in the literature: δ = –56.32 ($CDCl_3$) [42].

(31) Trifluoromethylation of caffeine

The reaction was carried out using caffeine (58.2 mg, 0.3 mmol) according to the General procedure.

(31) 1,3,7-Trimethyl-8-(trifluoromethyl)-3,7-dihydro-1*H*-purine-2,6-dione

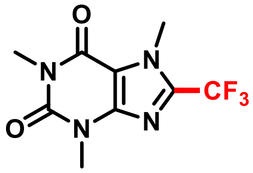

^{19}F NMR yield: 27%

^{19}F NMR (376 MHz, DMSO-d_6): δ = –62.16 (q, *J* = 1.4 Hz, 3F).

The data matched that of reported in the literature: δ = –62.7 ($CDCl_3$) [31].

(32) Trifluoromethylation of chlorobenzene

The reaction was carried out using chlorobenzene (33.8 mg, 0.3 mmol) according to the General procedure.

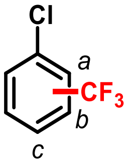

^{19}F NMR yield: 17%

(a:b:c = 1.3:2.6:1.0)

(32a) 1-Chloro-2-(trifluoromethyl)benzene

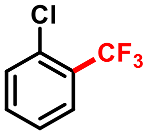

^{19}F NMR (376 MHz, DMSO-d_6/EtOAc): δ = –62.10 (s, 3F).

The data matched that of reported in the literature: δ = –62.65 ($CDCl_3$) [43].

(32b) 1-Chloro-3-(trifluoromethyl)benzene

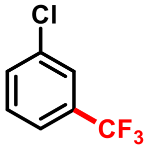

^{19}F NMR (376 MHz, DMSO-d_6/EtOAc): δ = –62.15 (s, 3F).

The data matched that of reported in the literature: δ = –62.67 ($CDCl_3$) [44].

(32c) 1-Chloro-4-(trifluoromethyl)benzene

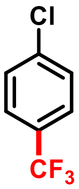

^{19}F NMR (376 MHz, DMSO-d_6/EtOAc): δ = –62.37 (s, 3F).

The data matched that of reported in the literature: δ = –62.92 ($CDCl_3$) [43].

(33) Trifluoromethylation of bromobenzene

The reaction was carried out using bromobenzene (47.1 mg, 0.3 mmol) according to the General procedure.

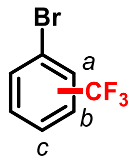

^{19}F NMR yield: 13%

(*a*:*b*:*c* = 2.4:1.3:1.0)

(33a) 1-Bromo-2-(trifluoromethyl)benzene

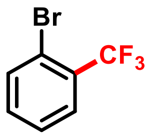

^{19}F NMR (376 MHz, DMSO-d_6/EtOAc): δ = −62.17 (s, 3F).

The data matched that of reported in the literature: δ = −62.70 ($CDCl_3$) [43].

(33b) 1-Bromo-3-(trifluoromethyl)benzene

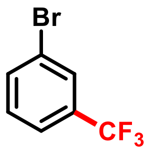

^{19}F NMR (376 MHz, DMSO-d_6/EtOAc): δ = −62.26 (s, 3F).

The data matched that of reported in the literature: δ = -62.79 ($CDCl_3$) [43].

(33c) 1-Bromo-4-(trifluoromethyl)benzene

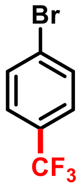

^{19}F NMR (376 MHz, DMSO-d_6/EtOAc): δ -62.36 (s, 3F).

The data matched that of reported in the literature: δ = −62.91 ($CDCl_3$) [43].

(34) Trifluoromethylation of iodobenzene

The reaction was carried out using iodobenzene (61.2 mg, 0.3 mmol) according to the General procedure.

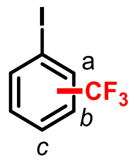

^{19}F NMR yield: 16%

(a:b:c = 2.0:1.0:1.1)

(34a) 1-Iodo-2-(trifluoromethyl)benzene

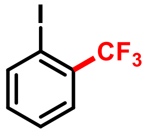

^{19}F NMR (376 MHz, DMSO-d_6/EtOAc): δ = –62.18 (s, 3F).

The data matched that of reported in the literature: δ = –62.81 ($CDCl_3$) [43].

(34b) 1-Iodo-3-(trifluoromethyl)benzene

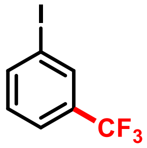

^{19}F NMR (376 MHz, DMSO-d_6/EtOAc): δ = –62.40 (s, 3F).

The data matched that of reported in the literature: δ = –62.90 ($CDCl_3$) [43].

(34c) 1-Iodo-4-(trifluoromethyl)benzene

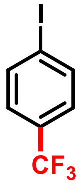

^{19}F NMR (376 MHz, DMSO-d_6/EtOAc): δ = –62.48 (s, 3F).

The data matched that of reported in the literature: δ = –62.99 ($CDCl_3$) [43].

(35) Trifluoromethylation of 1-chloro-3-methylbenzene (3-chlorotoluene)

The reaction was carried out using 1-chloro-3-methylbenzene (38 mg, 0.3 mmol) according to the General procedure.

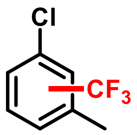

^{19}F NMR yield of mixture of isomers: 20%

(36) Trifluoromethylation of 1-bromo-2-methylbenzene (2-bromotoluene)

The reaction was carried out using 1-bromo-2-methylbenzene (51.3 mg, 0.3 mmol) according to the General procedure.

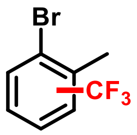

^{19}F NMR yield of mixture of isomers: 10%

(37) Trifluoromethylation of 1-bromo-3-methylbenzene (3-bromotoluene)

The reaction was carried out using 1-bromo-3-methylbenzene (51.3 mg, 0.3 mmol) according to the General procedure.

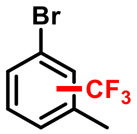

^{19}F NMR yield of mixture of isomers: 13%

(38) Trifluoromethylation of 1-bromo-4-methylbenzene (4-bromotoluene)

The reaction was carried out using 1-bromo-4-methylbenzene (51.3 mg, 0.3 mmol) according to the General procedure.

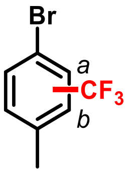

^{19}F NMR yield: 16%

(*a*:*b* = 1.0:1.4)

Isomers were identified based on the effect of through-space H–F coupling [26,27]:

(38a) 1-Bromo-4-methyl-2-(trifluoromethyl)benzene

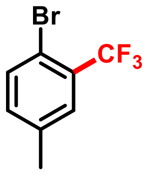

^{19}F NMR (376 MHz, DMSO-d_6/EtOAc): δ = −62.08 (s, 3F).

(38b) 4-Bromo-1-methyl-2-(trifluoromethyl)benzene

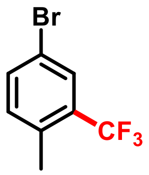

^{19}F NMR (376 MHz, DMSO-d_6/EtOAc): δ = –61.68 (q, 3F).

(39) Trifluoromethylation of 1-bromo-2-methoxybenzene (2-bromoanisole)

The reaction was carried out using 2-bromoanisole (56.1 mg, 0.3 mmol) according to the General procedure.

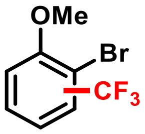

^{19}F NMR yield of mixture of isomers: 26%

(40) Trifluoromethylation of 1-bromo-3-methoxybenzene (3-bromoanisole)

The reaction was carried out using 3-bromoanisole (56.1 mg, 0.3 mmol) according to the General procedure.

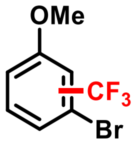

^{19}F NMR yield of mixture of isomers: 30%

(41) Trifluoromethylation of 1-bromo-4-methoxybenzene (4-bromoanisole)

The reaction was carried out using 4-bromoanisole (56.1 mg, 0.3 mmol) according to the General procedure.

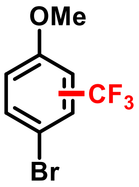

^{19}F NMR yield of mixture of isomers: 22%

(42) Trifluoromethylation of 4-bromo-1-methoxy-2-methylbenzene

The reaction was carried out using 4-bromo-1-methoxy-2-methylbenzene (60.3 mg, 0.3 mmol) according to the General procedure.

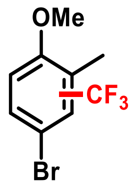

^{19}F NMR yield of mixture of isomers: 12%

(43) Trifluoromethylation of 1-bromo-4-methoxy-2-methylbenzene

The reaction was carried out using 1-bromo-4-methoxy-2-methylbenzene (60.3 mg, 0.3 mmol) according to the General procedure.

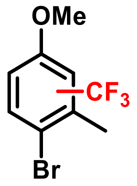

^{19}F NMR yield of mixture of isomers: 42%

(44) Trifluoromethylation of 5-bromo-2-methoxy-1,3-dimethylbenzene

The reaction was carried out using 5-bromo-2-methoxy-1,3-dimethylbenzene (64.5 mg, 0.3 mmol) according to the General procedure.

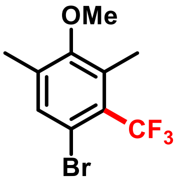

1-Bromo-4-methoxy-3,5-dimethyl-2-(trifluoromethyl)benzene

^{19}F NMR yield: 22%

The product (31) was purified by column chromatography (eluent: 100% CyH) and characterized**:**

^{1}H NMR (400 MHz, $CDCl_3$): δ 7.40 (s, 1H), 3.69 (s, 3H), 2.43 (q, *J* = 3.4 Hz, 3H), 2.29 (s, 3H).

^{19}F NMR (376 MHz, DMSO-*d*$_6$/EtOAc): δ = –54.56 (q, J = 3.5 Hz, 3F).

^{13}C NMR ((101 MHz, $CDCl_3$): δ 156.97, 136.30 (q, *J* = 0.7 Hz), 135.38, 133.64 (q, *J* = 1.3 Hz), 127.37 (q, *J* = 29.7 Hz), 123.99 (q, *J* = 276.0 Hz), 114.98 (q, *J* = 2.5 Hz), 60.19, 16.21, 13.52 (q, *J* = 4.6 Hz).

GC-MS (EI): *m*/*z* calcd for $C_{10}H_{10}BrF_3O$ $[M]^+$ 284 and 282; found 284 and 282. The product peak was observed at 21.66 min.

The EI–MS fragmentation spectra of compound (**44**):

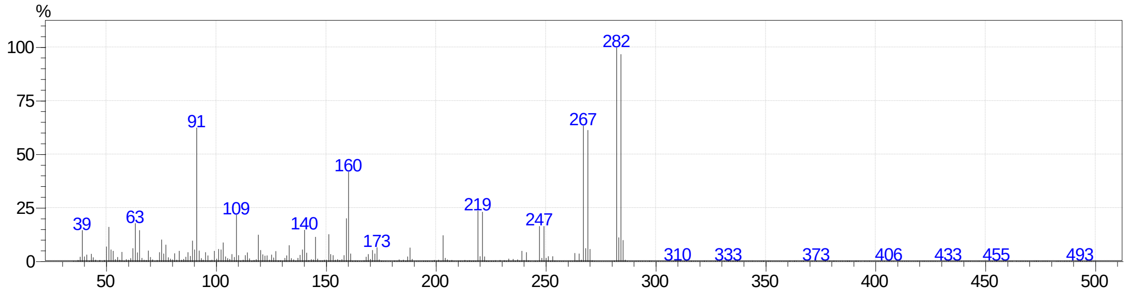

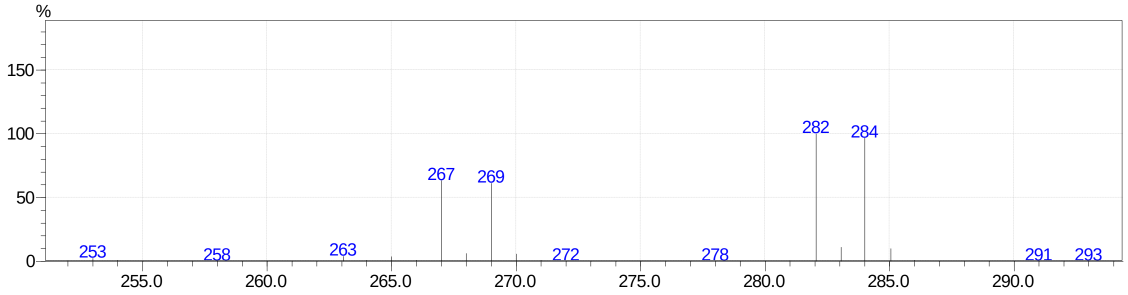

10. NMR spectra

(1) ^{19}F NMR (376 MHz, $CDCl_3$/EtOAc) for trifluoromethylation of 1,3,5-trimethoxybenzene

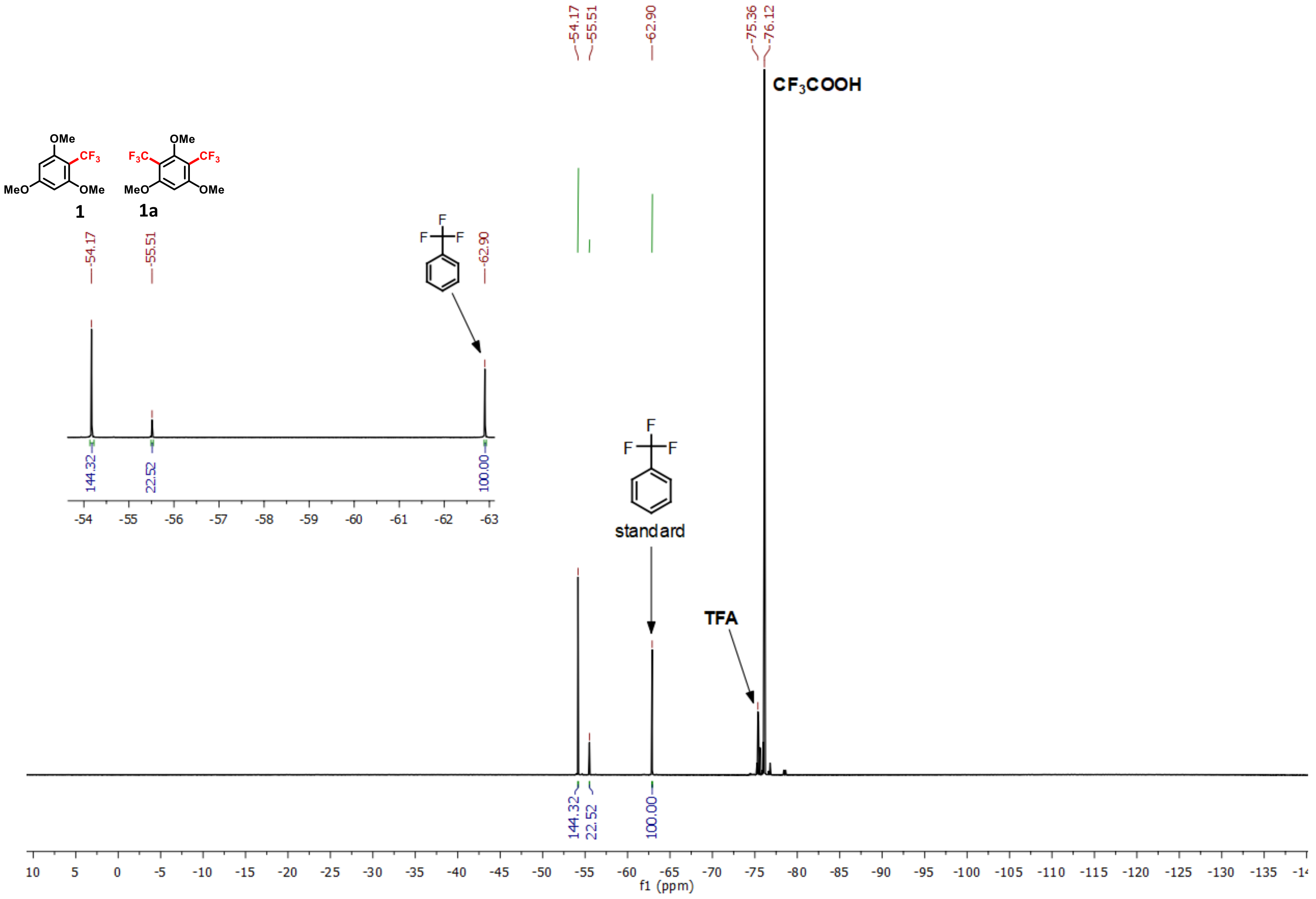

(2) ^{19}F NMR (376 MHz, DMSO-d_6/EtOAc) for trifluoromethylation of benzene

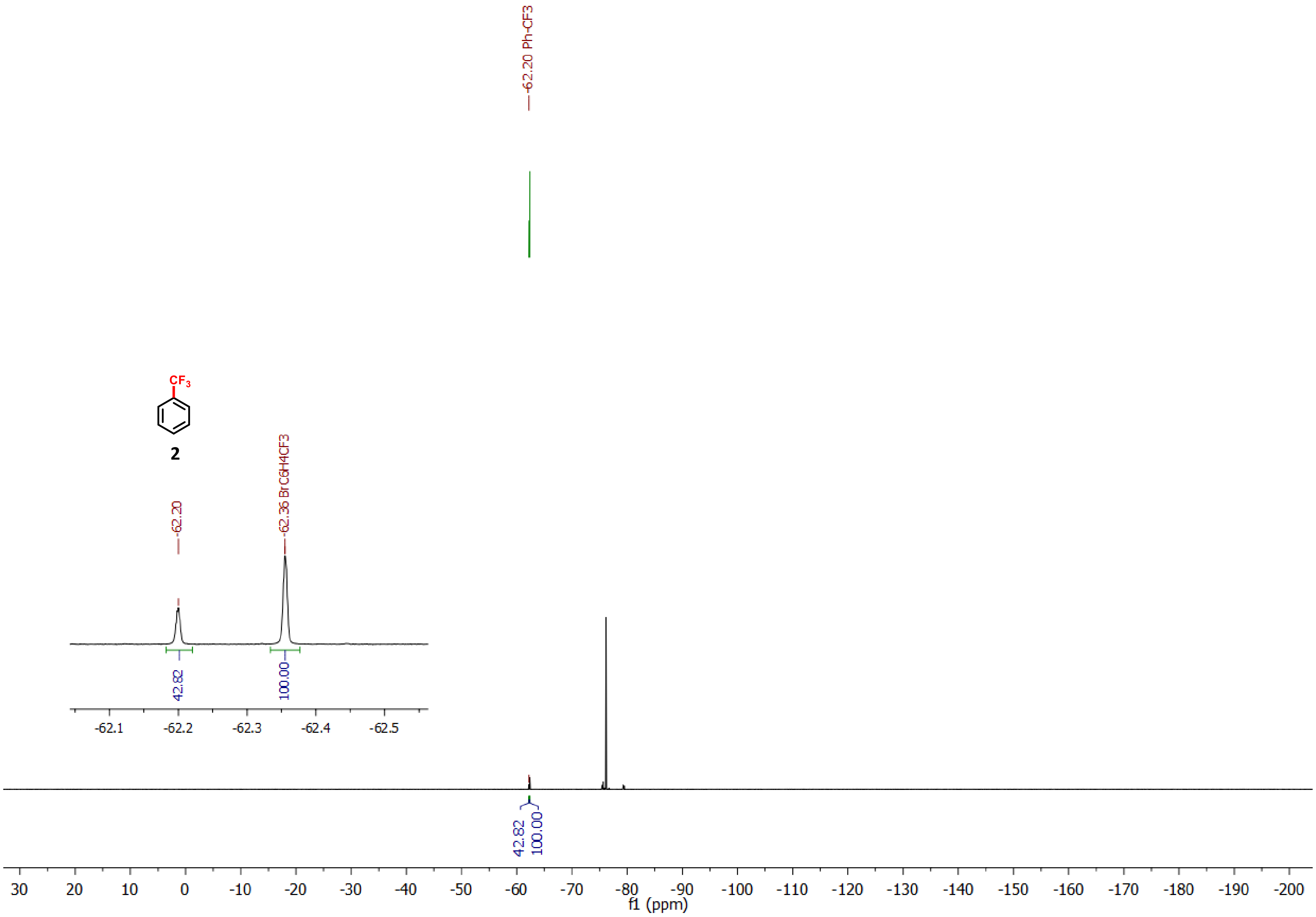

(3) ^{19}F NMR (376 MHz, DMSO-d_6/EtOAc) for trifluoromethylation of toluene

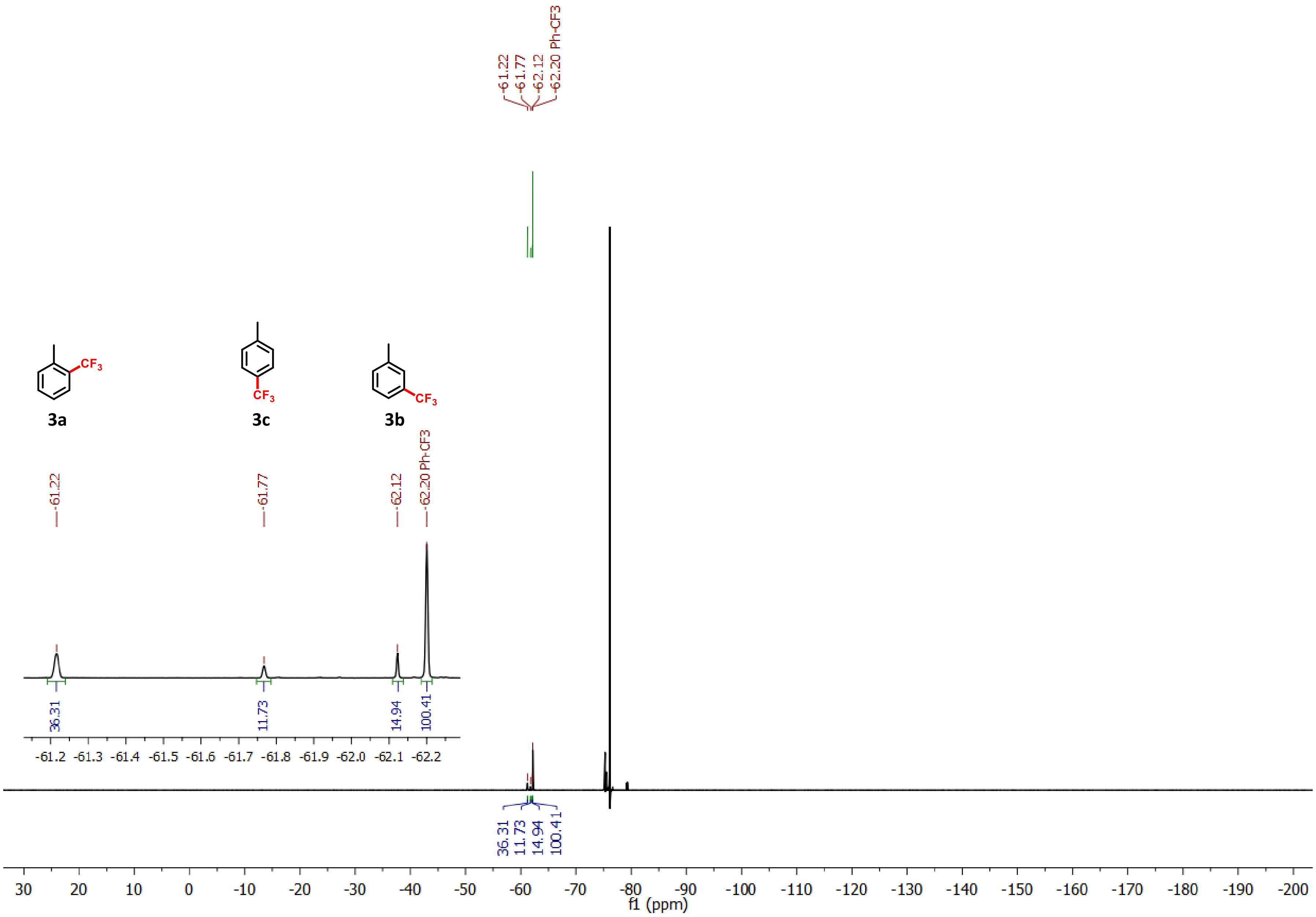

(4) ^{19}F NMR (376 MHz, DMSO-d_6/EtOAc) for trifluoromethylation of *tert*-butylbenzene

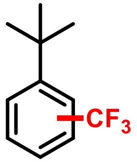

4
The mixture of isomers

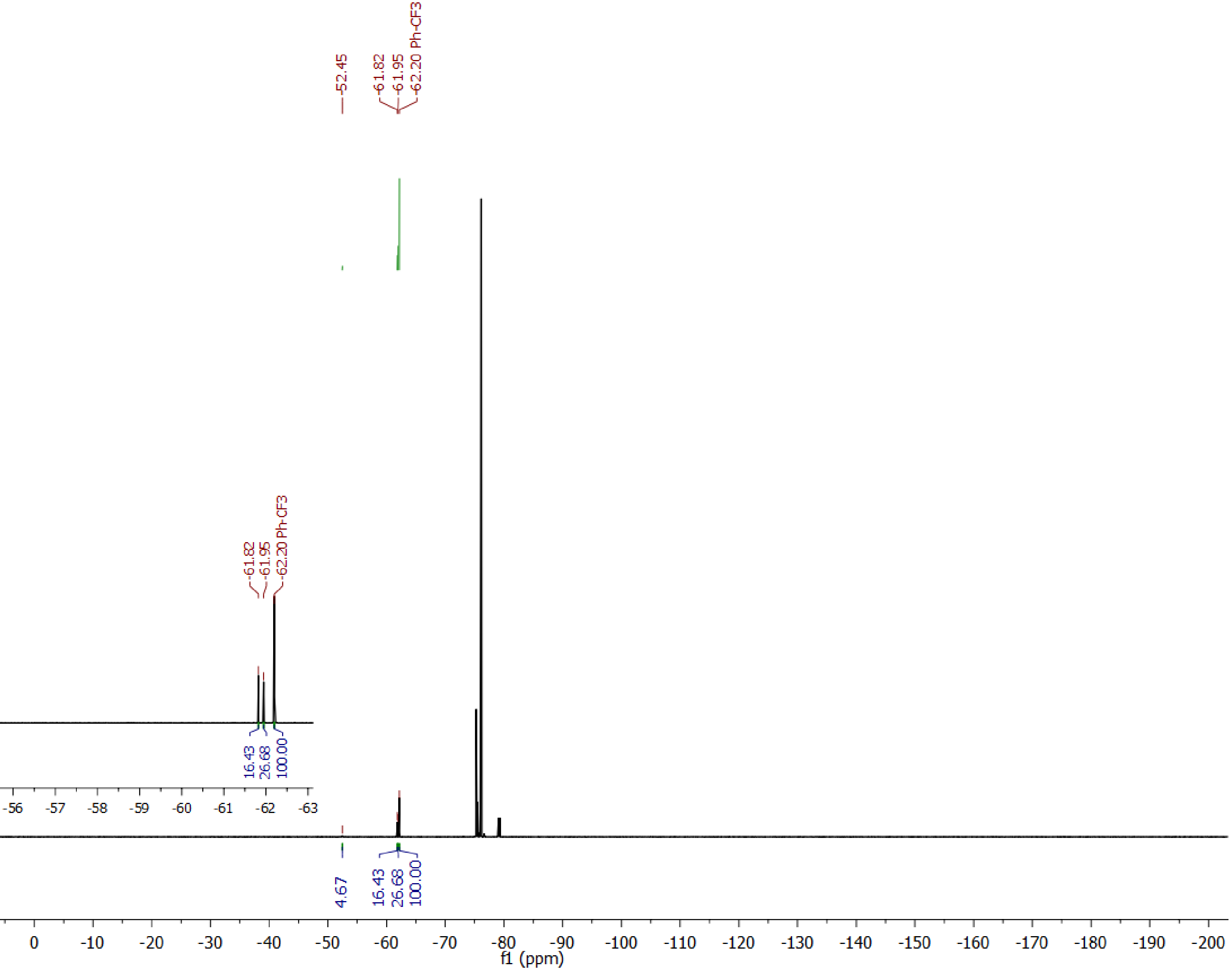

(5) ^{19}F NMR (376 MHz, DMSO-d_6/EtOAc) for trifluoromethylation of biphenyl

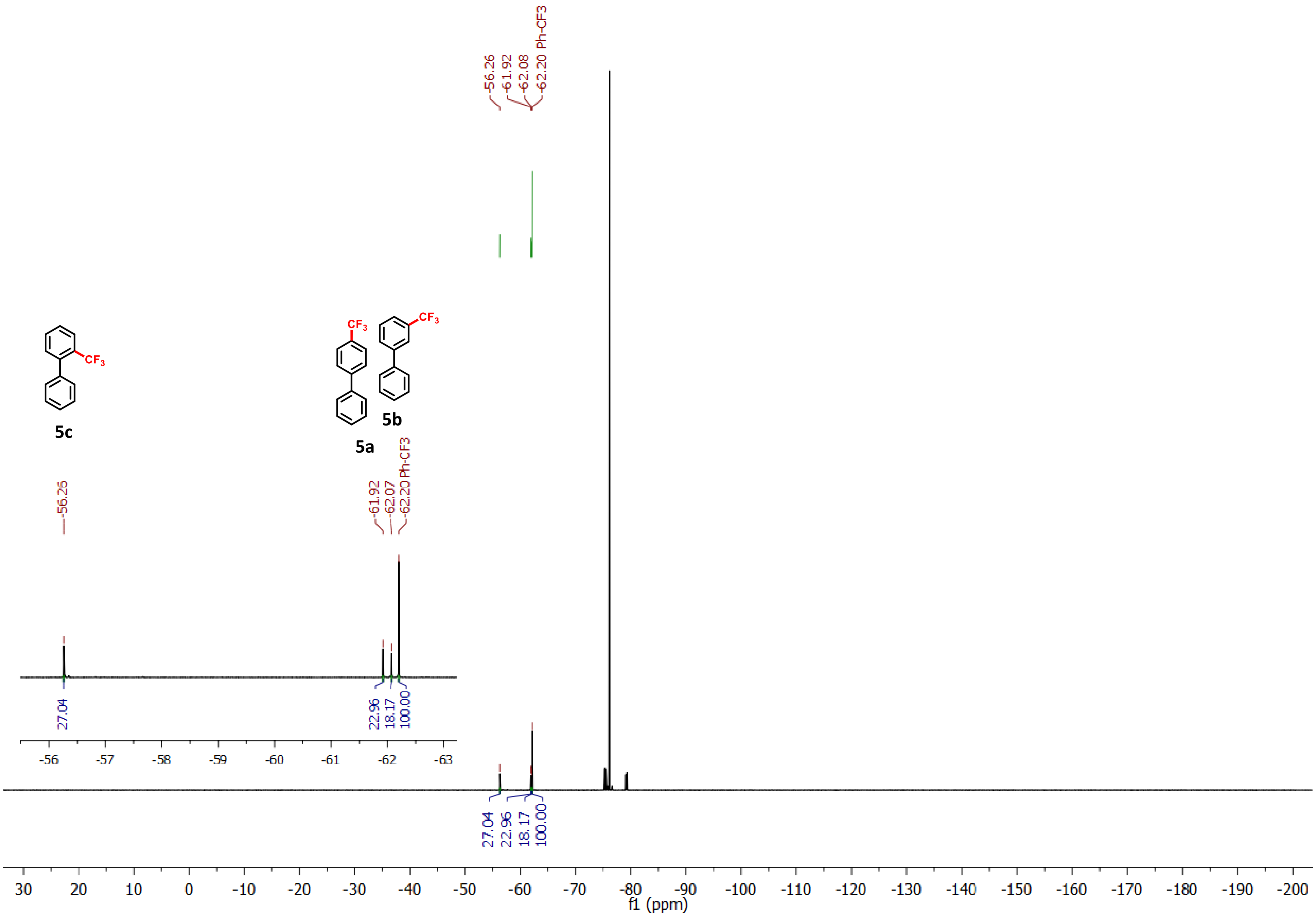

(6) ^{19}F NMR (376 MHz, DMSO-d_6/EtOAc) for trifluoromethylation of naphthalene

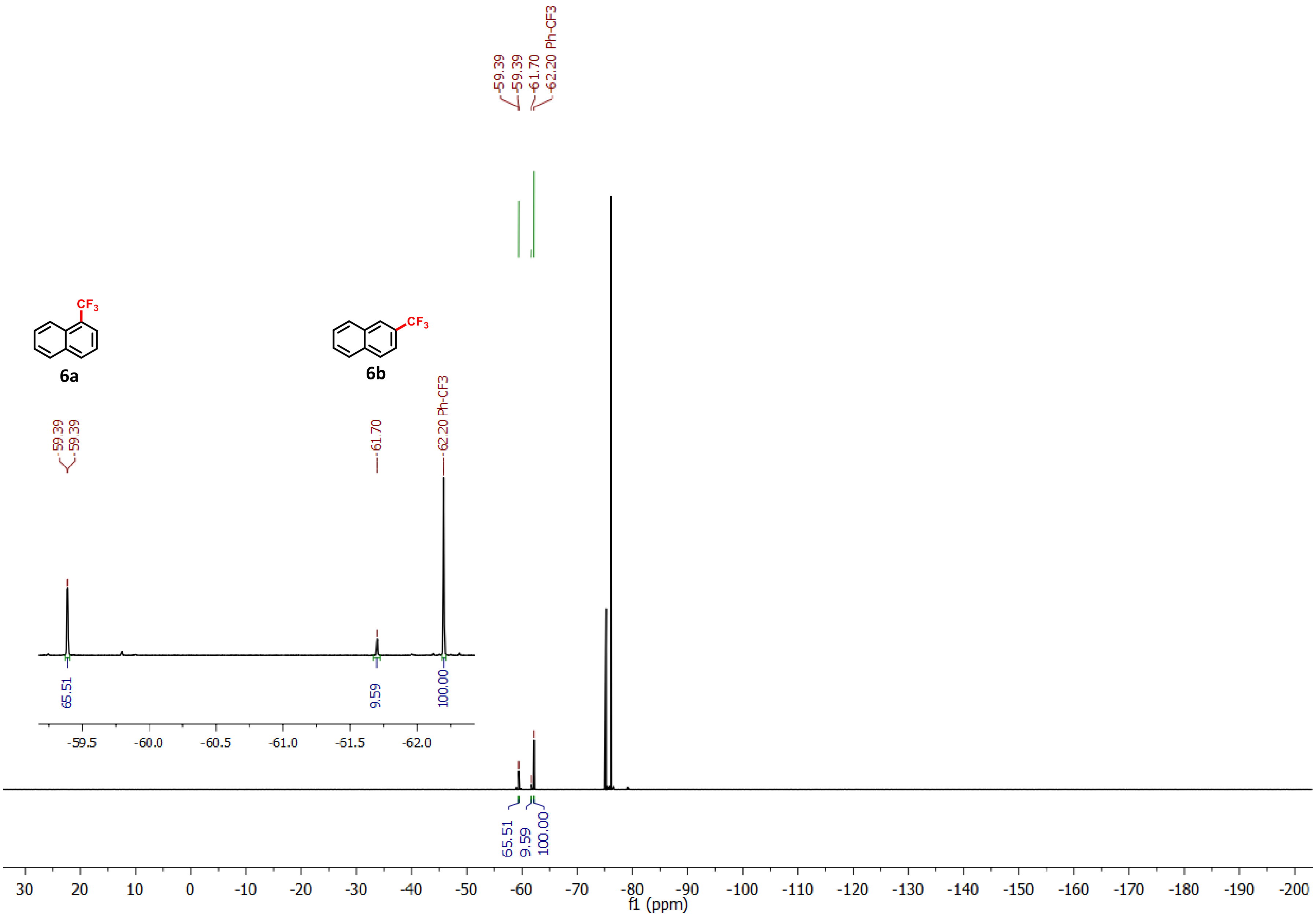

(7) ^{19}F NMR (376 MHz, DMSO-d_6/EtOAc) for trifluoromethylation of 1,2-dimethylbenzene (*o*-xylene)

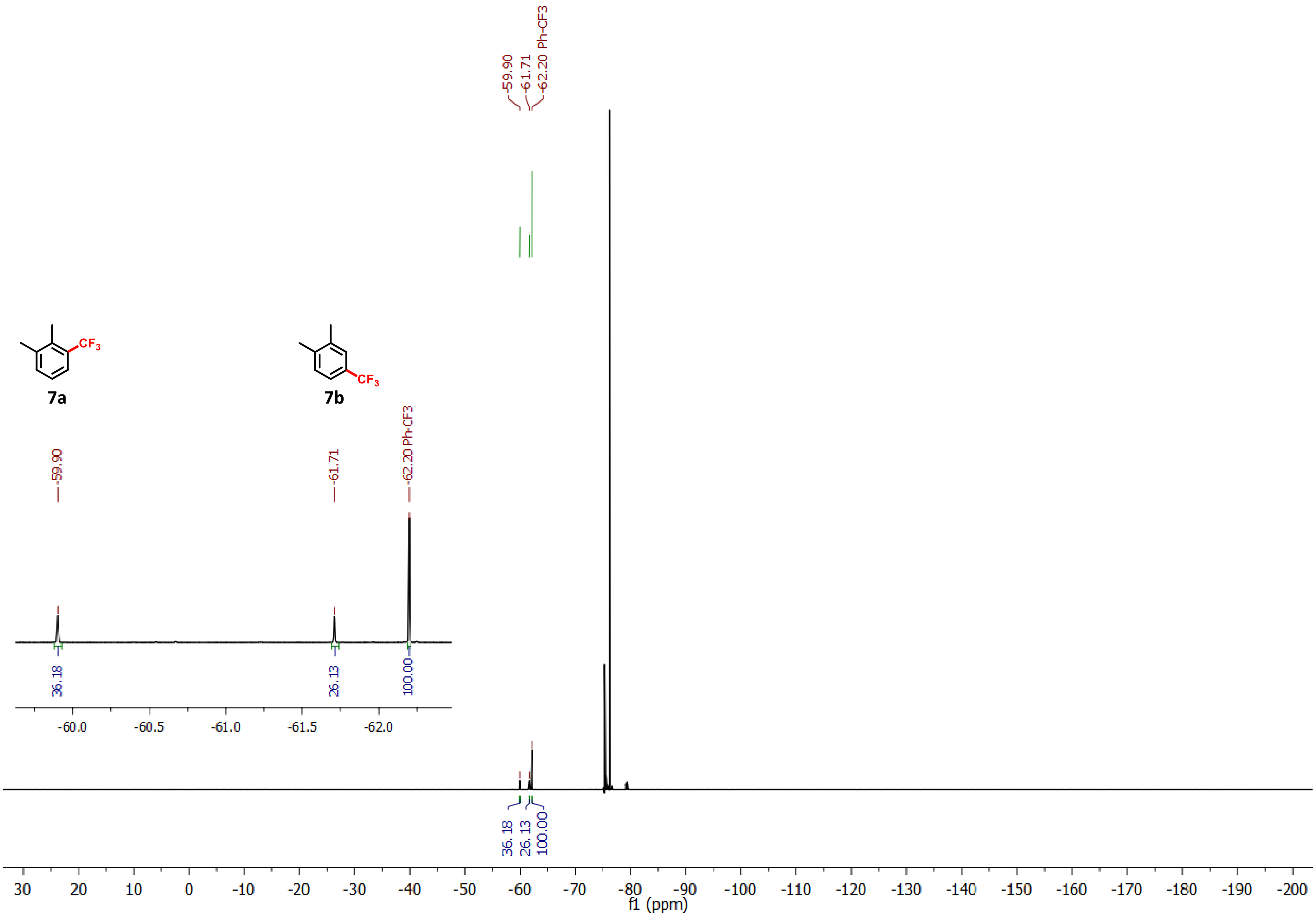

(8) ^{19}F NMR (376 MHz, DMSO-d_6/EtOAc) for trifluoromethylation of 1,2,4,5-tetramethylbenzene (durene)

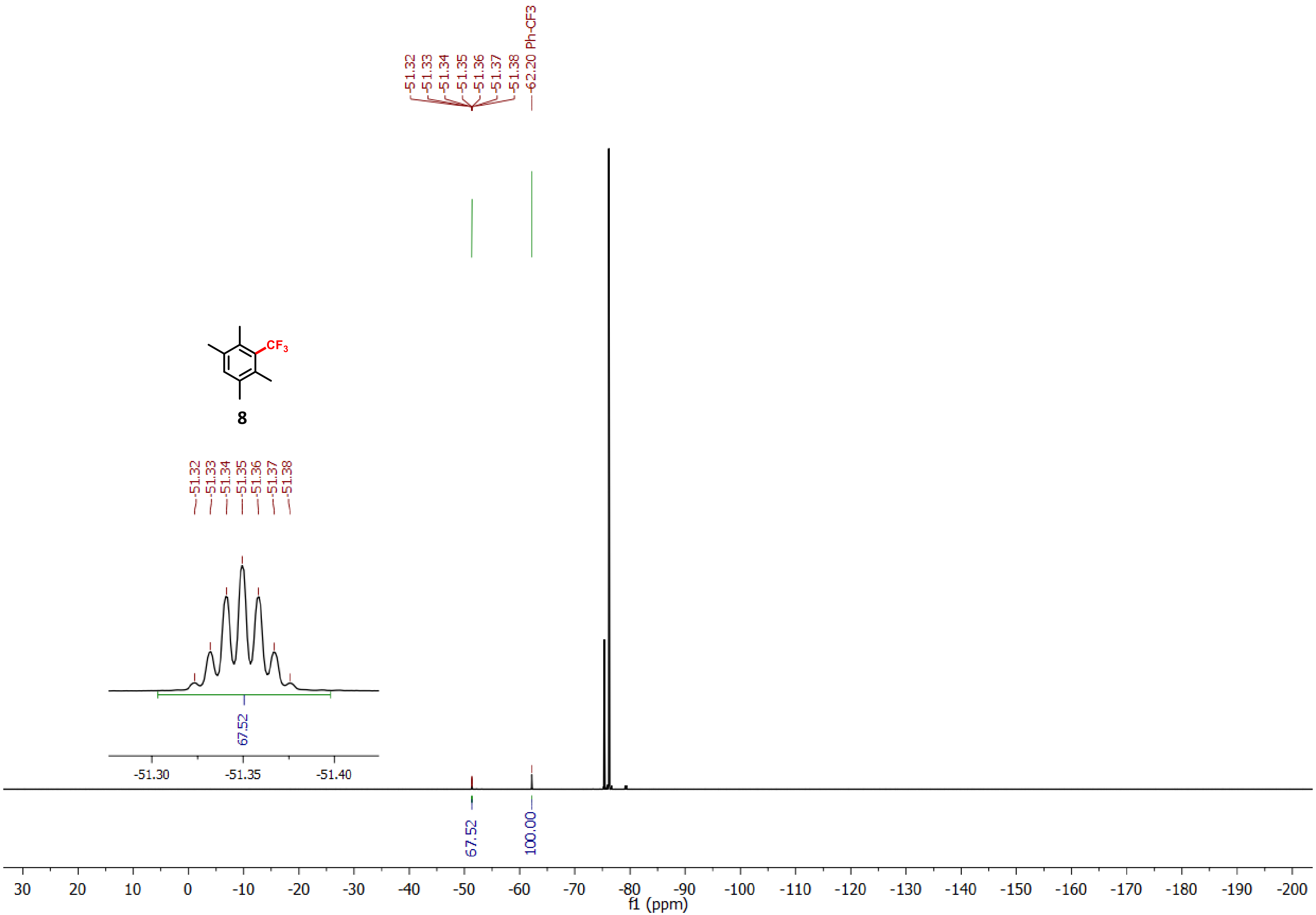

(9) ^{19}F NMR (376 MHz, $CDCl_3$/EtOAc) for trifluoromethylation of 1,3,5-trimethylbenzene (mesitylene)

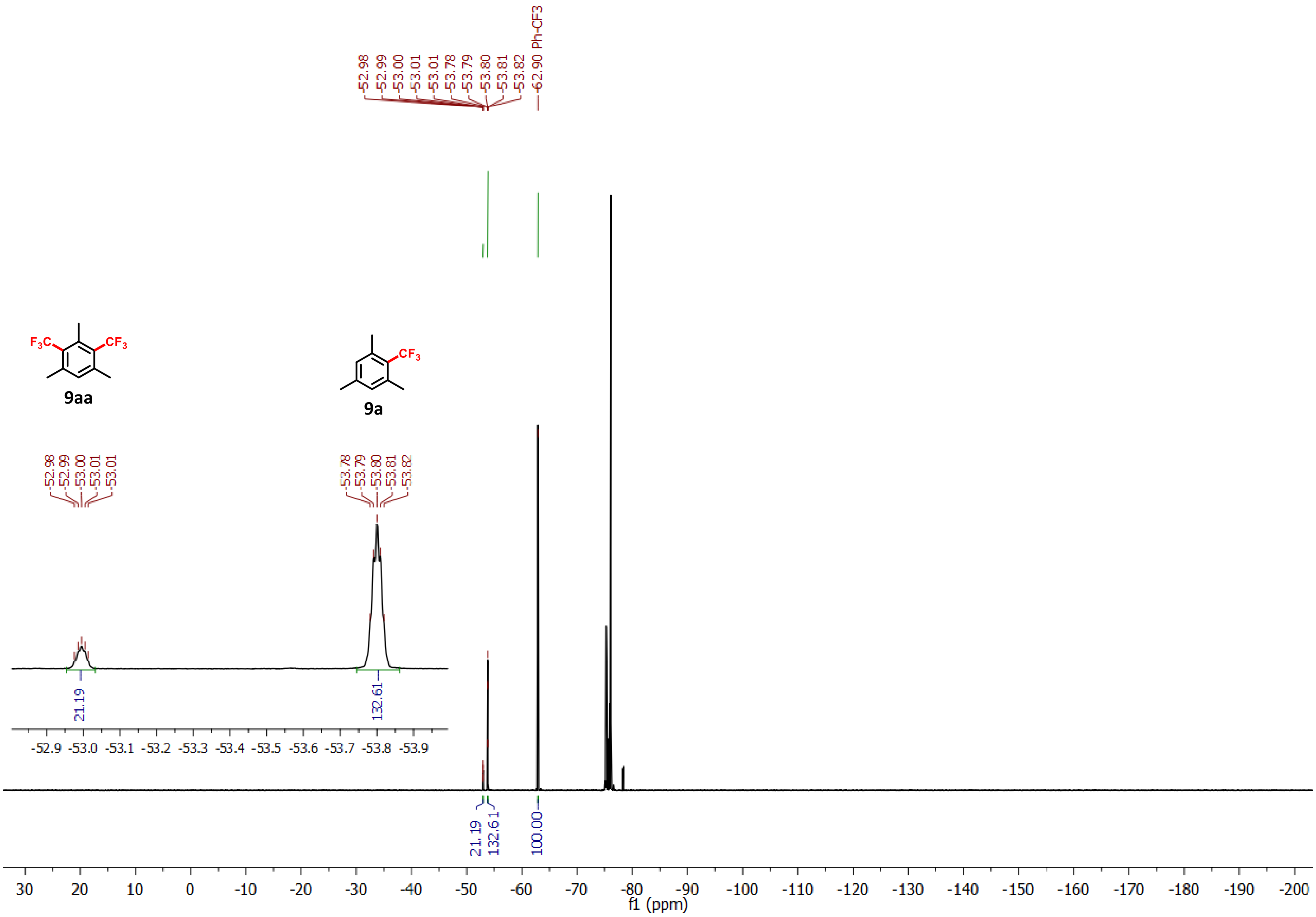

(10) ^{19}F NMR (376 MHz, DMSO-d_6/EtOAc) for trifluoromethylation of 2-bromo-1,3,5-trimethylbenzene

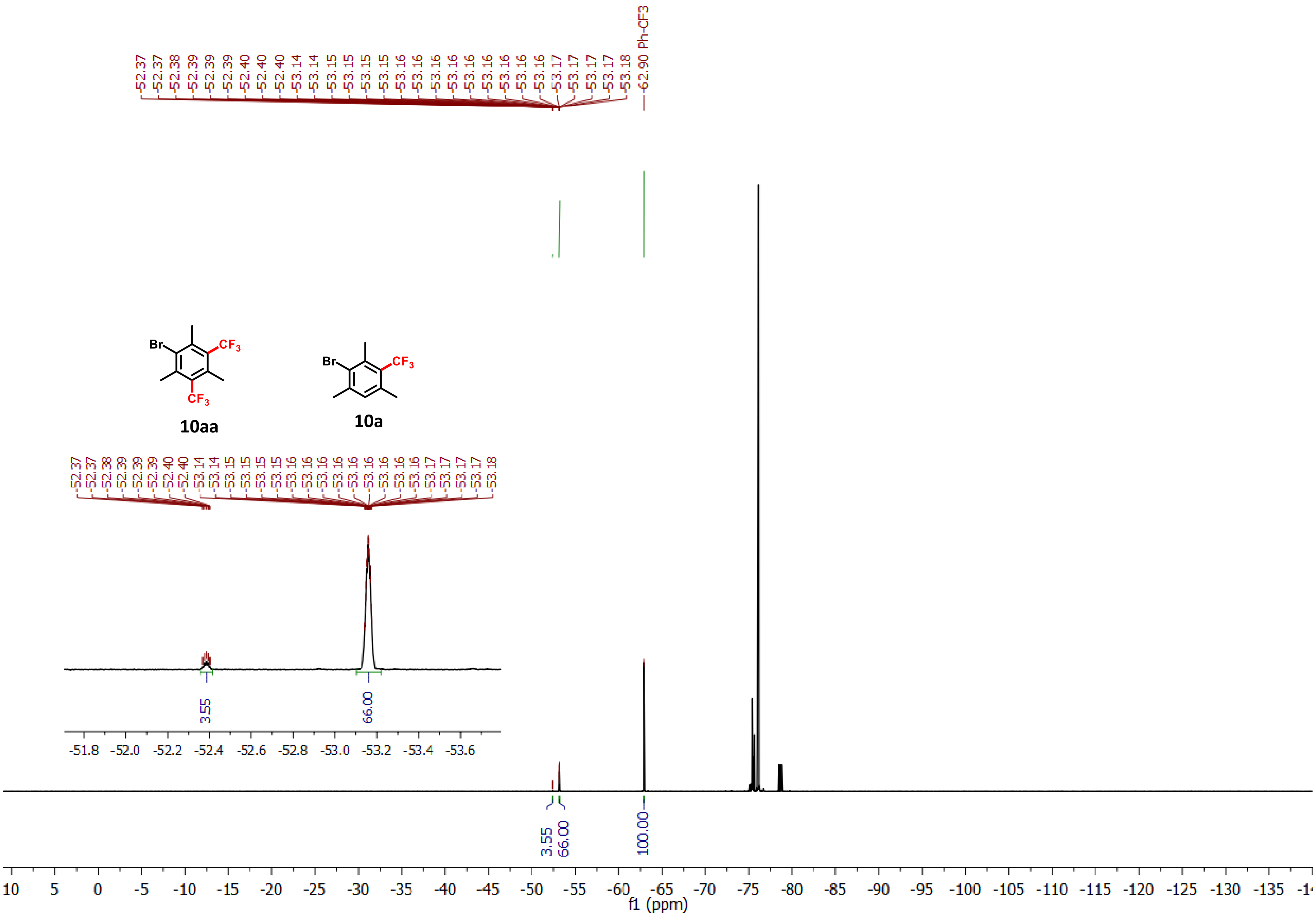

(11) ^{19}F NMR (376 MHz, $CDCl_3$/EtOAc) for trifluoromethylation of methoxybenzene (anisole)

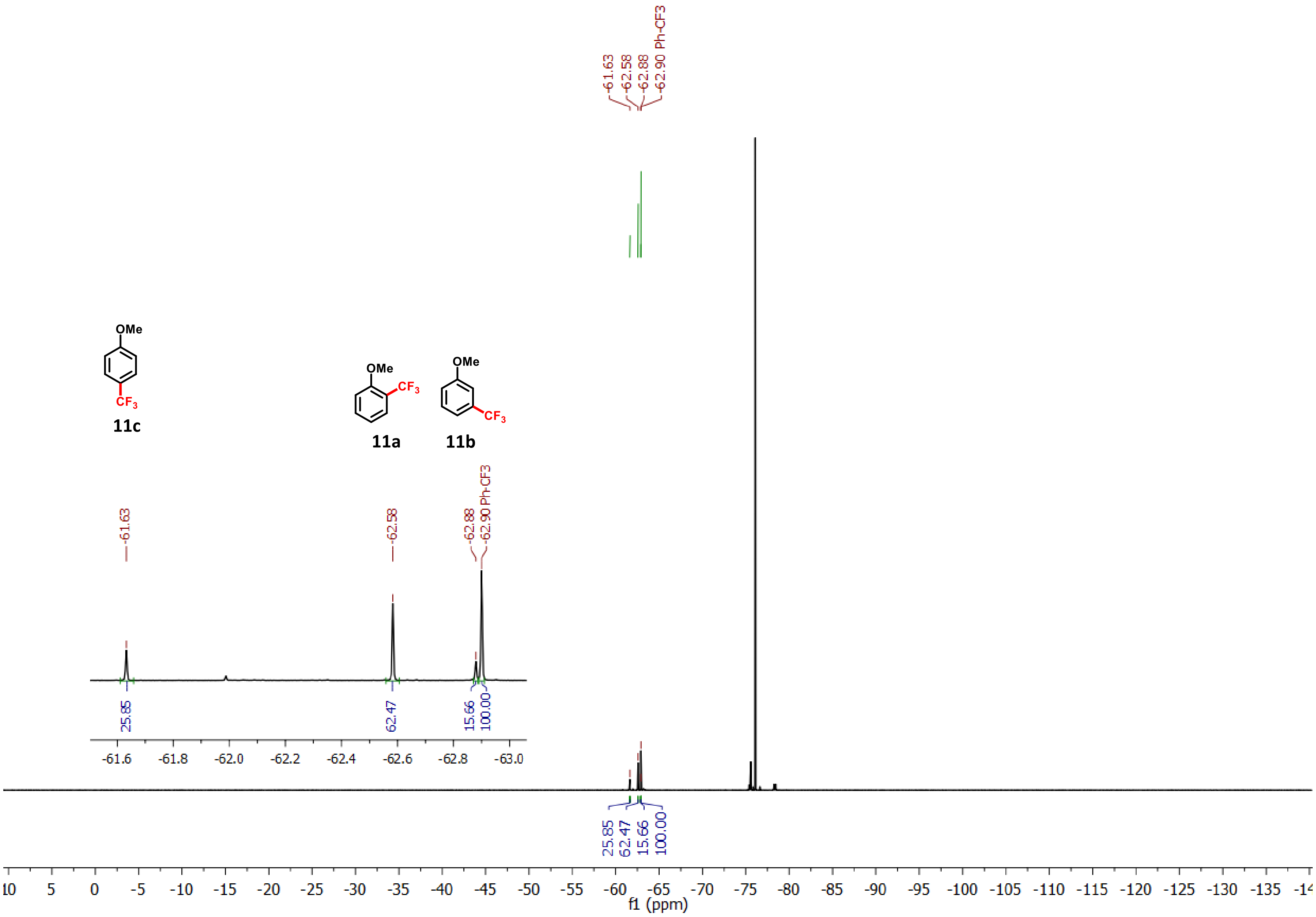

(12) ^{19}F NMR (376 MHz, $CDCl_3$/EtOAc) for trifluoromethylation of thioanisole

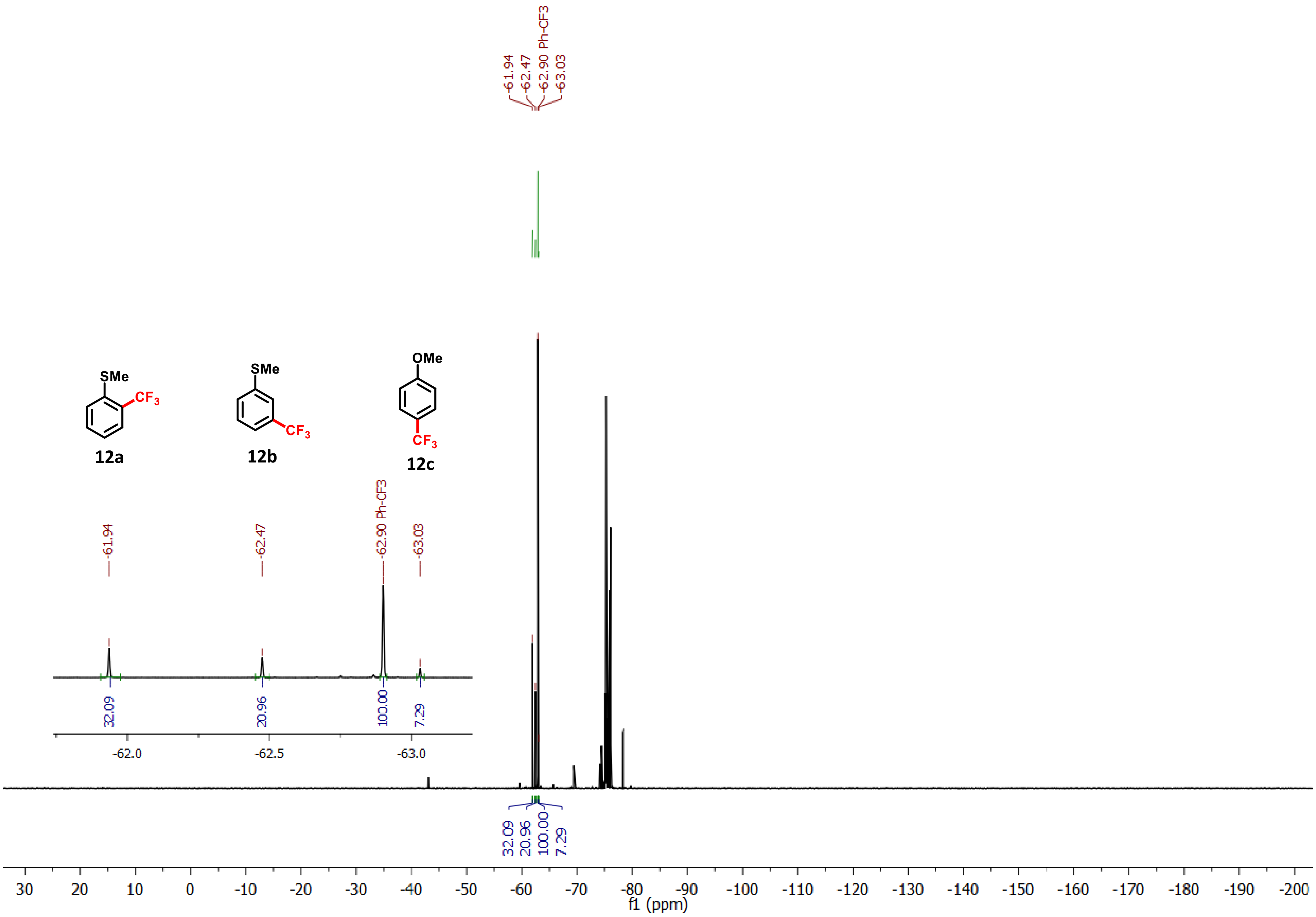

(13) ^{19}F NMR (376 MHz, DMSO-d_6/EtOAc) for trifluoromethylation of 1-methoxy-3-methylbenzene

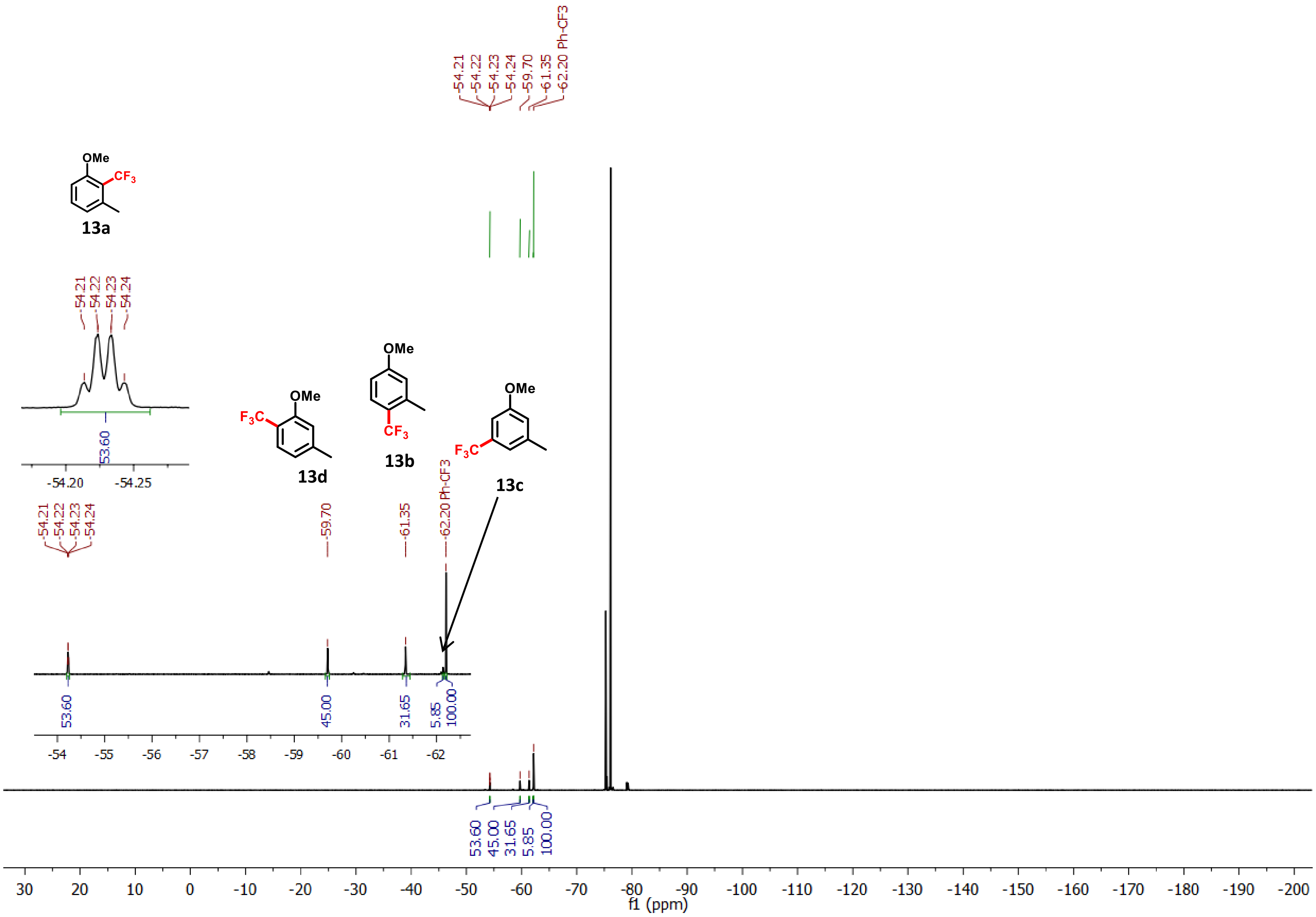

(14) ^{19}F NMR (376 MHz, DMSO-d_6/EtOAc) for trifluoromethylation of 1-methoxy-4-methylbenzene (4-methylanisole)

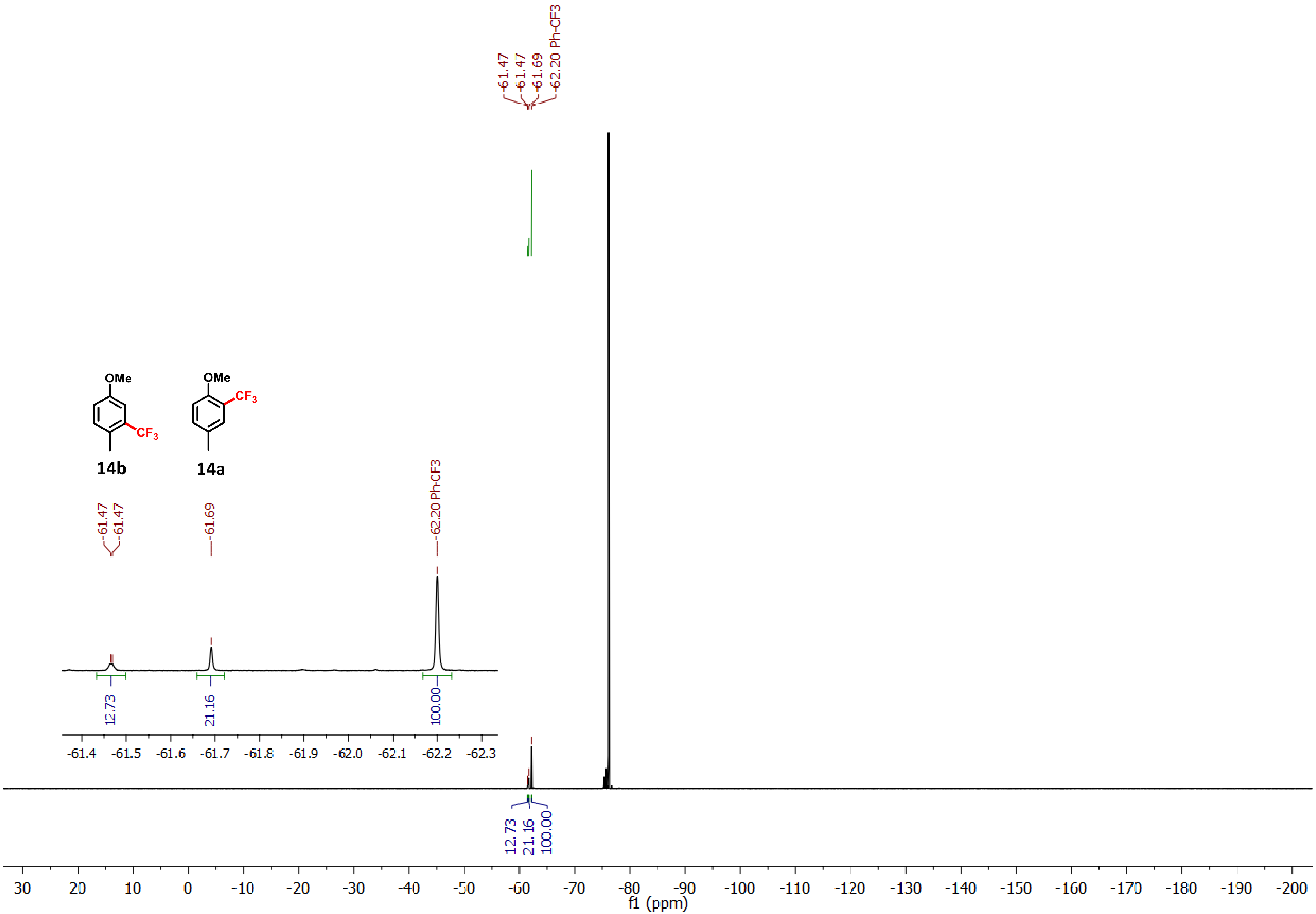

(15) ^{19}F NMR (376 MHz, DMSO-d_6/EtOAc) for trifluoromethylation of 3,4-dimethylanisole

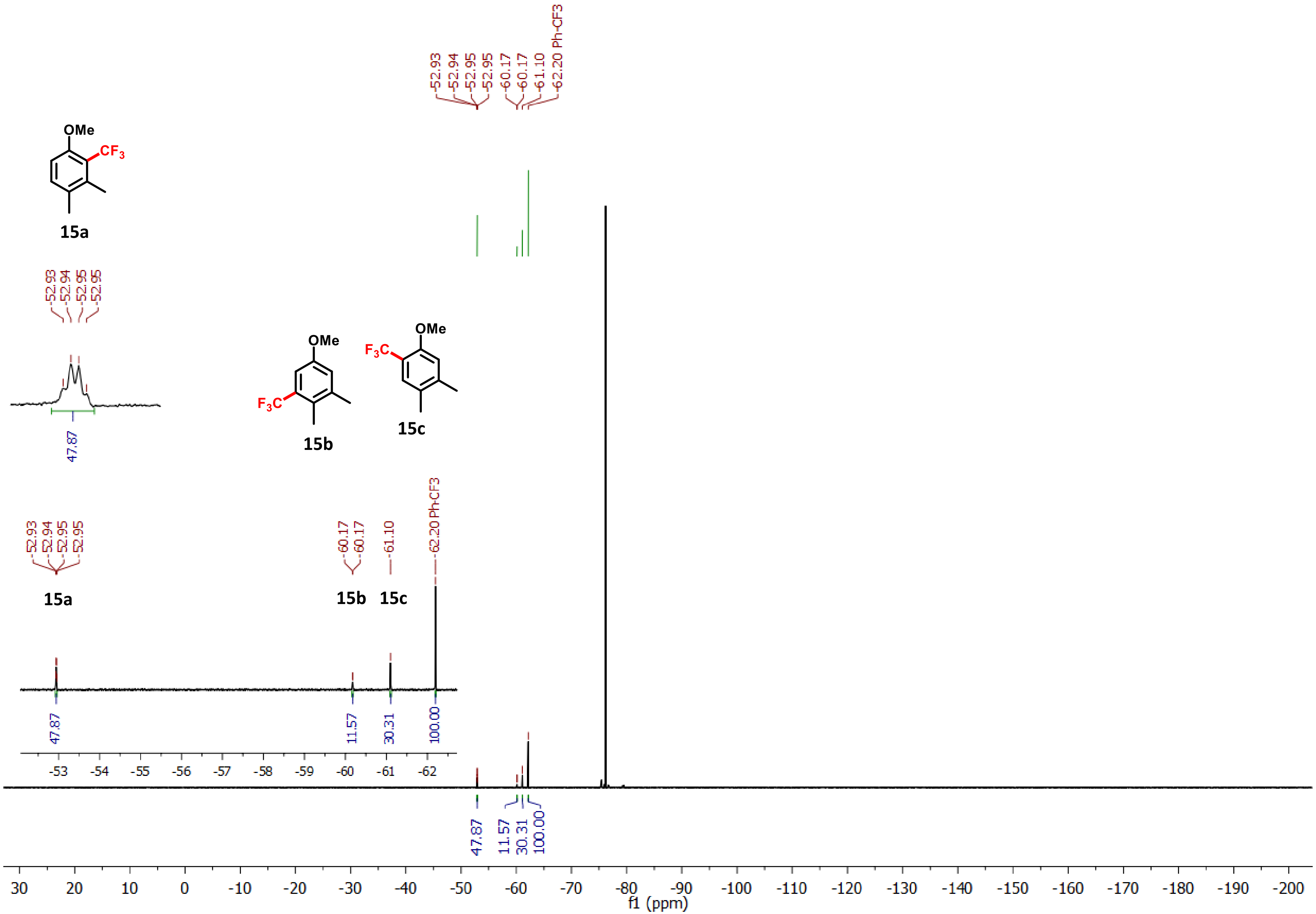

(16) ^{19}F NMR (376 MHz, DMSO-d_6/EtOAc) for trifluoromethylation of 1-isopropyl-2-methoxy-4-methylbenzene

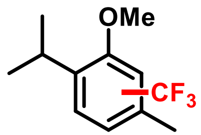

16
The mixture of isomers

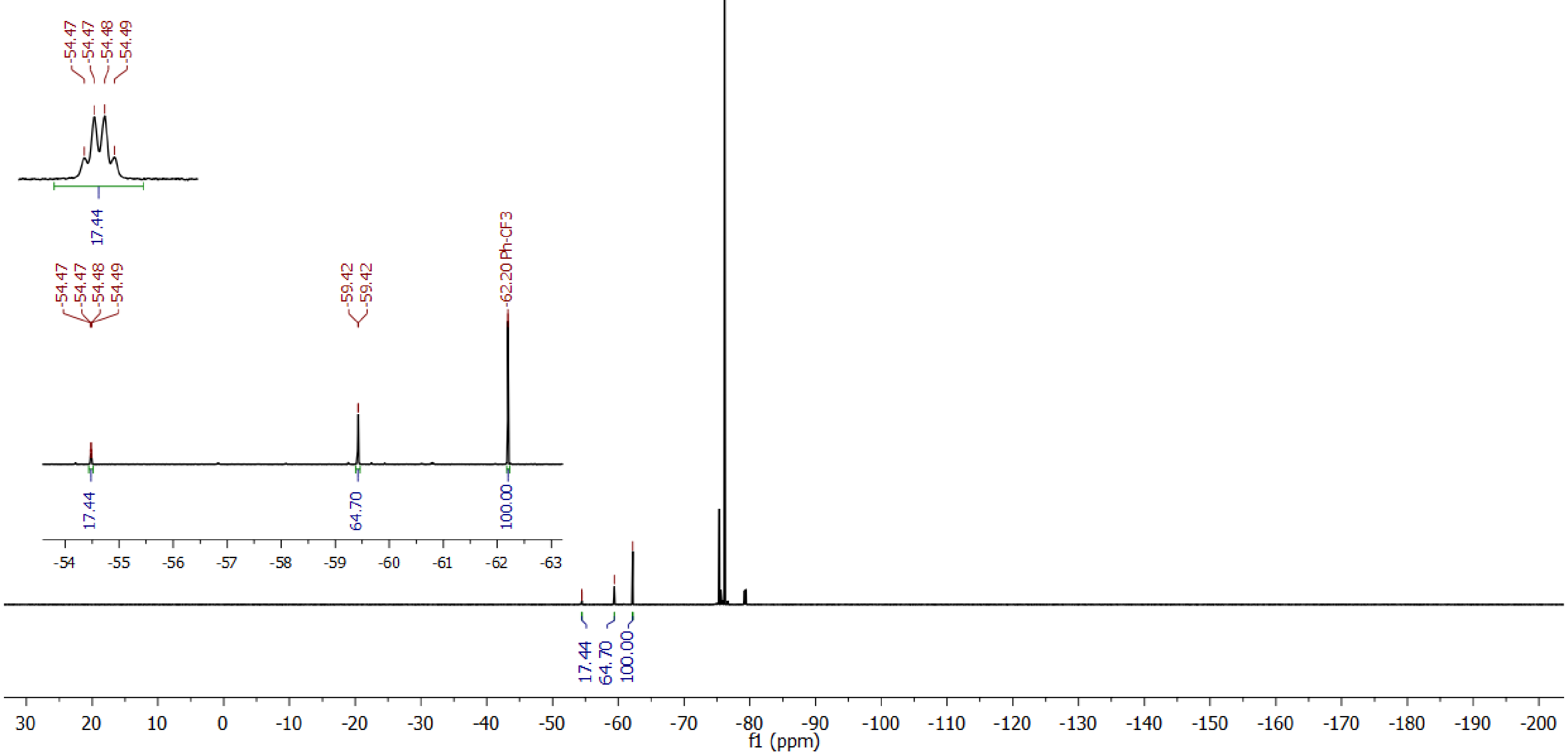

(17) ^{19}F NMR (376 MHz, $CDCl_3$/EtOAc) for trifluoromethylation of 1-methoxy-3,5-dimethylbenzene

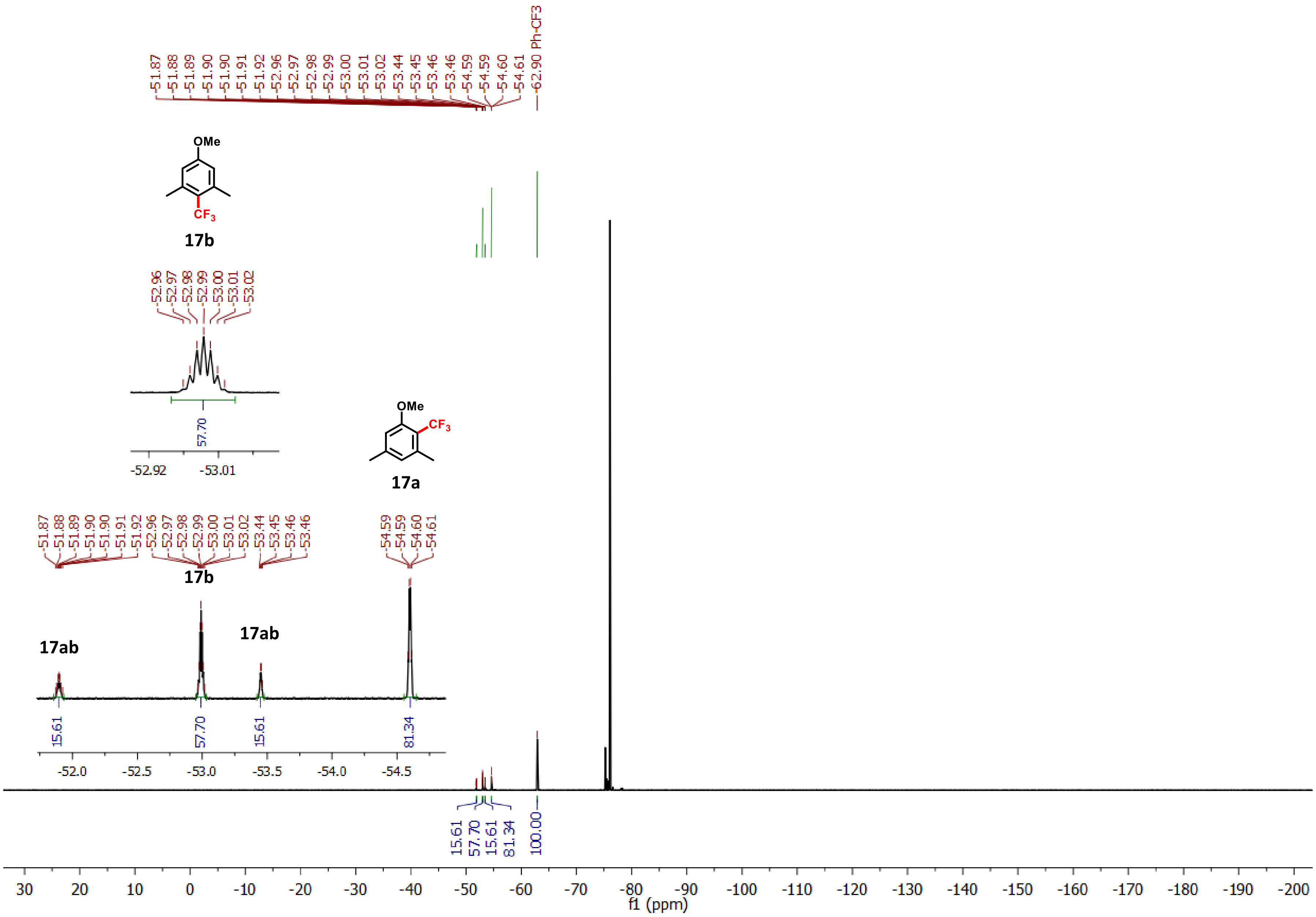

(18) ^{19}F NMR (376 MHz, DMSO-d_6/EtOAc) for trifluoromethylation of 1-methoxy-2,3,5-trimethylbenzene

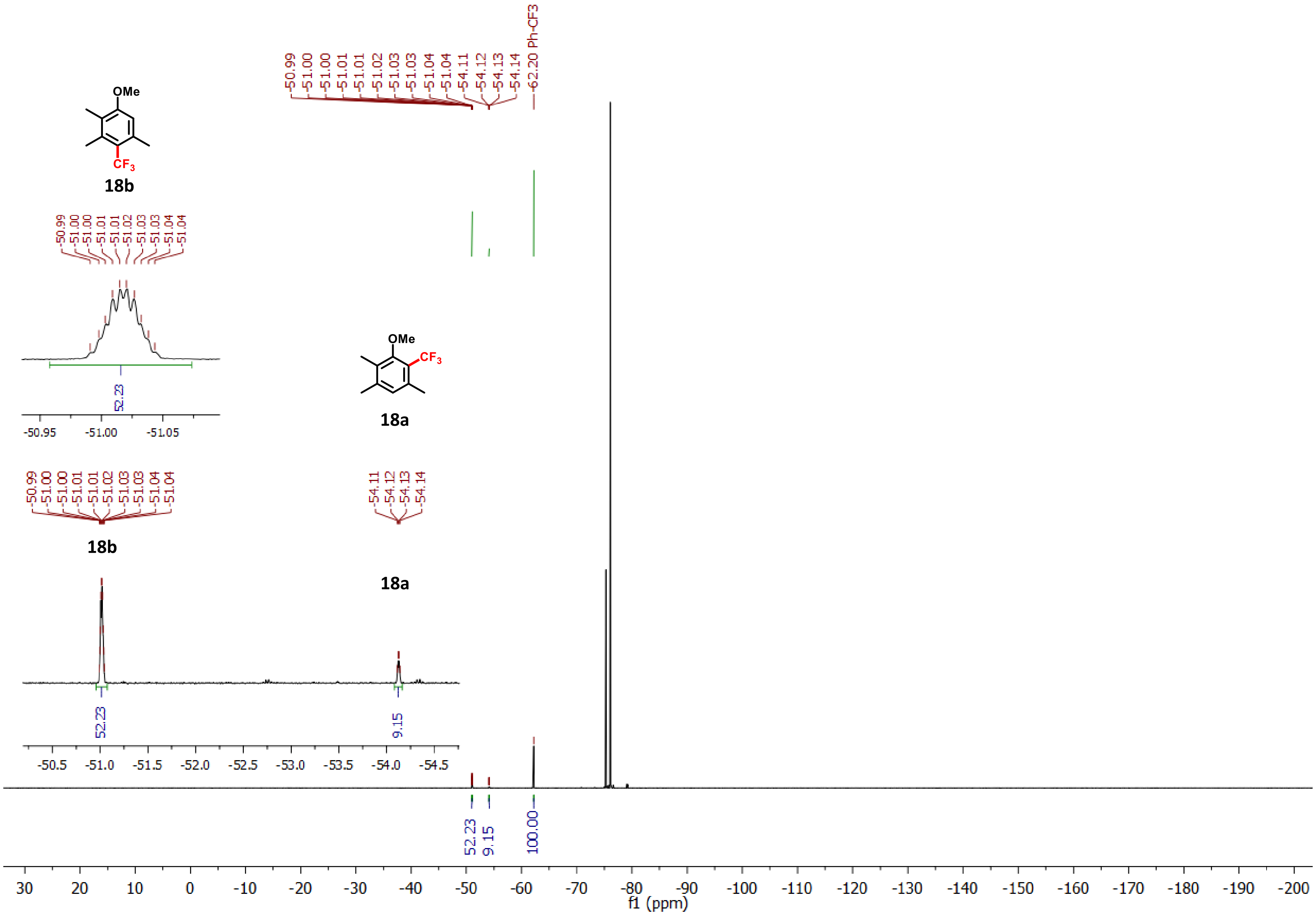

(19) ^{19}F NMR (376 MHz, DMSO-d_6/EtOAc) for trifluoromethylation of 1,2-dimethoxybenzene

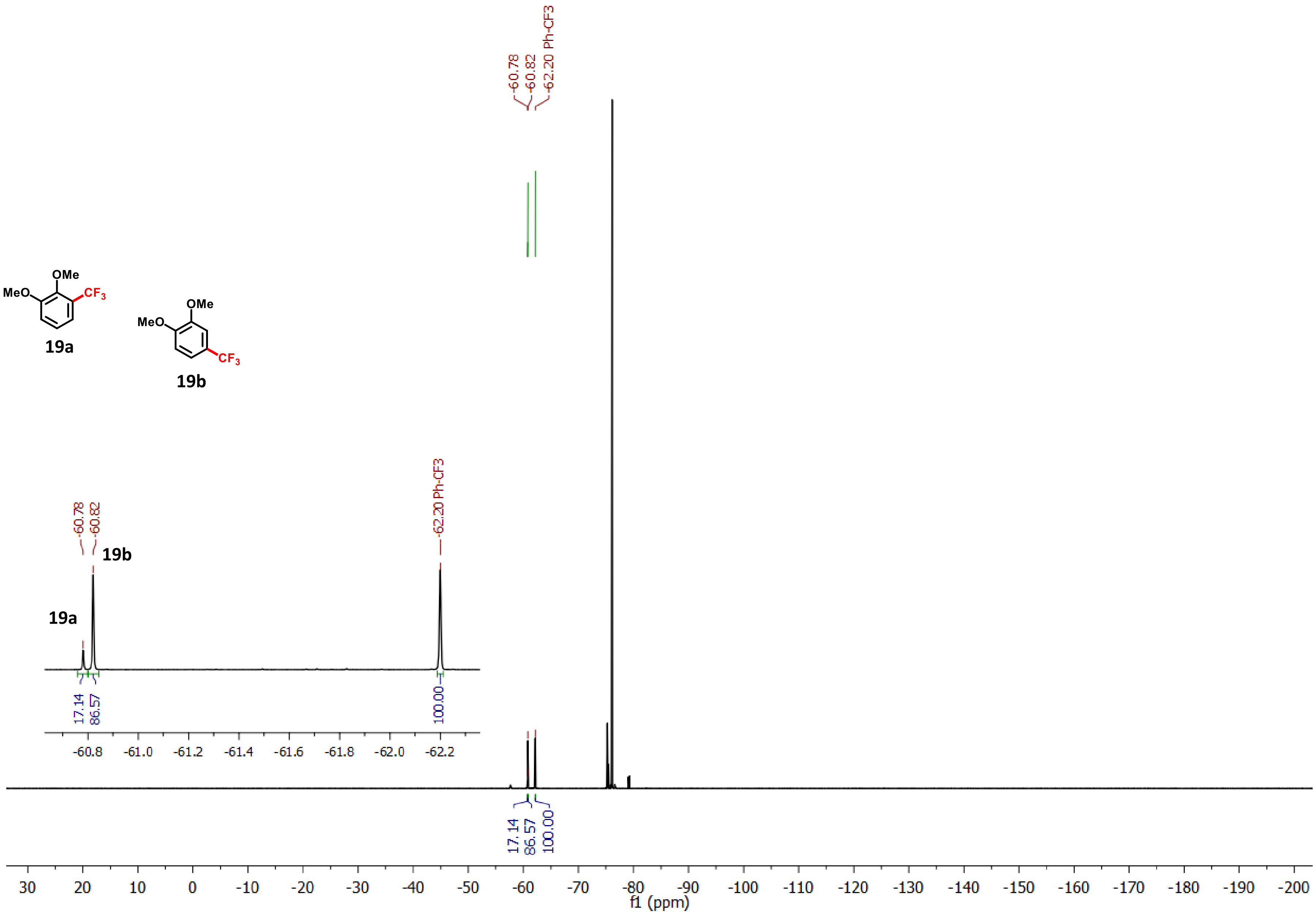

(20) ^{19}F NMR (376 MHz, DMSO-d_6/EtOAc) for trifluoromethylation of 1,3-dimethoxybenzene

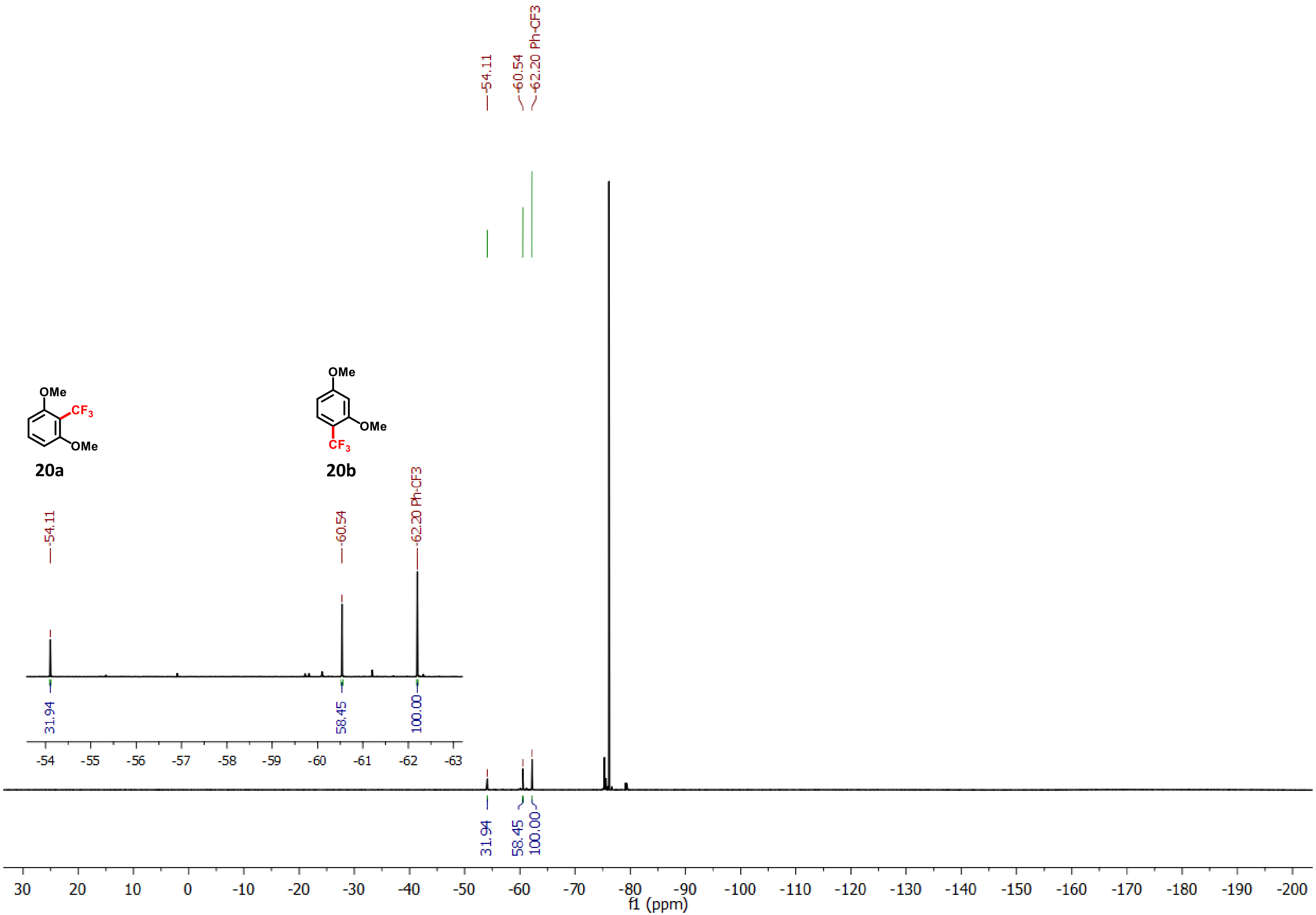

(21) ^{19}F NMR (376 MHz, DMSO-d_6/EtOAc) for trifluoromethylation of 1,4-dimethoxybenzene

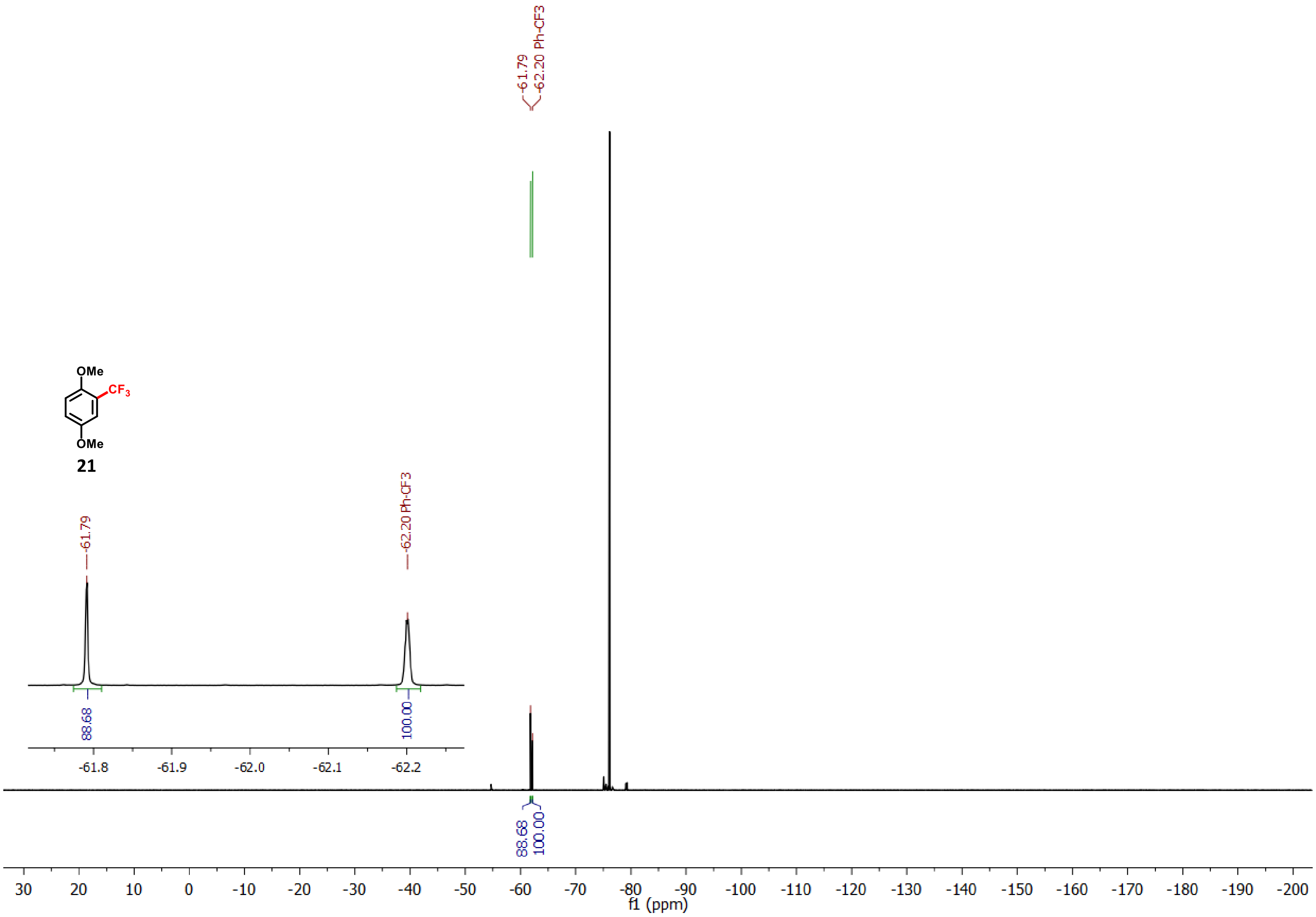

(22) ^{19}F NMR (376 MHz, DMSO-d_6/EtOAc) for trifluoromethylation of 3,5-dimethoxytoluene

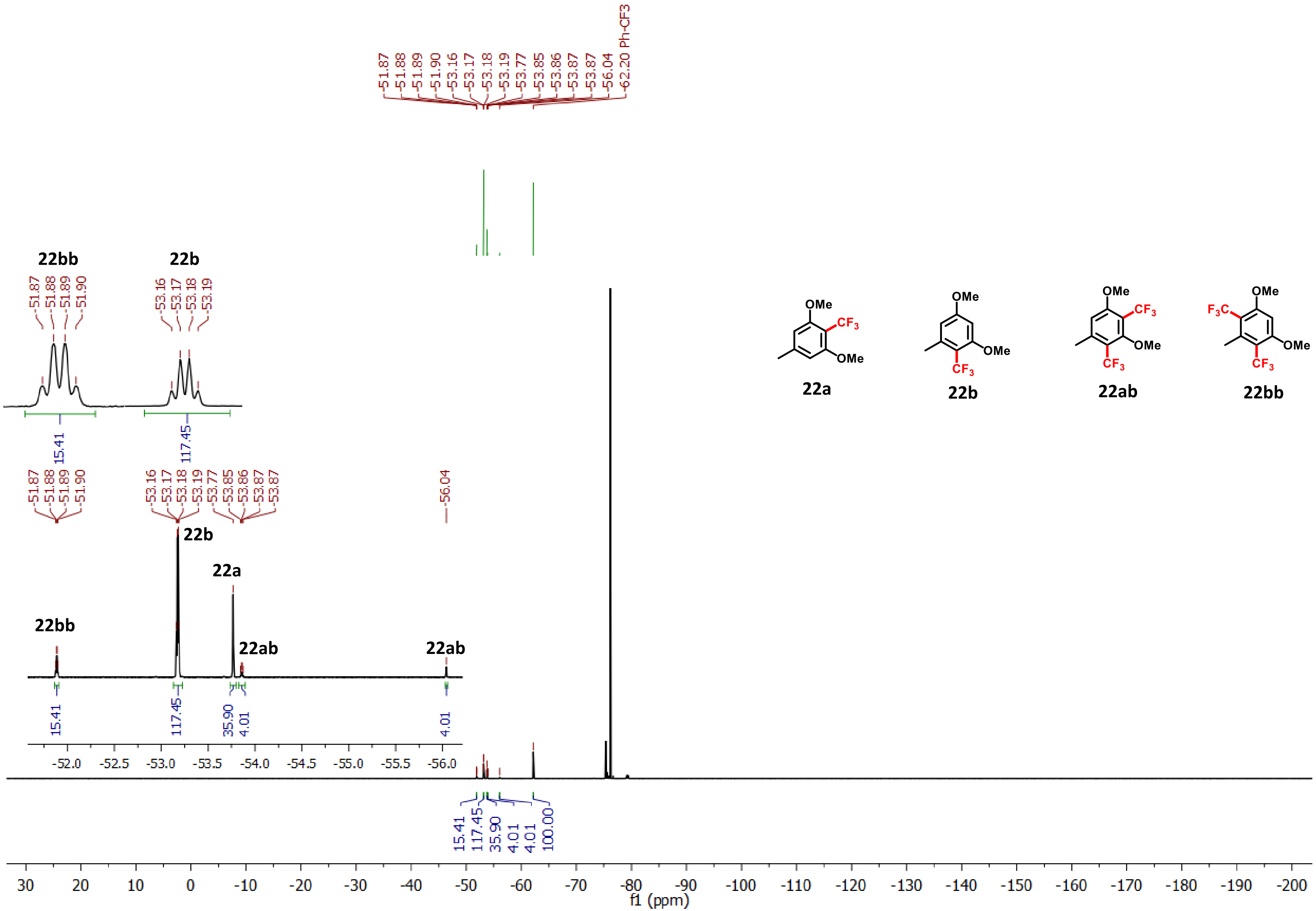

(23) ^{19}F NMR (376 MHz, DMSO-d_6/EtOAc) for trifluoromethylation of 1,2,3-trimethoxybenzene

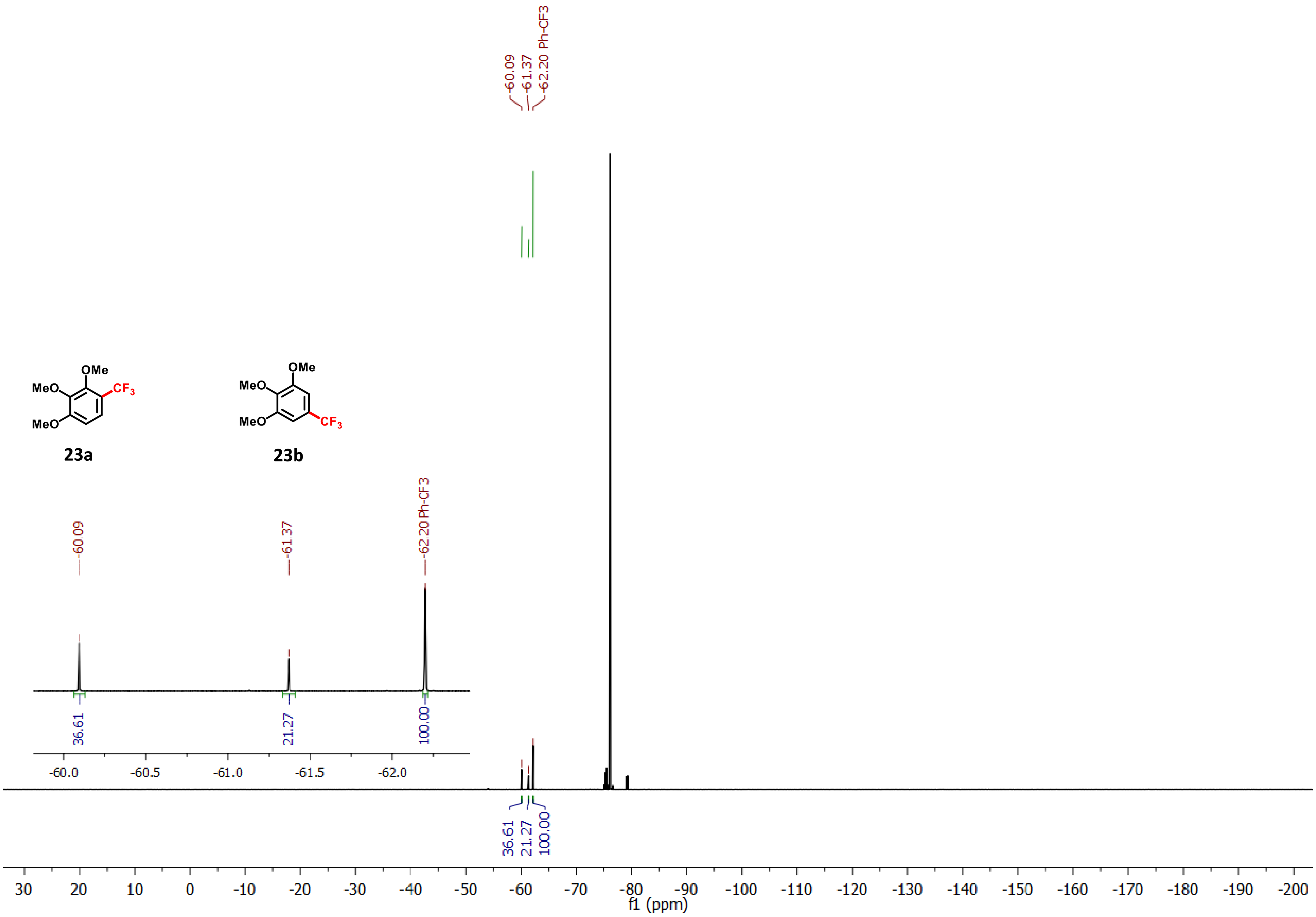

(24) ^{19}F NMR (376 MHz, DMSO-d_6/EtOAc) for trifluoromethylation of acetophenone

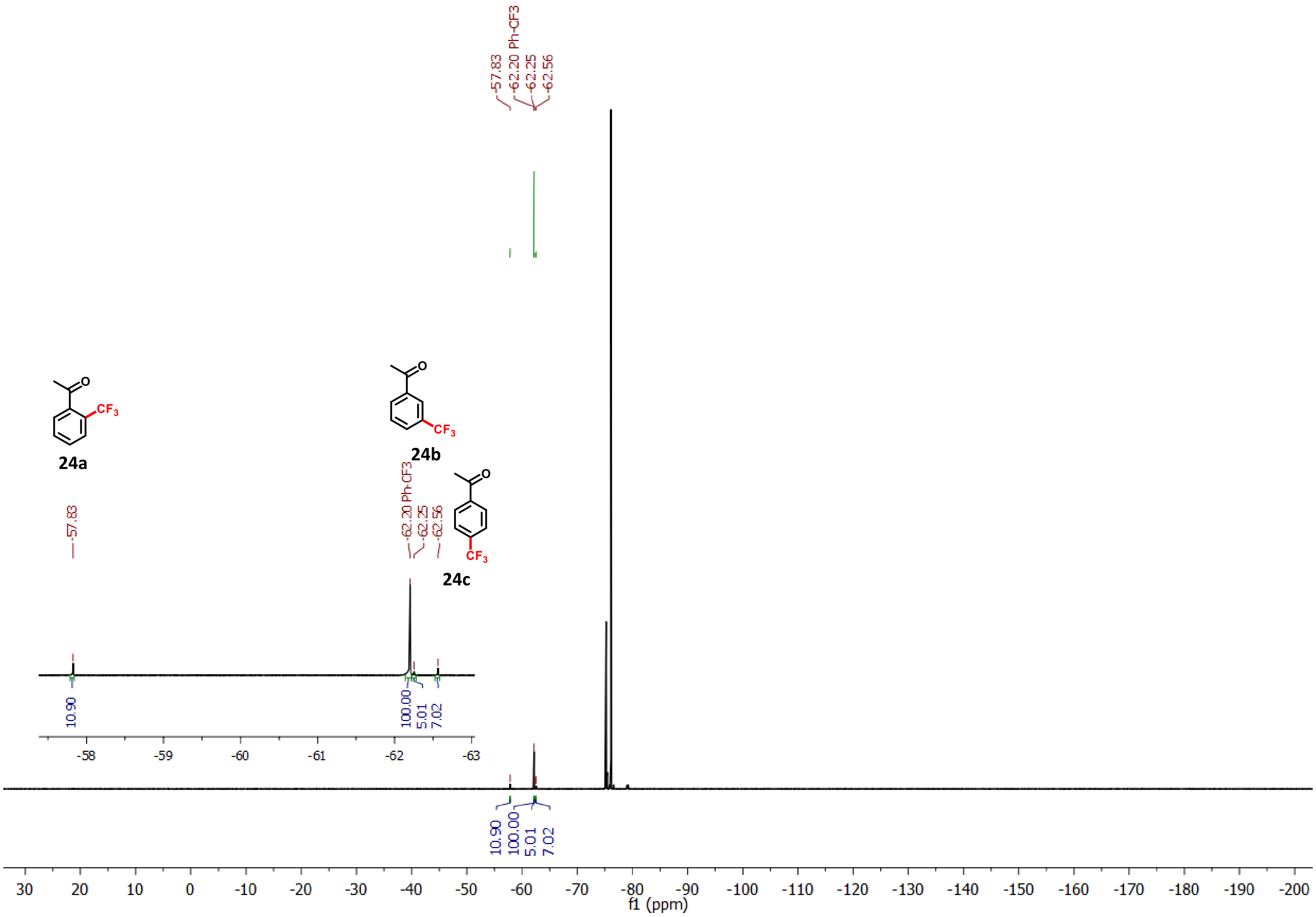

(25) ^{19}F NMR (376 MHz, $CDCl_3$/EtOAc) for trifluoromethylation of methyl benzoate

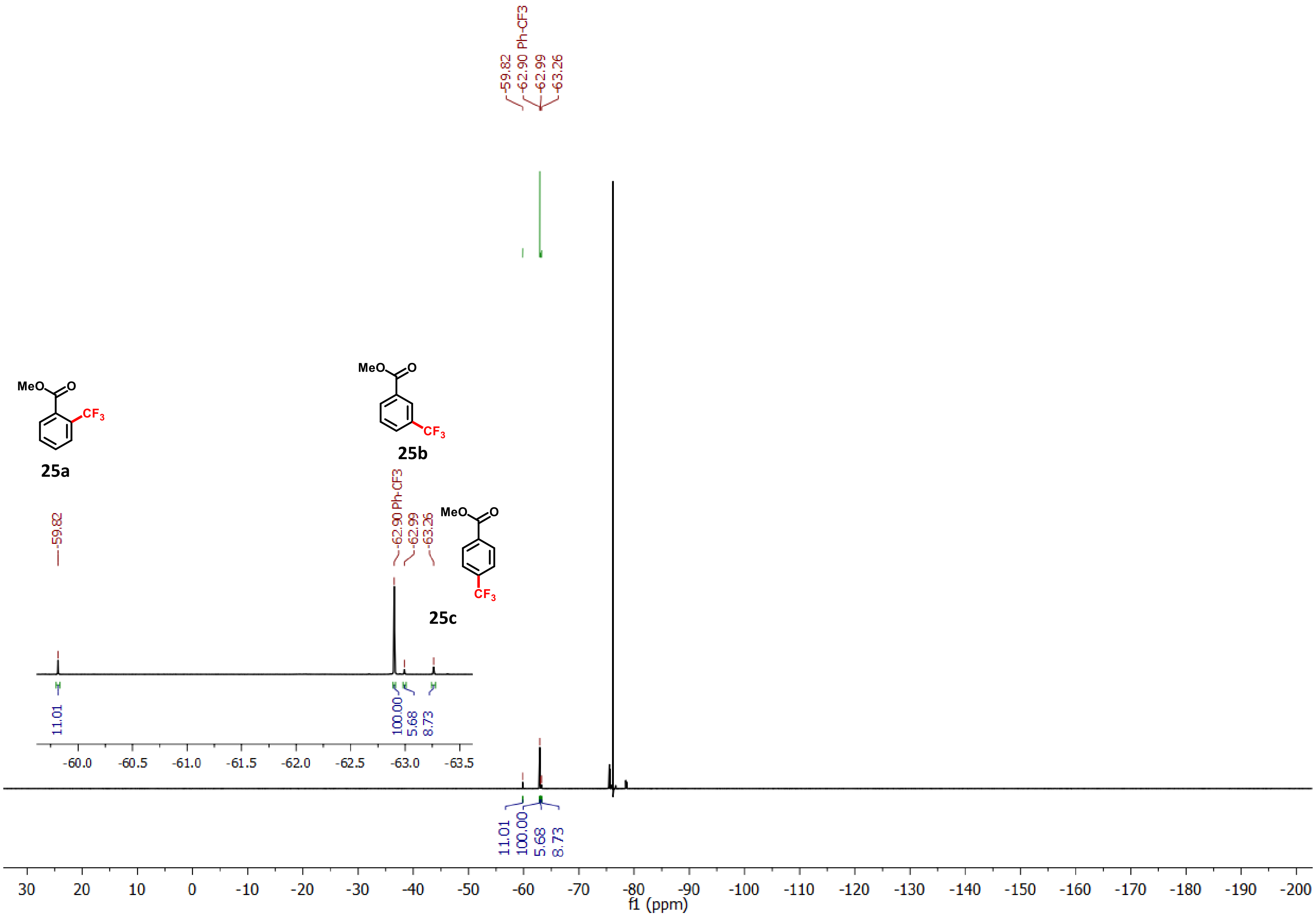

(26) ^{19}F NMR (376 MHz, DMSO-d_6/EtOAc) for trifluoromethylation of 1-(3,4,5-trimethoxyphenyl)ethan-1-one

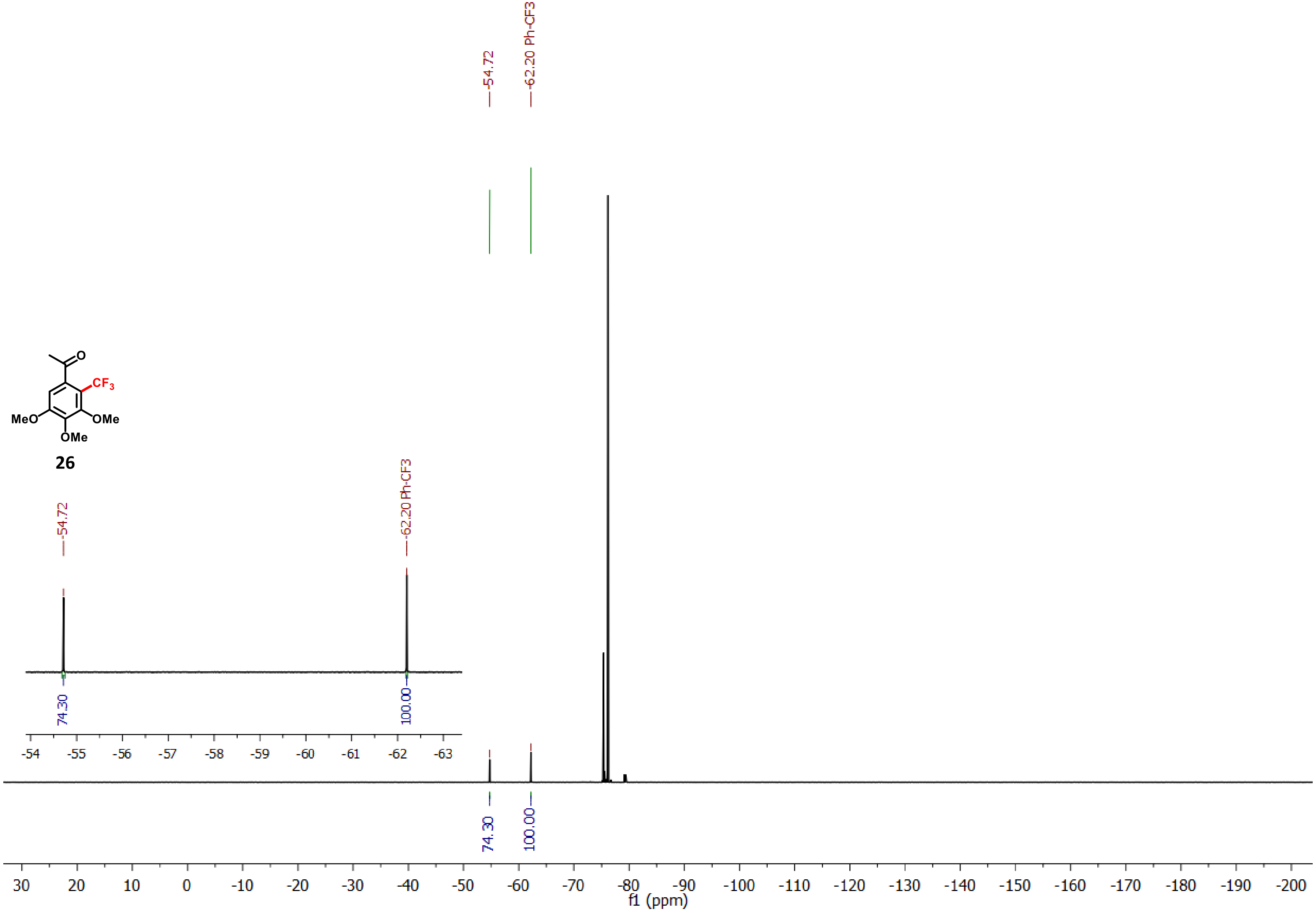

(27) ^{19}F NMR (376 MHz, DMSO-d_6/EtOAc) for trifluoromethylation of 1-methyl-1H-indole

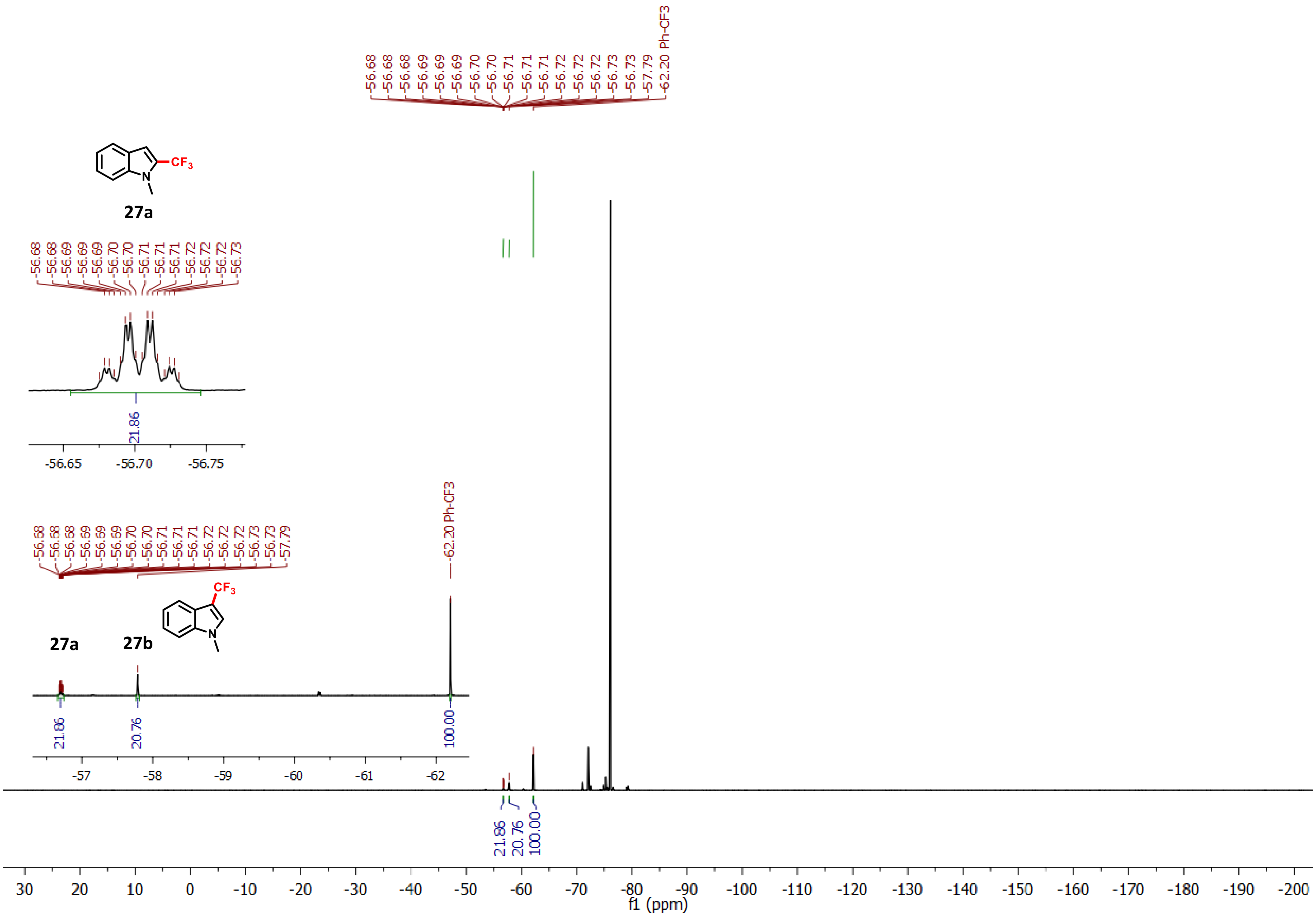

(28) ^{19}F NMR (376 MHz, $CDCl_3$/EtOAc) for trifluoromethylation of tert-butyl 1H-indole-1-carboxylate

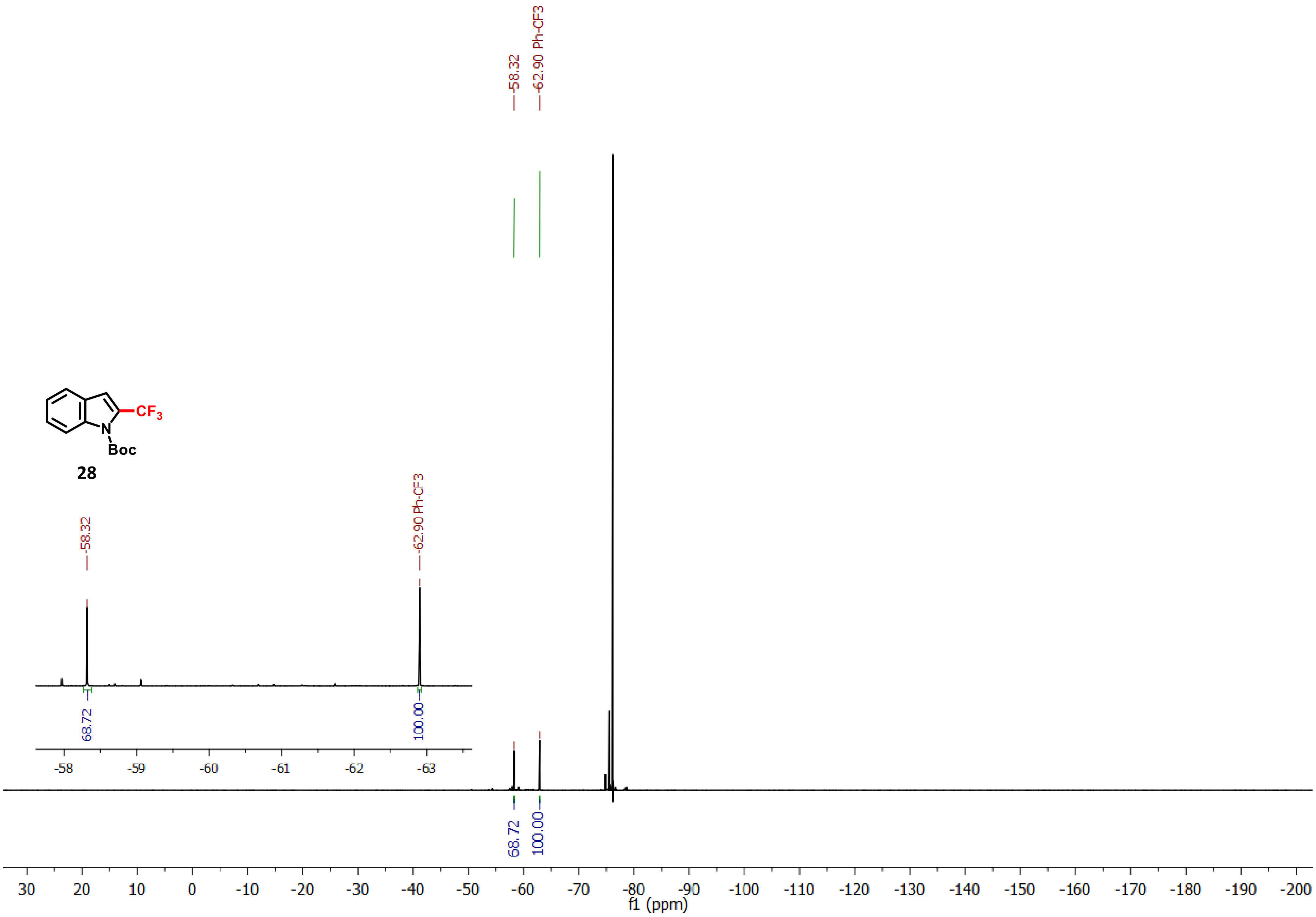

(29) ^{19}F NMR (376 MHz, DMSO-d_6/EtOAc) for trifluoromethylation thiophene

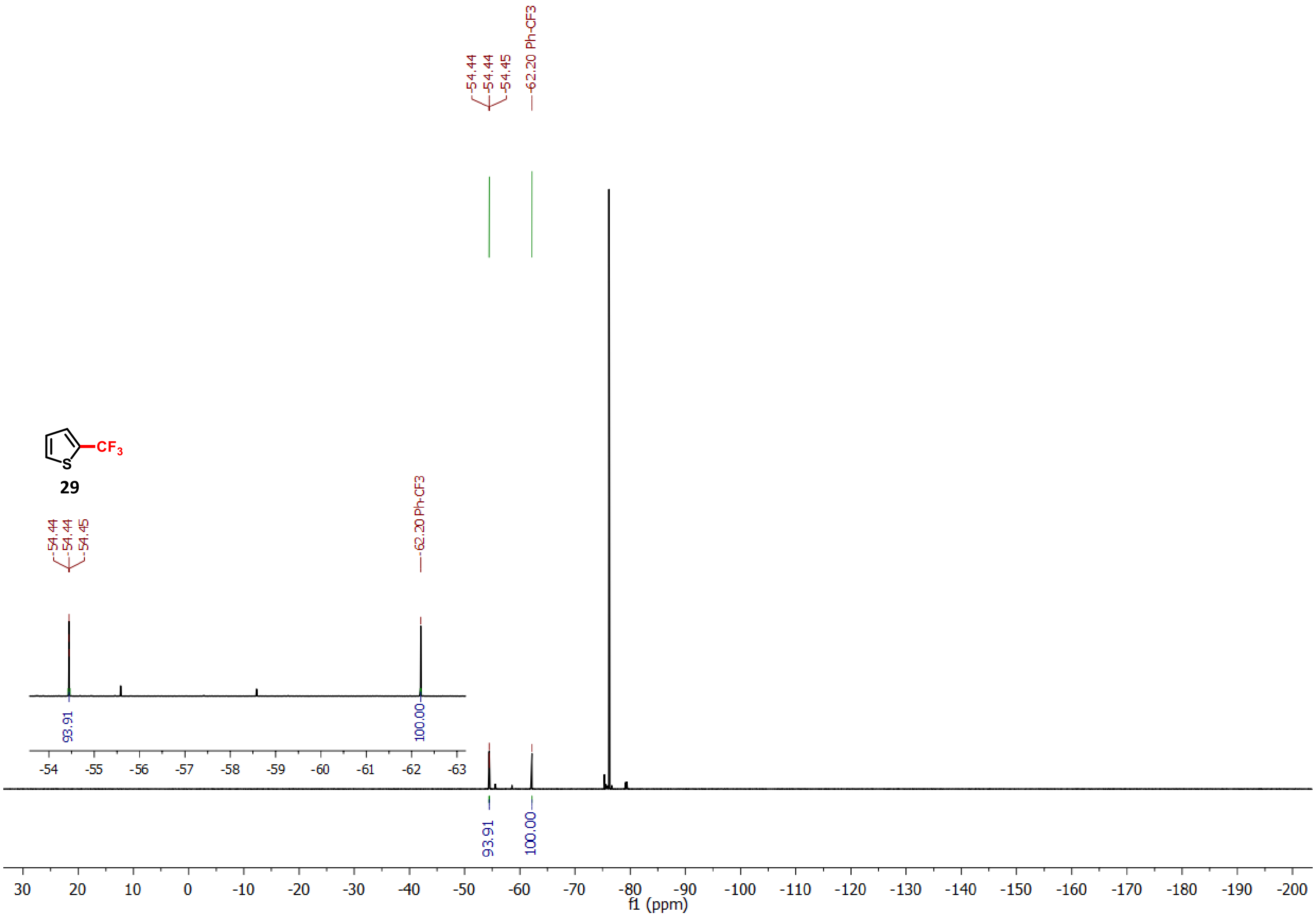

(30) ^{19}F NMR (376 MHz, DMSO-d_6/EtOAc) for trifluoromethylation 2-bromothiophene

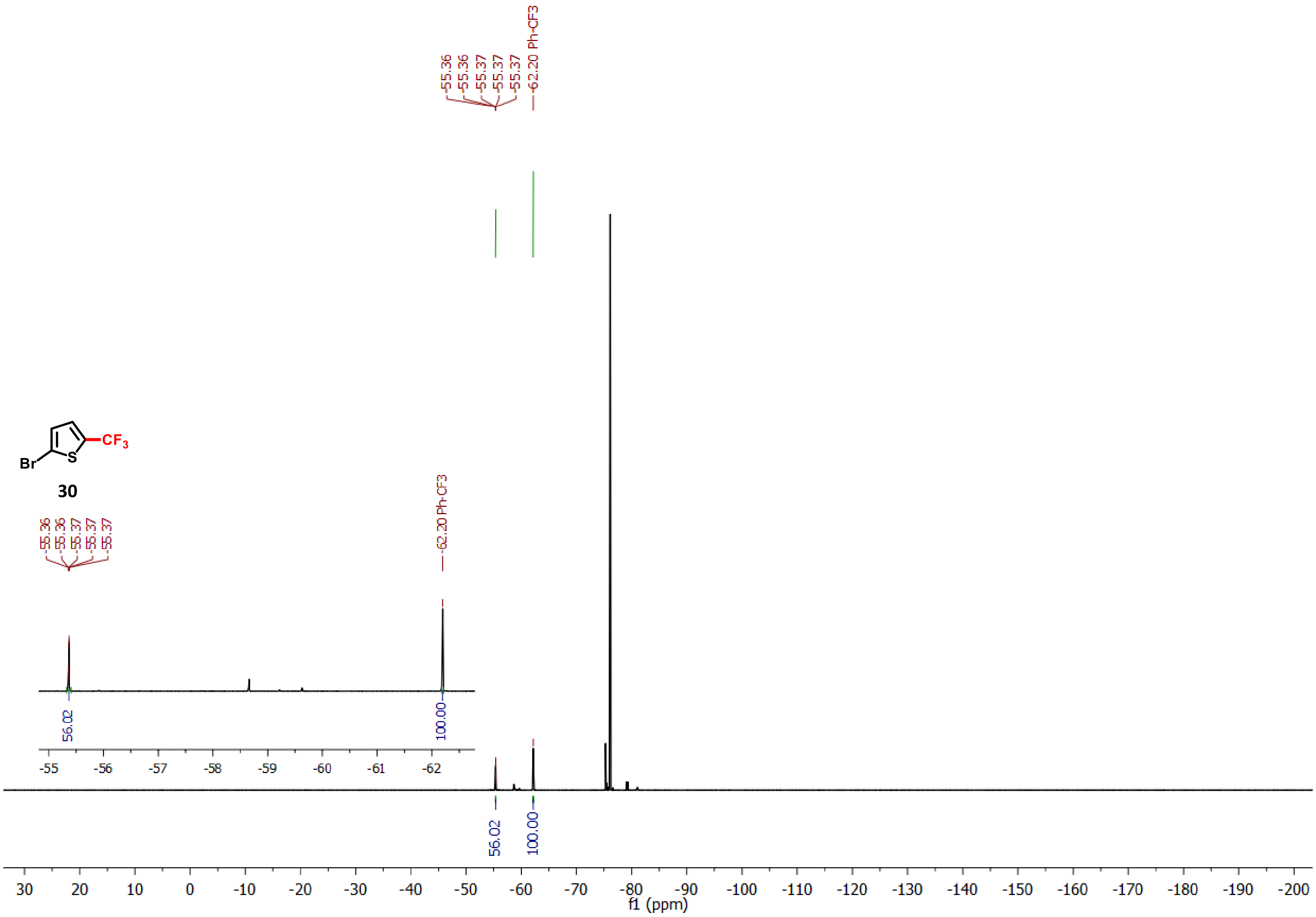

(31) ^{19}F NMR (376 MHz, DMSO-d_6/EtOAc) for trifluoromethylation of caffeine

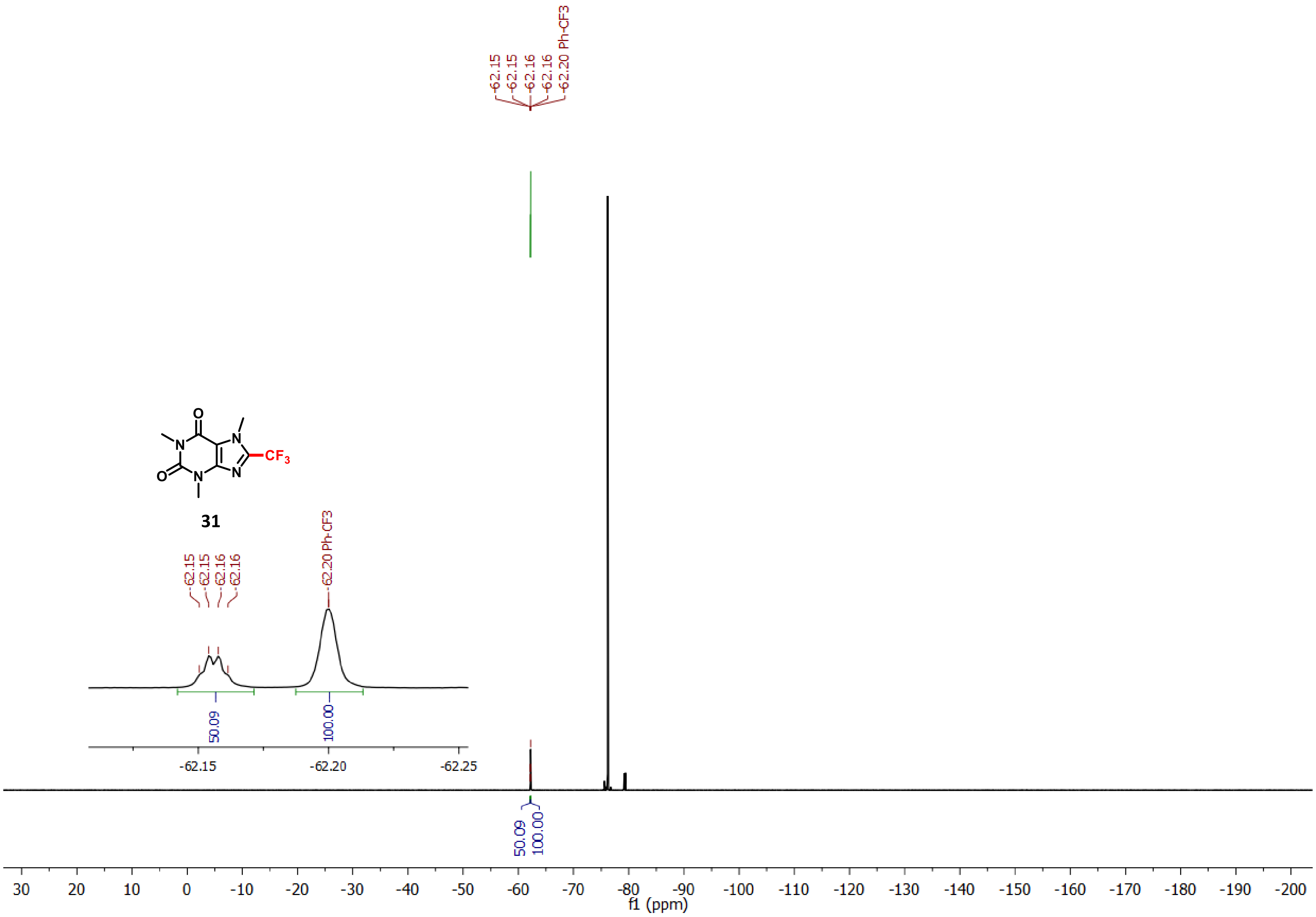

(32) ^{19}F NMR (376 MHz, DMSO-d_6/EtOAc) for trifluoromethylation of chlorobenzene

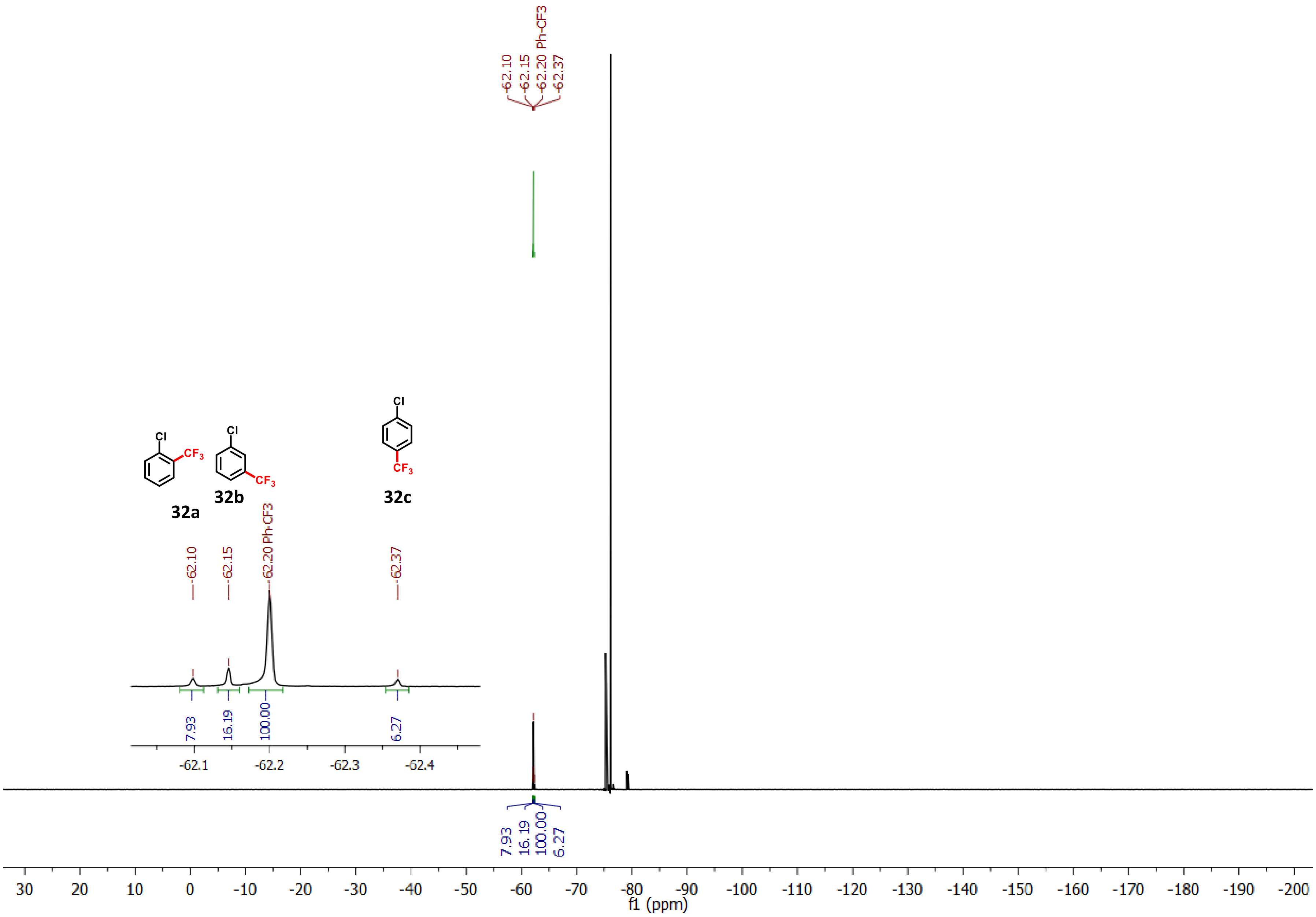

(33) ^{19}F NMR (376 MHz, DMSO-d_6/EtOAc) for trifluoromethylation of bromobenzene

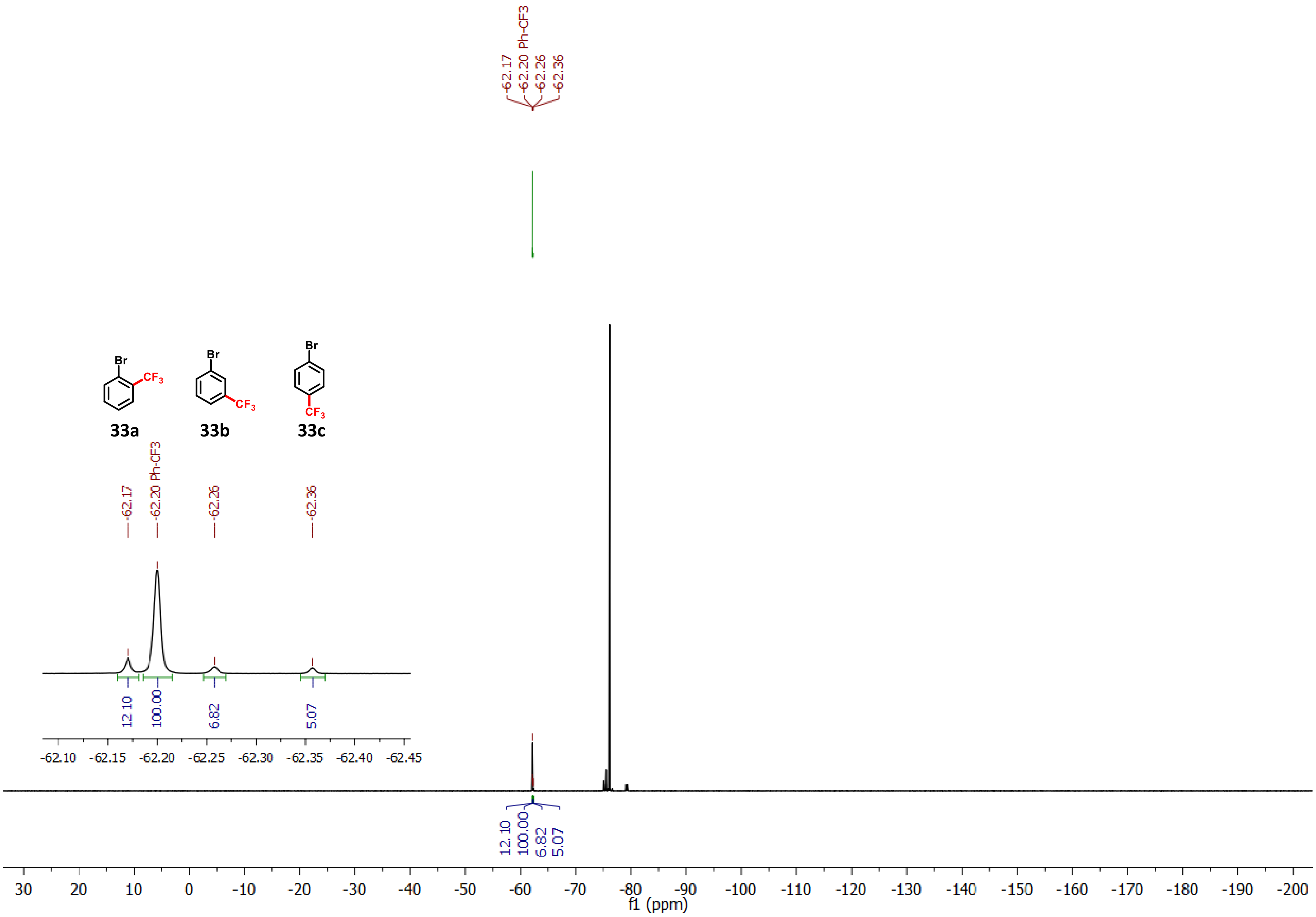

(34) ^{19}F NMR (376 MHz, DMSO-d_6/EtOAc) for trifluoromethylation of iodobenzene

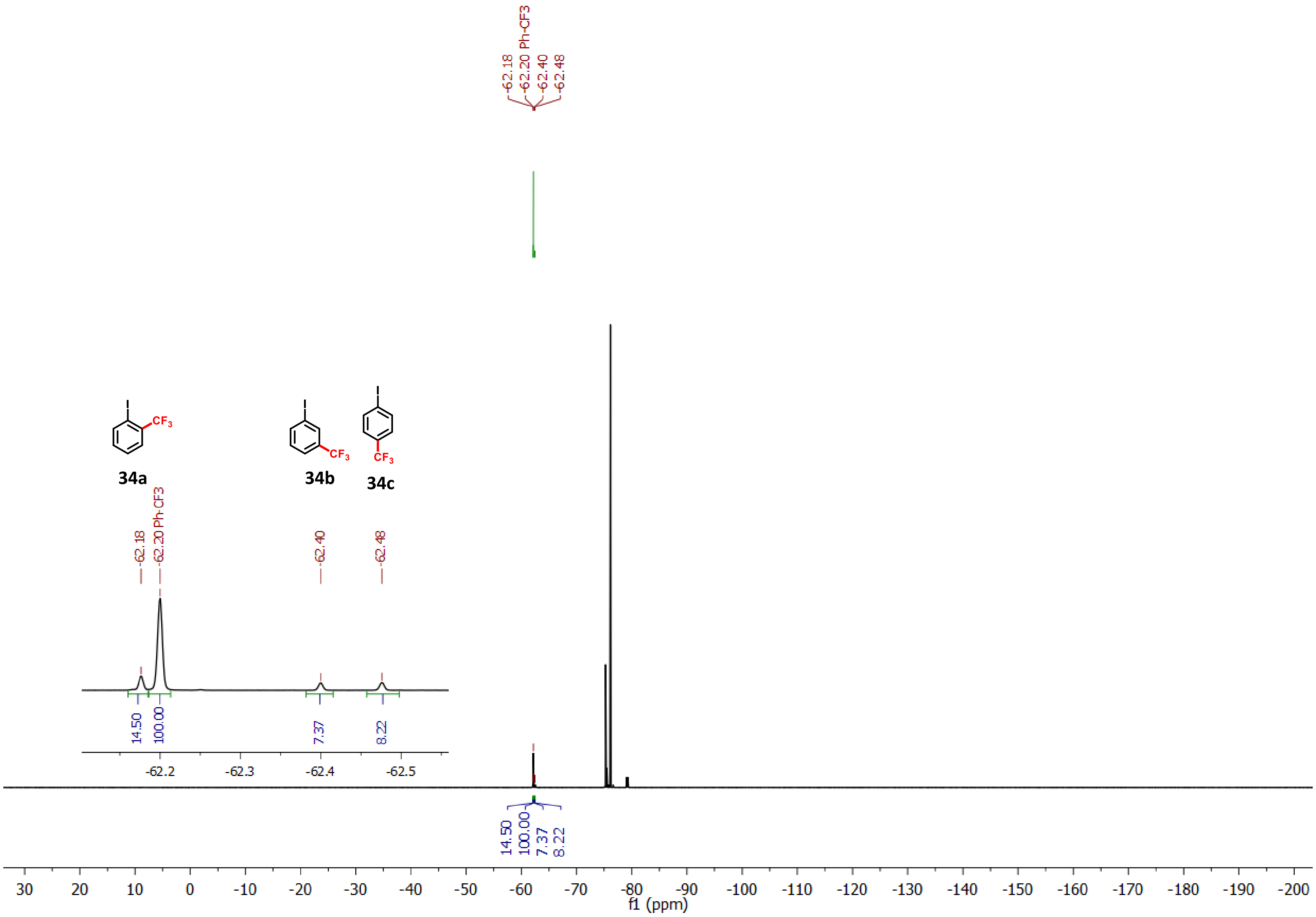

(35) ^{19}F NMR (376 MHz, DMSO-d_6/EtOAc) for trifluoromethylation of 1-Chloro-3-methylbenzene (3-chlorotoluene)

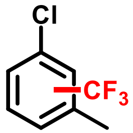

35
The mixture of isomers

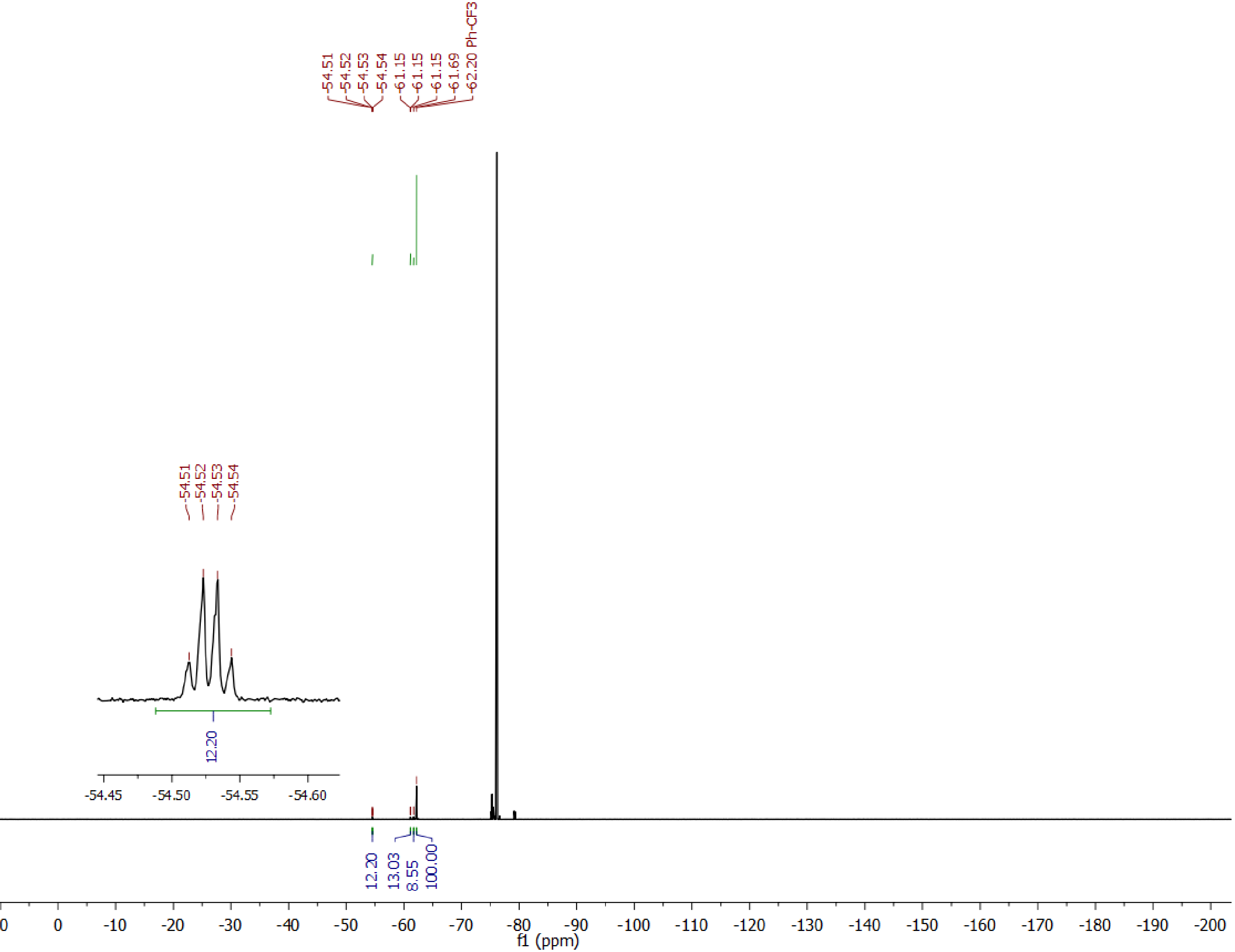

(36) ^{19}F NMR (376 MHz, DMSO-d_6/EtOAc) for trifluoromethylation of 1-bromo-2-methylbenzene (2-bromotoluene)

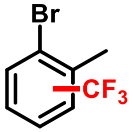

36
The mixture of isomers

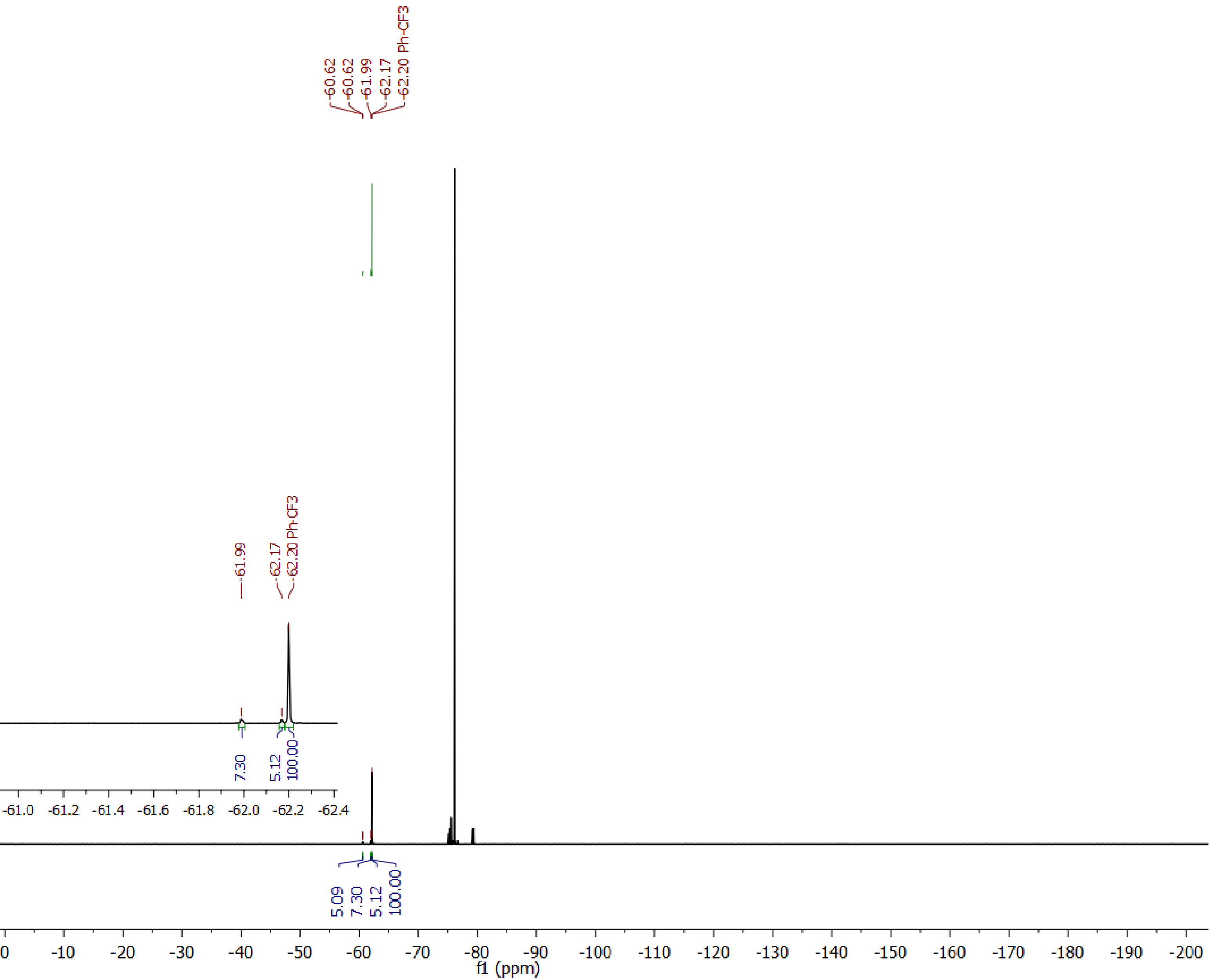

(37) ^{19}F NMR (376 MHz, DMSO-d_6/EtOAc) for trifluoromethylation of 1-bromo-3-methylbenzene (3-bromotoluene)

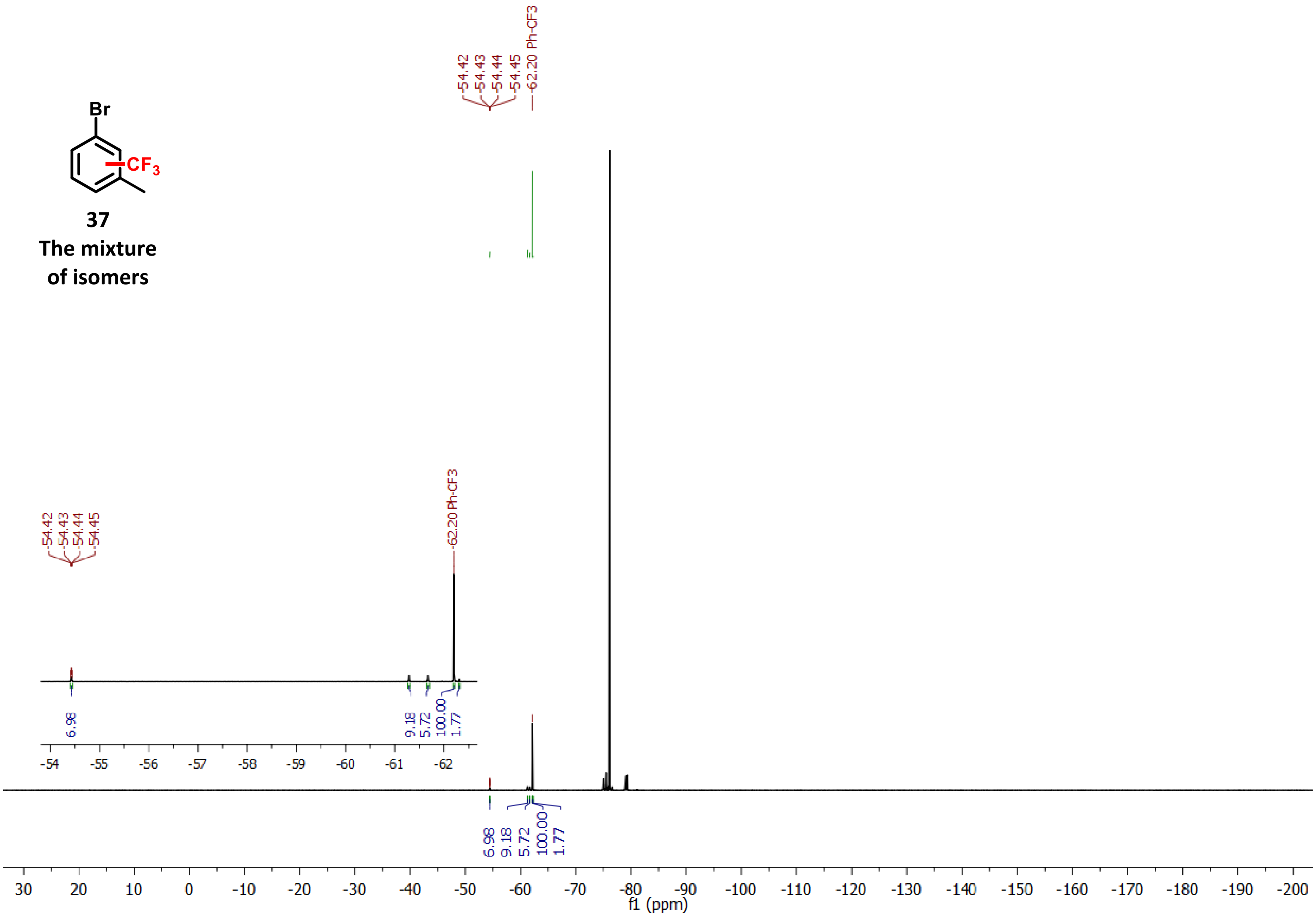

(38) ^{19}F NMR (376 MHz, DMSO-d_6/EtOAc) for trifluoromethylation of 1-bromo-4-methylbenzene (4-bromotoluene)

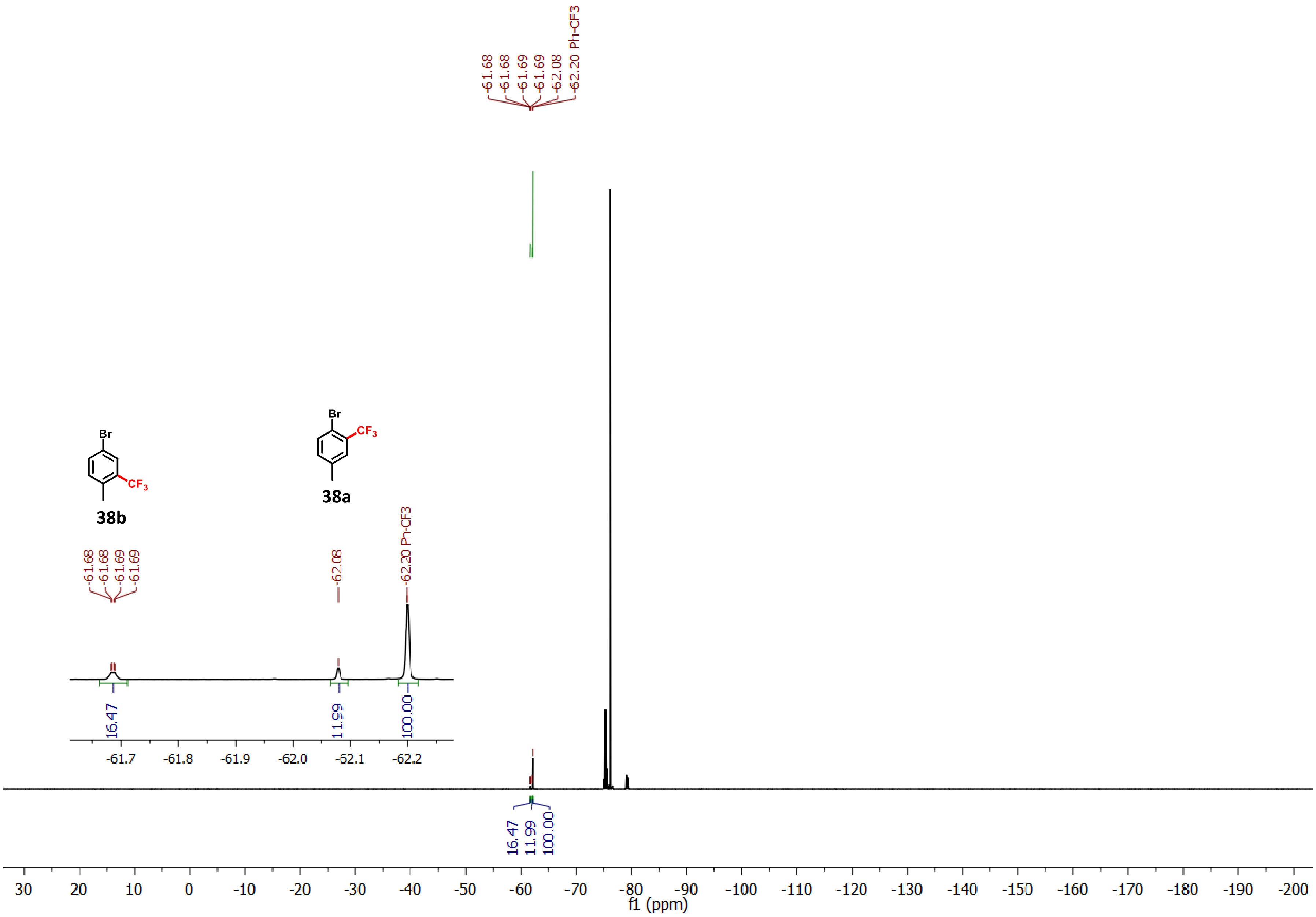

(39) ^{19}F NMR (376 MHz, DMSO-d_6/EtOAc) for trifluoromethylation of 2-bromoanisole

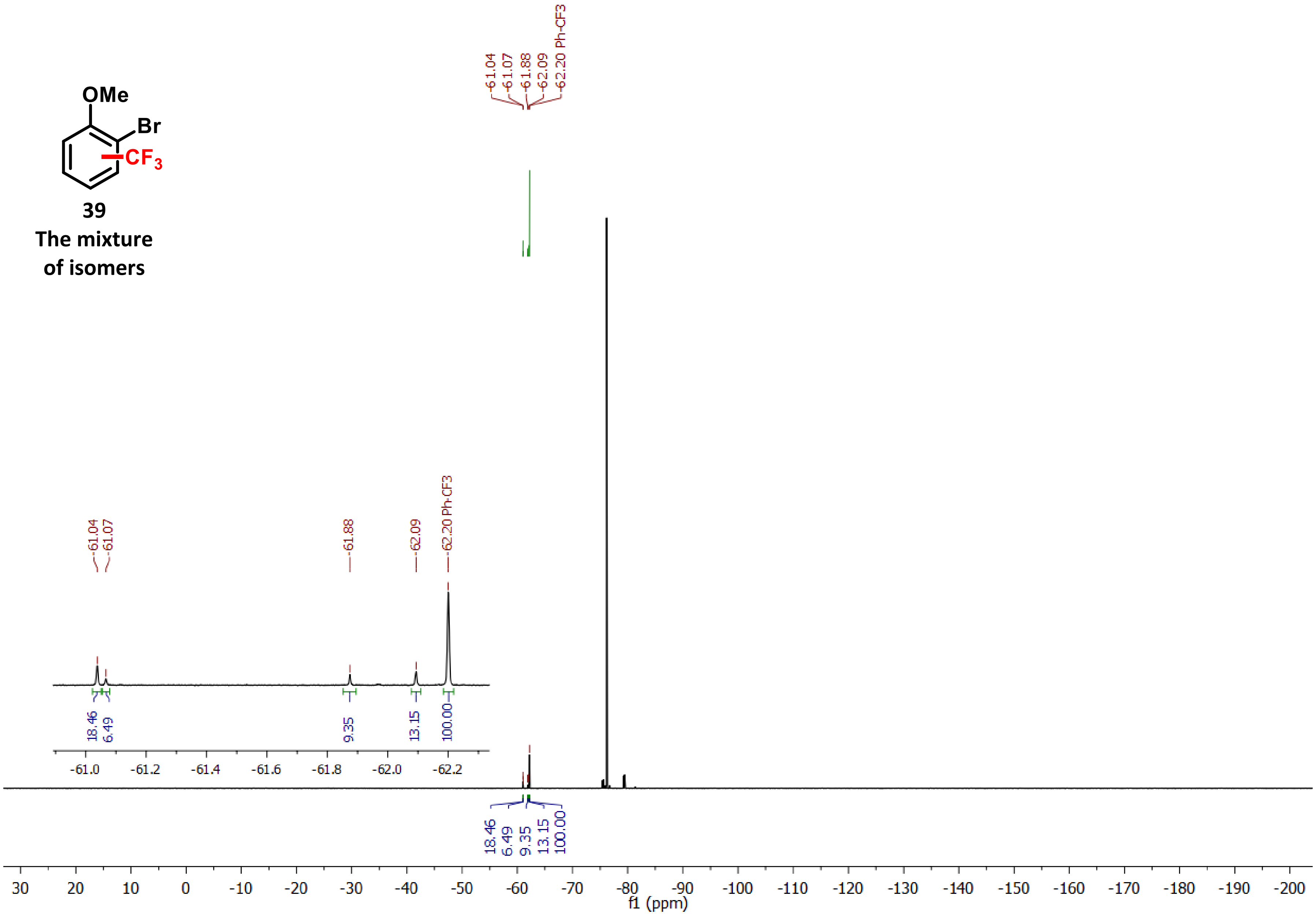

39
The mixture of isomers

(40) ^{19}F NMR (376 MHz, DMSO-d_6/EtOAc) for trifluoromethylation of 3-bromoanisole

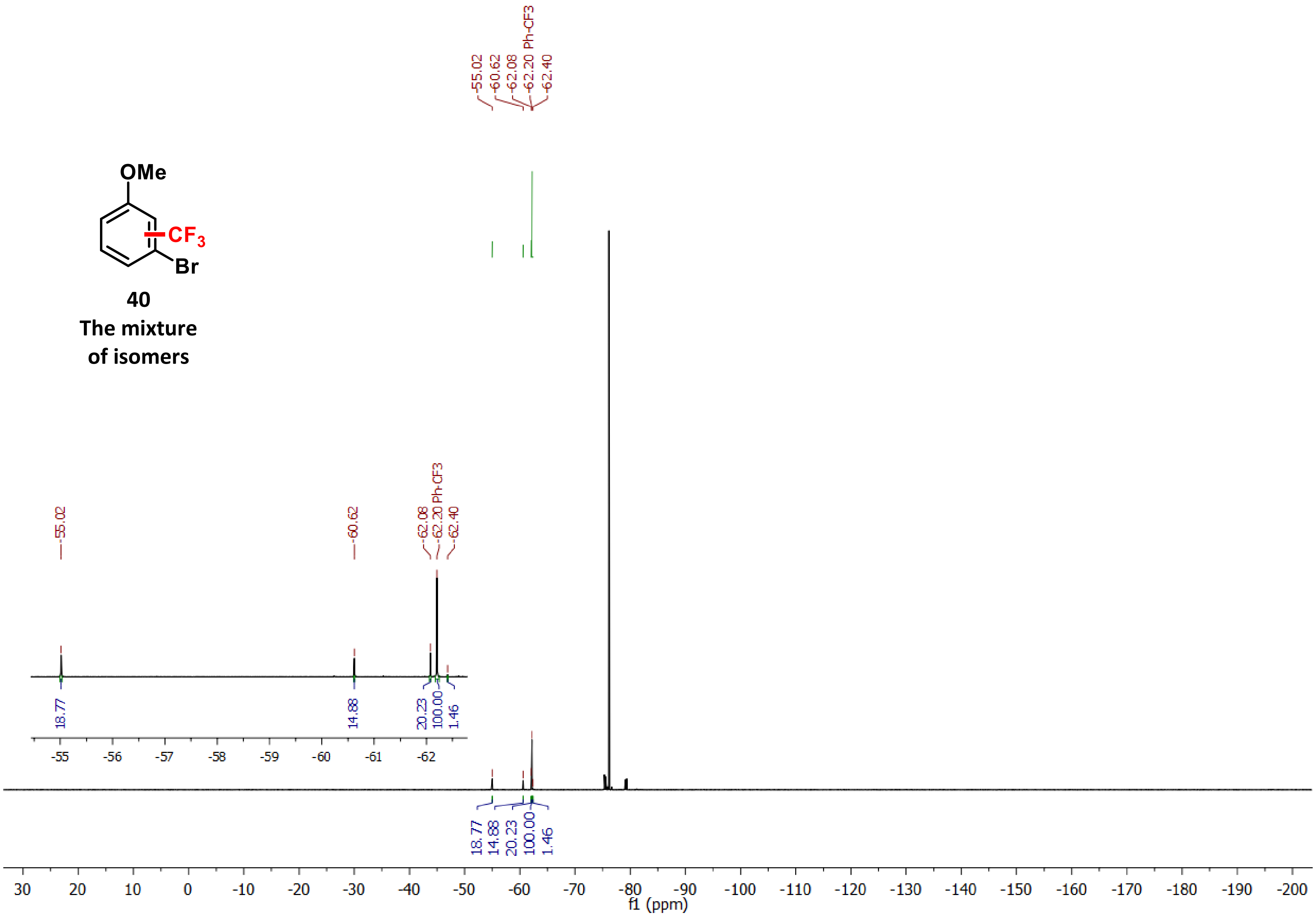

40
The mixture of isomers

(41) ^{19}F NMR (376 MHz, DMSO-d_6/EtOAc) for trifluoromethylation of 4-bromoanisole

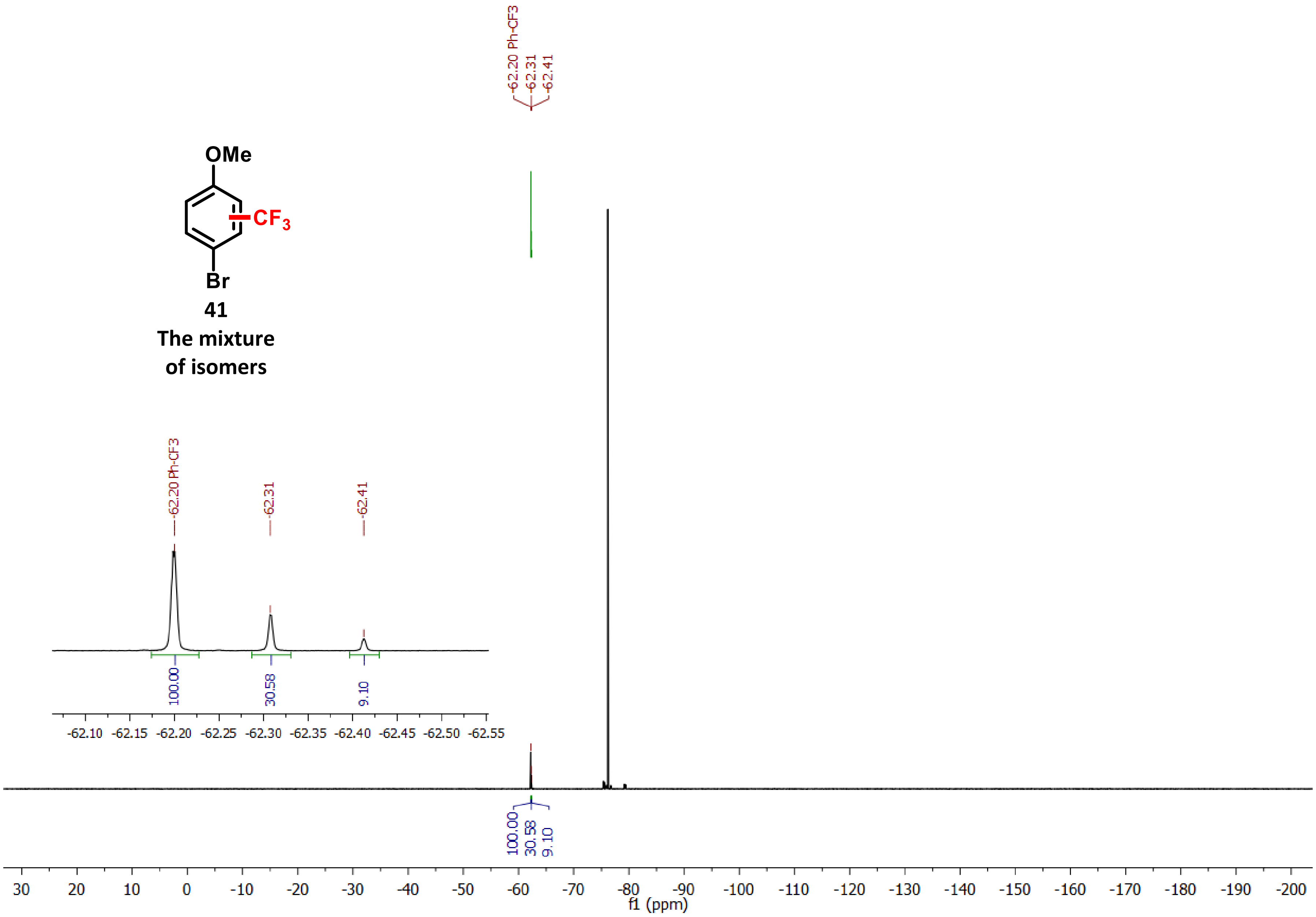

(42) ^{19}F NMR (376 MHz, DMSO-d_6/EtOAc) for trifluoromethylation of 4-bromo-1-methoxy-2-methylbenzene

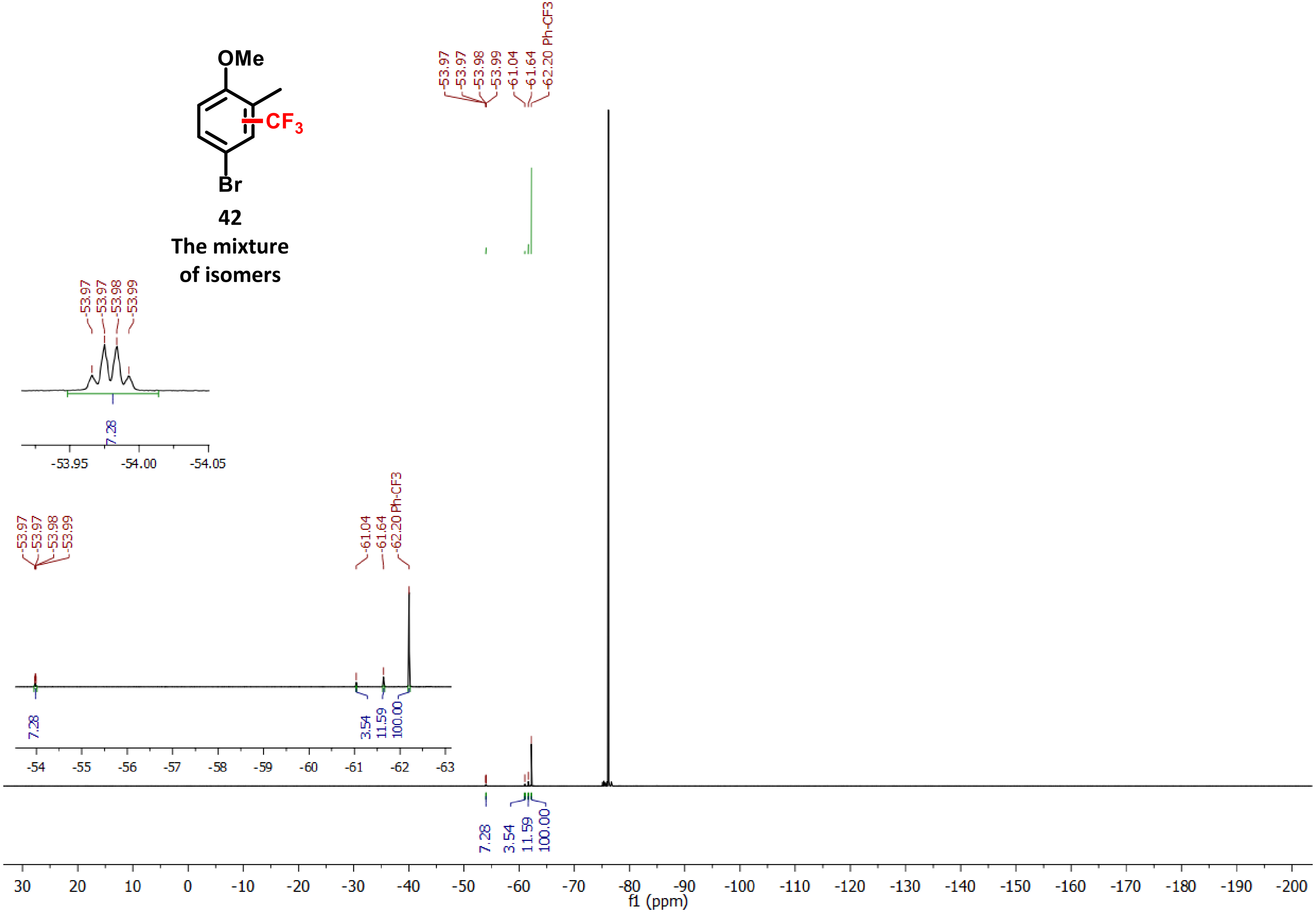

(43) ^{19}F NMR (376 MHz, DMSO-d_6/EtOAc) for trifluoromethylation of 1-bromo-4-methoxy-2-methylbenzene

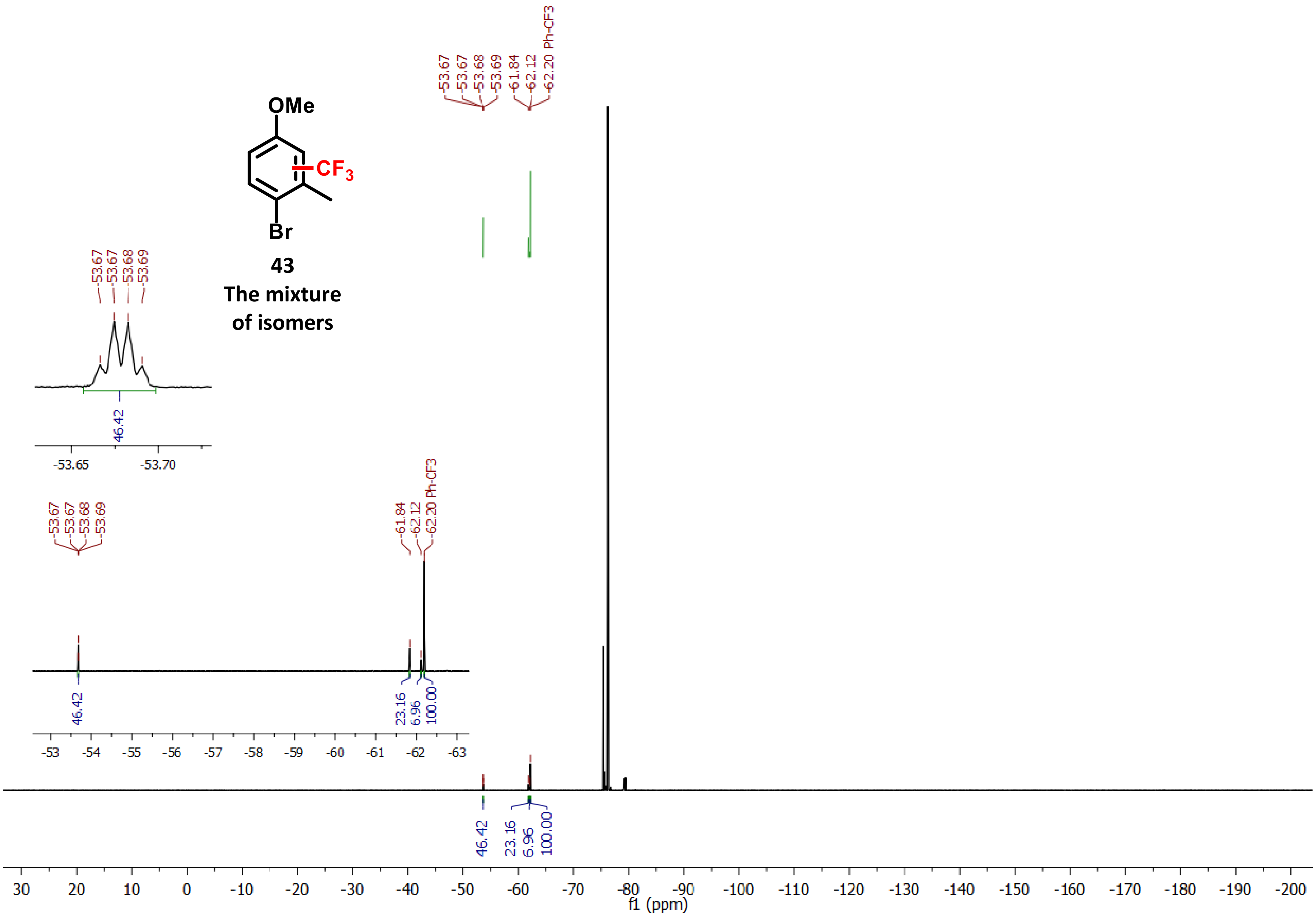

(44) ^{19}F NMR (376 MHz, DMSO-d_6/EtOAc) for trifluoromethylation of 5-Bromo-2-methoxy-1,3-dimethylbenzene

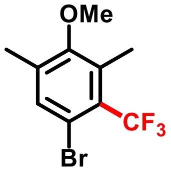

44

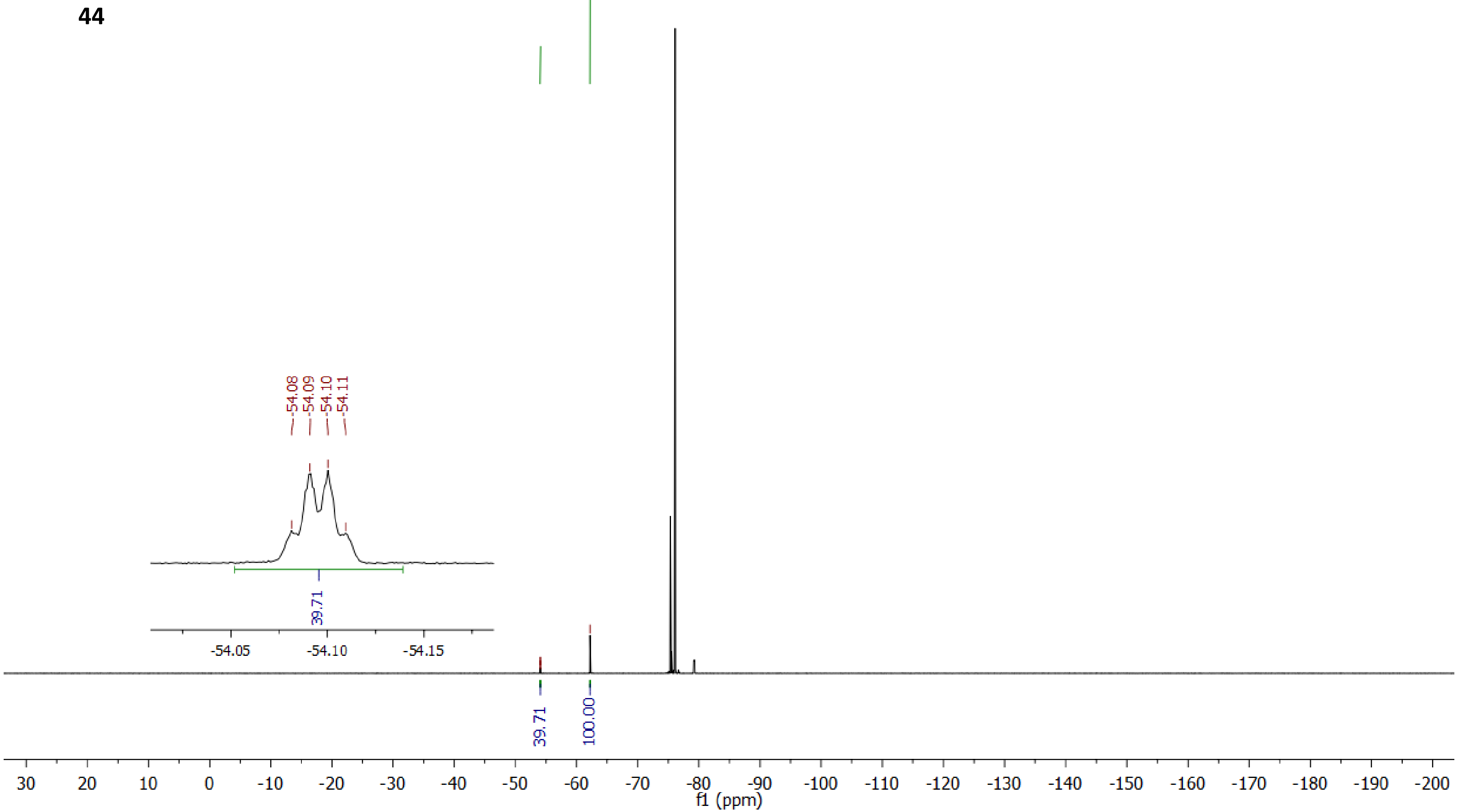

(44) ^{1}H NMR (400 MHz, $CDCl_3$) for 1-bromo-4-methoxy-3,5-dimethyl-2-(trifluoromethyl)benzene

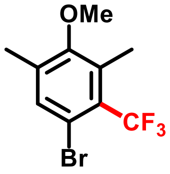

44

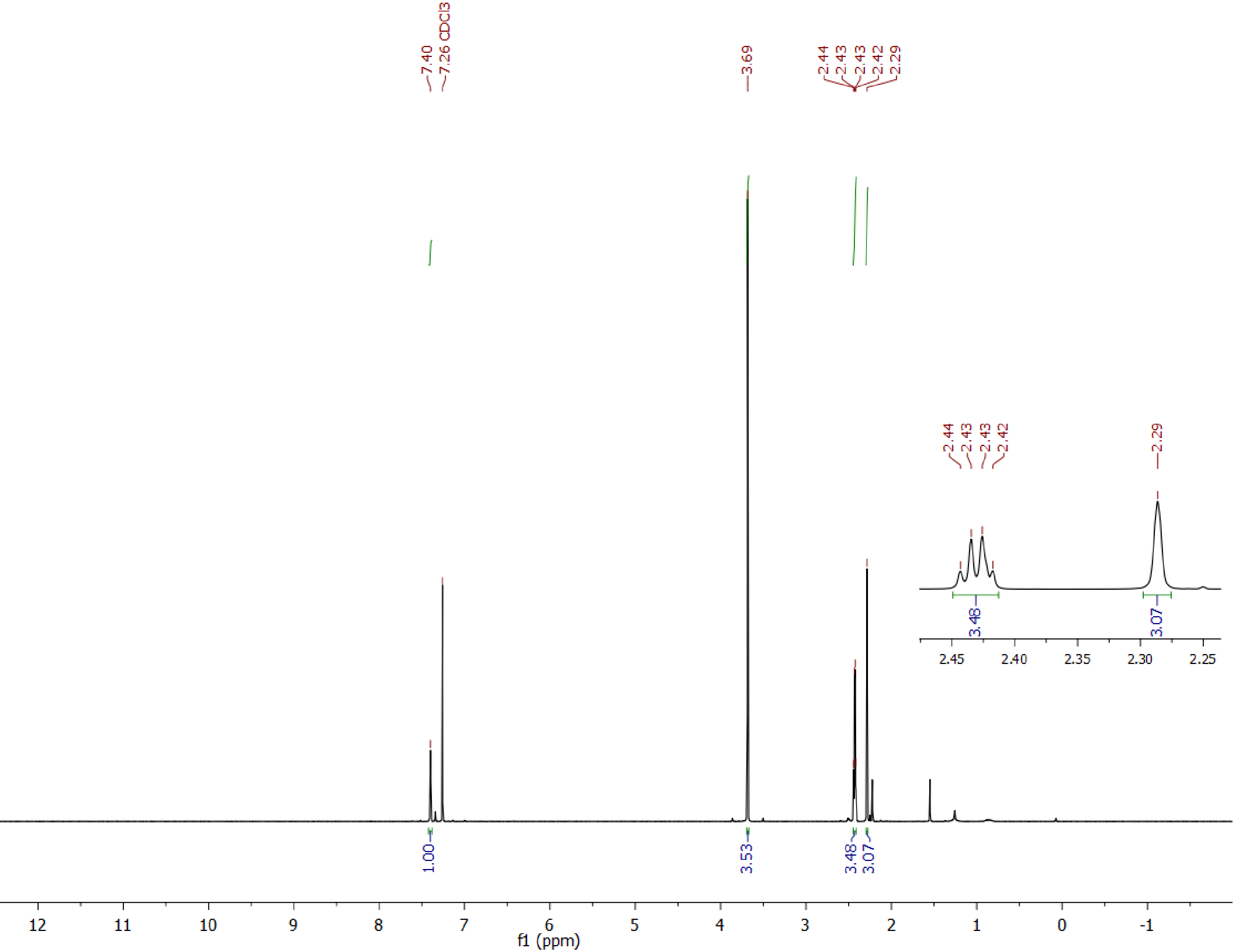

(44) ^{19}F NMR (376 MHz, $CDCl_3$) for 1-bromo-4-methoxy-3,5-dimethyl-2-(trifluoromethyl)benzene

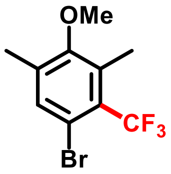

44

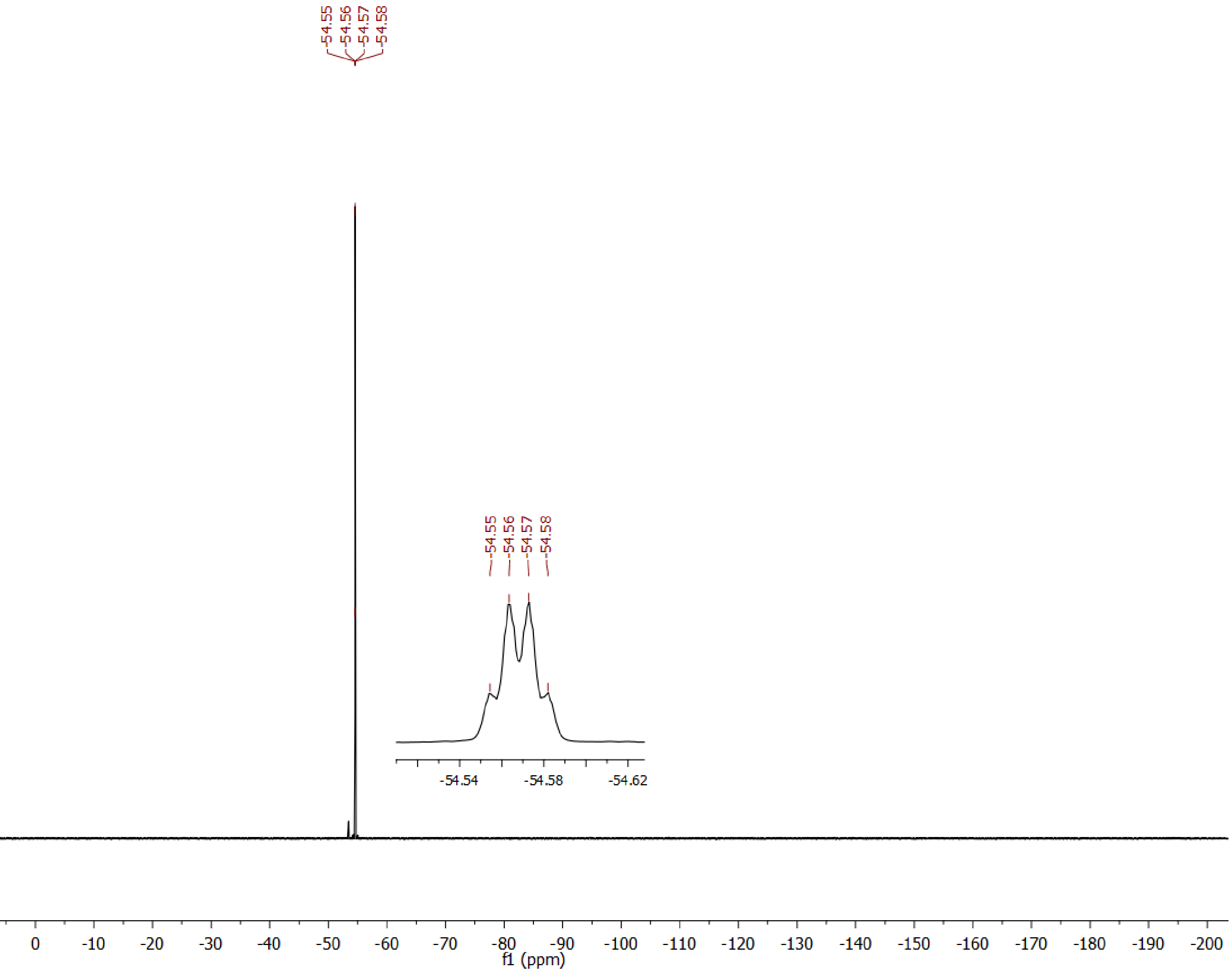

(44) ^{13}C NMR (101 MHz, $CDCl_3$) for 1-bromo-4-methoxy-3,5-dimethyl-2-(trifluoromethyl)benzene

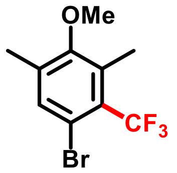

44

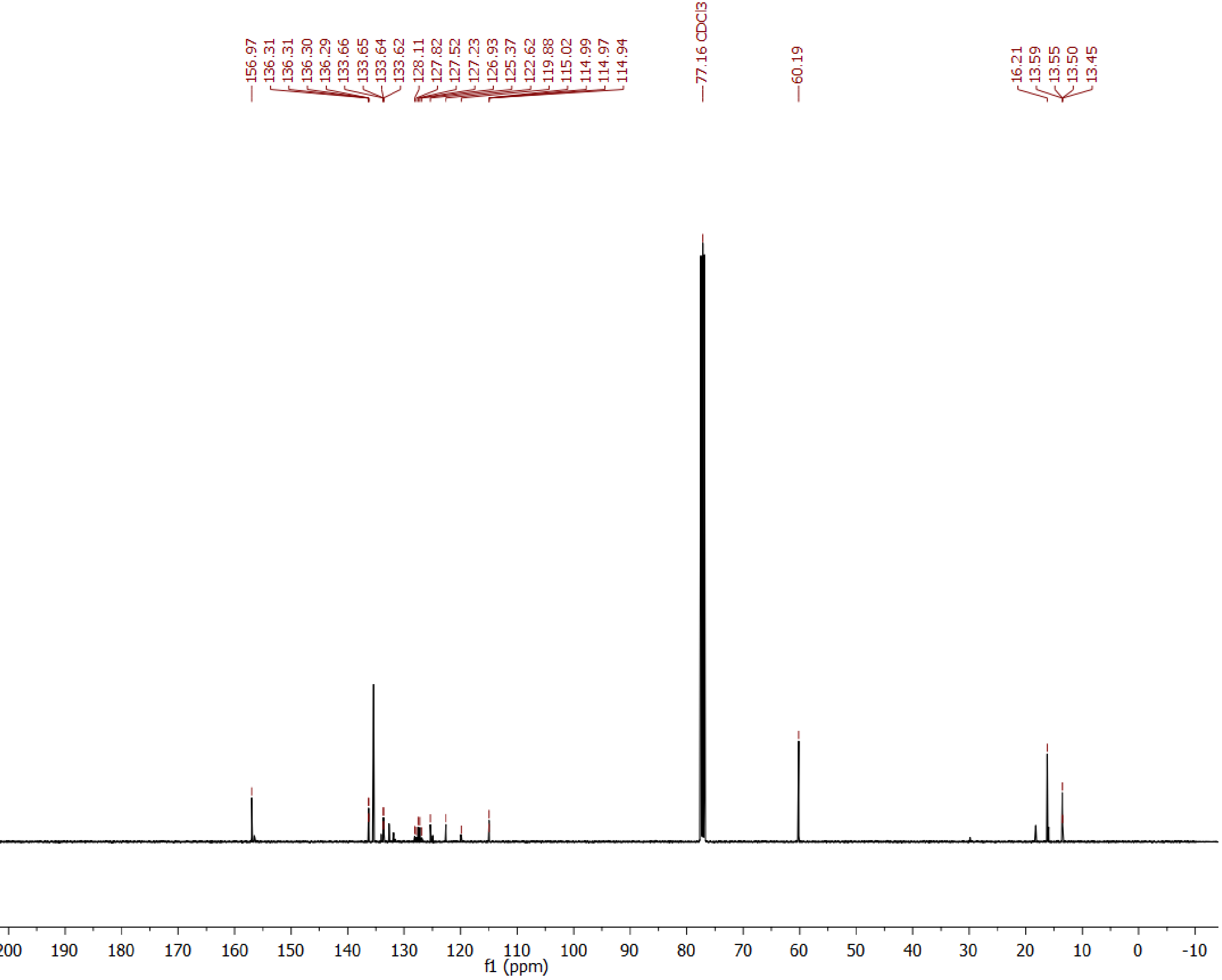

11. Computational data

DFT calculations were carried out with the ORCA 6.0 software package [45–52] using the TPSS0 functional with GRID3 integration and the D4 dispersion correction, together with the def2-SVP basis set for all atoms. Frequency calculations were performed using the PW6B95 functional (with GRID3 integration and D4 dispersion correction) for all structures to confirm whether they correspond to a minimum or a transition state (TS). Transition states were located using the NEB-TS algorithm implemented in ORCA 6.0. All structures and energies were optimized and calculated with the SMD solvent model for EtOAc to account for solvent effects. Gibbs free energy values are reported at 298 K, unless stated otherwise. TDDFT calculations were carried out to optimize the geometry of the T1 state for PS at the TPSS0/def2-SVP level of theory (with GRID3 integration and D4 dispersion correction).

PS (3DPAFIPN)

Charge: 0

Multiplicity: 1

Number of Imaginary Frequencies: 0

Final single point energy -2069.682594077043 Eh

C -0.86934 1.35052 -0.82751

C -1.57788 0.12790 -0.80755

C -1.06662 -0.93376 -1.55272

C 0.10659 -0.84378 -2.30064

C 0.80662 0.38394 -2.28621

C 0.32203 1.48131 -1.55770

N 1.02226 2.69779 -1.55867

F -1.72892 -2.08522 -1.55009

N -2.74866 -0.02886 -0.07049

N 0.55845 -1.93703 -3.03476

C 2.33546 2.72436 -1.03852

C 0.38779 3.84532 -2.08482

C -3.89524 -0.56989 -0.69570

C -2.74647 0.31770 1.30305

C 0.85838 -1.76630 -4.40861

C 0.66626 -3.19899 -2.40660

C -4.74906 -1.43110 0.00167

C -5.86910 -1.95695 -0.63289

C -6.14038 -1.65076 -1.96654

C -5.28178 -0.80059 -2.66091

C -4.16968 -0.25190 -2.02988

C -3.84882 0.95924 1.87599

C -3.81897 1.31225 3.21998

C -2.69065 1.05025 3.99801

C -1.59216 0.41631 3.42163

C -1.62036 0.03773 2.08256

C 1.08717 -3.27498 -1.07496

C 1.17207 -4.51138 -0.44225

C 0.86056 -5.68118 -1.13263

C 0.45107 -5.60188 -2.46380

C 0.34244 -4.37011 -3.09980

C 1.96627 -2.40145 -4.97789

C 2.25829 -2.20426 -6.32239

C 1.46687 -1.36252 -7.10483

C 0.36730 -0.72708 -6.53214

C 0.05253 -0.93548 -5.19243

C -0.48395 3.71651 -3.17163

C -1.14531 4.83513 -3.67046

C -0.93142 6.09182 -3.10880

C -0.05472 6.21804 -2.03066

C 0.59469 5.10476 -1.51040

C 3.31810 3.53718 -1.61527

C 4.60985 3.53151 -1.10224

C 4.94617 2.70585 -0.02891

C 3.96913 1.88882 0.53536

C 2.66681 1.90228 0.04416

C 2.01996 0.52226 -3.01889

N 3.00810 0.64561 -3.60881

C -1.35821 2.47114 -0.09718

N -1.74799 3.39025 0.48843

H -4.53159 -1.69078 1.03520

H -6.52825 -2.62602 -0.08164

H -7.01490 -2.07142 -2.45971

H -5.48430 -0.54760 -3.70057

H -3.51377 0.42917 -2.56943

H -4.71802 1.19127 1.26472

H -4.67954 1.81500 3.65867

H -2.66911 1.33982 5.04712

H -0.70841 0.19998 4.01992

H -0.76893 -0.47404 1.63732

H 1.35313 -2.36543 -0.53891

H 1.49919 -4.55851 0.59538

H 0.93664 -6.64799 -0.63820

H 0.19750 -6.50819 -3.01183

H 0.00236 -4.31313 -4.13132

H 2.60156 -3.03525 -4.36332

H 3.12493 -2.69914 -6.75804

H 1.70772 -1.20331 -8.15441

H -0.26226 -0.07317 -7.13361

H -0.81760 -0.45338 -4.75031

H -0.64067 2.74050 -3.62715

H -1.82330 4.72099 -4.51505

H -1.44427 6.96635 -3.50527

H 0.11280 7.19281 -1.57476

H 1.25430 5.20686 -0.65183

H 3.07083 4.16188 -2.47037

H 5.36667 4.16688 -1.56005

H 5.96218 2.69900 0.36177

H 4.21491 1.24204 1.37637

H 1.90367 1.27541 0.50155

PS* (3DPAFIPN*)

Charge: 0

Multiplicity: 3

Number of Imaginary Frequencies: 0

Final single point energy -2069.682594077043 Eh

C 1.21220 0.71223 -0.34089

C 1.14002 -0.73671 -0.39459

C -0.02770 -1.39618 -0.00709

C -1.17718 -0.64124 0.40001

C -1.16607 0.80751 0.29750

C 0.01998 1.42043 -0.04807

C 2.21776 -1.45601 -0.96714

C -2.27784 -1.26874 1.01207

N 3.11617 -2.01270 -1.45153

N -3.20751 -1.75532 1.52173

N 2.42099 1.38978 -0.52085

N -2.33571 1.55444 0.53464

N -0.09543 -2.79337 0.00714

F 0.06480 2.74516 -0.07374

C 2.50958 2.40023 -1.49389

C 3.31250 3.53539 -1.27980

C 1.75529 2.29328 -2.67543

C 3.36243 4.53447 -2.24202

H 3.86972 3.63894 -0.35169

C 1.81399 3.30233 -3.62711

H 1.14607 1.40748 -2.84300

C 2.61689 4.42600 -3.41851

H 3.97730 5.41546 -2.06517

H 1.23715 3.20593 -4.54543

H 2.65928 5.21495 -4.16734

C 3.53270 1.02961 0.26820

C 4.82859 1.01962 -0.27650

C 3.33212 0.62975 1.59970

C 5.90310 0.63182 0.51186

H 4.98100 1.29025 -1.31874

C 4.41689 0.23949 2.37488

H 2.32895 0.65392 2.01989

C 5.70546 0.23989 1.83840

H 6.90321 0.61740 0.08181

H 4.25579 -0.05693 3.41008

H 6.55237 -0.06797 2.44912

C -3.43769 1.36370 -0.33571

C -4.75132 1.33915 0.15528

C -3.21614 1.14677 -1.70204

C -5.81737 1.12660 -0.71290

H -4.93420 1.47785 1.21868

C -4.28927 0.91729 -2.55990

H -2.19888 1.16441 -2.08912

C -5.59572 0.91205 -2.07405

H -6.83161 1.11060 -0.31574

H -4.09897 0.75287 -3.61989

H -6.43343 0.73762 -2.74732

C -2.36769 2.47059 1.60495

C -1.58709 2.24034 2.74882

C -3.14150 3.64064 1.53842

C -1.58505 3.15631 3.79639

H -0.98552 1.33579 2.81562

C -3.13978 4.54298 2.59743

H -3.73642 3.84827 0.65187

C -2.36325 4.31191 3.73328

H -0.97456 2.95552 4.67608

H -3.74464 5.44620 2.52434

H -2.36277 5.02445 4.55644

C -1.16342 -3.43835 -0.65926

C -1.72529 -4.61420 -0.14037

C -1.67770 -2.88673 -1.84131

C -2.78387 -5.22400 -0.80161

H -1.34274 -5.03497 0.78666

C -2.74971 -3.49820 -2.48488

H -1.22711 -1.98850 -2.25872

C -3.30523 -4.66923 -1.97284

H -3.21838 -6.13278 -0.38788

H -3.14264 -3.06023 -3.40114

H -4.13970 -5.14883 -2.48161

C 0.91409 -3.54901 0.65777

C 1.40366 -3.12566 1.90023

C 1.42182 -4.71559 0.07175

C 2.39537 -3.86028 2.54218

H 0.99436 -2.23067 2.36436

C 2.40207 -5.45066 0.73028

H 1.05759 -5.03544 -0.90206

C 2.89639 -5.02689 1.96410

H 2.76706 -3.52535 3.50945

H 2.79401 -6.35411 0.26548

H 3.66795 -5.60282 2.47239

TFAA

Charge: 0

Multiplicity: 1

Number of Imaginary Frequencies: 0

Final single point energy -977.491910351521 Eh

C -6.93403 -0.11859 0.24107

C -5.80327 0.81843 -0.21637

F -6.80212 -0.39126 1.53409

F -6.88661 -1.25898 -0.43730

F -8.10191 0.45797 0.03692

O -5.95762 1.88827 -0.69134

O -4.61717 0.17925 -0.00205

C -3.41319 0.81907 -0.04853

O -3.22802 1.98505 -0.03531

C -2.30947 -0.25171 -0.08401

F -2.46059 -1.02357 -1.15383

F -2.37612 -1.01482 1.00070

F -1.12633 0.32789 -0.13207

TFAA$^{\bullet-}$

Charge: -1

Multiplicity: 2

Number of Imaginary Frequencies: 0

Final single point energy -977.578110825612 Eh

C -6.91891 -0.12530 0.15329

C -5.60825 0.68553 0.19808

F -6.72320 -1.38080 0.56980

F -7.40952 -0.18698 -1.08826

F -7.85094 0.41730 0.93031

O -5.52422 1.67113 0.88285

O -4.73727 0.12885 -0.58153

C -3.34313 0.81400 -0.76576

O -3.01677 0.99224 -1.94635

C -2.46141 0.04341 0.17232

F -2.18110 -1.23082 -0.21269

F -2.97297 -0.06259 1.41092

F -1.26878 0.65106 0.28897

PS$^{\bullet+}$ (3DPAFIPN$^{\bullet+}$)

Charge: +1

Multiplicity: 2

Number of Imaginary Frequencies: 0

Final single point energy -2069.471885850890 Eh

C -0.88105 1.34308 -0.84631

C -1.62858 0.14625 -0.86533

C -1.09657 -0.93679 -1.58666

C 0.10089 -0.83601 -2.27374

C 0.83366 0.36699 -2.25845

C 0.35232 1.46594 -1.52689

N 1.07329 2.65580 -1.48486

F -1.74659 -2.08746 -1.59923

N -2.81435 -0.01310 -0.17276

N 0.55729 -1.95580 -3.00791

C 2.44085 2.62123 -1.11347

C 0.44266 3.86724 -1.86538

C -3.92394 -0.59973 -0.82886

C -2.90293 0.39156 1.18609

C 0.72689 -1.81941 -4.38583

C 0.80926 -3.13839 -2.32369

C -4.76559 -1.48262 -0.14476

C -5.84593 -2.05285 -0.80760

C -6.08621 -1.76574 -2.15211

C -5.24074 -0.89022 -2.83061

C -4.16777 -0.29735 -2.17215

C -4.03938 1.05912 1.64873

C -4.10418 1.46084 2.97774

C -3.03872 1.21586 3.84447

C -1.90728 0.55101 3.37607

C -1.83976 0.12673 2.05260

C 1.22904 -3.07661 -0.97878

C 1.46742 -4.25182 -0.29120

C 1.28448 -5.48791 -0.92226

C 0.85342 -5.54606 -2.25197

C 0.61040 -4.38243 -2.95859

C 1.76661 -2.50856 -5.04047

C 1.93817 -2.33307 -6.40248

C 1.09319 -1.47772 -7.11515

C 0.06849 -0.78698 -6.45801

C -0.11877 -0.94705 -5.09743

C -0.46675 3.86915 -2.92663

C -1.11963 5.04770 -3.27749

C -0.85243 6.23122 -2.59357

C 0.06995 6.22704 -1.54626

C 0.71062 5.05180 -1.17286

C 3.37798 3.42113 -1.77428

C 4.71738 3.35861 -1.40771

C 5.13807 2.49141 -0.39899

C 4.20149 1.69277 0.25371

C 2.85454 1.76308 -0.08996

C 2.03977 0.45762 -3.00794

N 3.01278 0.49383 -3.63342

C -1.37359 2.45845 -0.10788

N -1.77346 3.36433 0.49012

H -4.56866 -1.72303 0.89753

H -6.49904 -2.73958 -0.27151

H -6.93137 -2.22005 -2.66606

H -5.42573 -0.65053 -3.87648

H -3.52490 0.40989 -2.69391

H -4.85784 1.27421 0.96543

H -4.98871 1.98562 3.33494

H -3.09156 1.54213 4.88158

H -1.07463 0.34739 4.04735

H -0.96445 -0.40876 1.68906

H 1.39881 -2.11189 -0.50620

H 1.81119 -4.21154 0.74007

H 1.47051 -6.40867 -0.37281

H 0.68442 -6.50901 -2.72902

H 0.22376 -4.41961 -3.97375

H 2.45533 -3.12534 -4.46909

H 2.75181 -2.84558 -6.91102

H 1.23825 -1.34067 -8.18492

H -0.59240 -0.12906 -7.01807

H -0.92689 -0.43299 -4.58228

H -0.66175 2.94960 -3.47569

H -1.83100 5.03994 -4.10190

H -1.35867 7.15324 -2.87383

H 0.28045 7.14576 -1.00099

H 1.40812 5.04580 -0.33833

H 3.05829 4.07913 -2.57907

H 5.44219 3.98267 -1.92820

H 6.18982 2.44097 -0.12301

H 4.51582 1.01960 1.04985

H 2.12102 1.15511 0.43678

$CF_3C(O)O^-$

Charge: -1

Multiplicity: 1

Number of Imaginary Frequencies: 0

Final single point energy -526.504451228985 Eh

O -4.62138 0.23228 -0.01146

C -3.51677 0.79139 -0.03848

O -3.18551 1.98181 -0.03394

C -2.33777 -0.23032 -0.08281

F -2.41939 -1.04800 -1.15305

F -2.33169 -1.03563 1.00035

F -1.11838 0.32964 -0.13571

$CF_3C(O)^•$

Charge: 0

Multiplicity: 2

Number of Imaginary Frequencies: 0

Final single point energy -451.041807194817 Eh

C -6.98544 -0.12462 0.17352

C -5.76758 0.82152 0.08369

F -6.72861 -1.07533 1.05885

F -7.18436 -0.67815 -1.01340

F -8.09134 0.51072 0.53878

O -5.77567 1.97548 0.27717

CO

Charge: 0

Multiplicity: 1

Number of Imaginary Frequencies: 0

Final single point energy -113.353274270031 Eh

C -5.76773 0.84298 0.08729

O -5.77552 1.95402 0.27357

$CF_3^•$

Charge: 0

Multiplicity: 2

Number of Imaginary Frequencies: 0

Final single point energy -337.658290541734 Eh

C -2.26995 -0.29541 -0.08503

F -2.44574 -1.03016 -1.15797

F -2.35762 -1.01738 1.00697

F -1.13393 0.35863 -0.13519

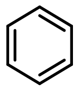

Charge: 0

Multiplicity: 1

Number of Imaginary Frequencies: 0

Final single point energy -232.404069781164 Eh

C -5.60192 0.20850 0.06703

C -4.41554 0.94183 0.04846

C -5.56040 -1.18566 0.05154

C -4.33250 -1.84651 0.01739

C -3.14612 -1.11318 -0.00118

C -3.18763 0.28098 0.01431

H -6.56091 0.72466 0.09354

H -4.44796 2.03066 0.06052

H -2.18713 -1.62934 -0.02769

H -2.26106 0.85373 -0.00009

H -6.48698 -1.75841 0.06594

H -4.30007 -2.93534 0.00532

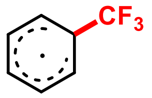

Charge: 0

Multiplicity: 2

Number of Imaginary Frequencies: 0

Final single point energy -570.103923971262 Eh

C -5.94876 -0.48043 0.31832

C -4.98880 0.41732 -0.04943

C -5.63376 -1.93030 0.54387

C -4.19345 -2.27578 0.30188

C -3.27762 -1.33289 -0.06541

C -3.64320 0.02311 -0.25282

H -6.97827 -0.16183 0.47448

H -5.26372 1.46255 -0.18844

H -2.24018 -1.63000 -0.21664

H -2.89607 0.75625 -0.54981

H -3.89677 -3.31359 0.44573

C -6.54650 -2.81553 -0.29874

F -6.36254 -2.62464 -1.60955

F -7.83900 -2.57676 -0.04433

F -6.33446 -4.11523 -0.05723

H -5.90687 -2.20684 1.58097

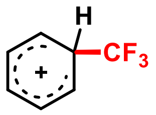

Charge: +1

Multiplicity: 1

Number of Imaginary Frequencies: 0

Final single point energy -569.917260349994 Eh

C -5.95271 -0.48533 0.16604

C -4.97366 0.44951 -0.01604

C -5.60529 -1.90113 0.34857

C -4.19949 -2.27846 0.14895

C -3.24461 -1.31890 -0.03294

C -3.63282 0.03199 -0.11743

H -7.00175 -0.20149 0.23638

H -5.22018 1.50458 -0.10233

H -2.19627 -1.58824 -0.13221

H -2.86276 0.78641 -0.27770

H -3.93876 -3.33425 0.20648

C -6.61609 -2.88365 -0.24825

F -6.58456 -2.84409 -1.57796

F -7.85019 -2.58563 0.14220

F -6.34598 -4.12735 0.13210

H -5.72484 -2.02856 1.45698

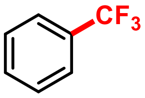

Charge: 0

Multiplicity: 1

Number of Imaginary Frequencies: 0

Final single point energy -569.560708903622 Eh

C -5.90976 -0.49944 -0.05702

C -4.91099 0.46904 -0.01091

C -5.55796 -1.84931 -0.08959

C -4.21687 -2.23440 -0.07601

C -3.22357 -1.26035 -0.02995

C -3.56962 0.08969 0.00207

H -6.95799 -0.20827 -0.06282

H -5.18172 1.52301 0.01787

H -2.17638 -1.55729 -0.01618

H -2.79046 0.84949 0.03994

H -3.95182 -3.28934 -0.09682

C -6.62891 -2.89403 -0.18742

F -6.93333 -3.18310 -1.46355

F -7.76945 -2.50607 0.39619

F -6.26429 -4.04738 0.38609

EtOAc

Charge: 0

Multiplicity: 1

Number of Imaginary Frequencies: 0

Final single point energy -307.855531328024 Eh

C -8.36037 0.29185 0.34410

C -8.19231 -1.02479 -0.37866

H -7.60860 1.00697 -0.01432

H -8.22526 0.16449 1.42570

H -9.35373 0.71755 0.15899

O -9.09561 -2.01987 0.12503

H -7.19490 -1.44416 -0.21196

H -8.35479 -0.91076 -1.45633

C -10.33602 -2.03315 -0.37392

O -10.71571 -1.27378 -1.23056

C -11.16887 -3.09933 0.27121

H -10.66282 -4.06922 0.19874

H -12.14877 -3.14752 -0.20972

H -11.29111 -2.87074 1.33758

EtOAc(H^+)

Charge: +1

Multiplicity: 1

Number of Imaginary Frequencies: 0

Final single point energy -308.259452527708 Eh

C -8.25651 0.28160 0.37974

C -8.16929 -0.93568 -0.49902

H -7.48441 0.98680 0.04840

H -8.06260 0.02718 1.42785

H -9.22914 0.77988 0.29718

O -9.12112 -1.97189 -0.03185

H -7.20877 -1.44674 -0.41618

H -8.39464 -0.73391 -1.54948

C -10.33359 -1.98367 -0.37605

O -10.73958 -1.06494 -1.17971

C -11.22607 -3.02752 0.15500

H -10.65378 -3.76091 0.72535

H -11.75873 -3.50996 -0.67428

H -11.97232 -2.54657 0.80355

H -11.68604 -1.15331 -1.40391

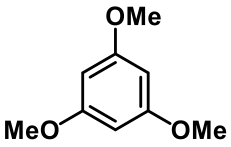

Charge: 0

Multiplicity: 1

Number of Imaginary Frequencies: 0

Final single point energy -576.129819900274 Eh

C -5.77859 1.07312 0.00862

C -5.12988 2.31753 0.02170

C -4.99584 -0.08445 0.00184

C -3.59731 -0.02060 0.00776

C -2.97885 1.23055 0.02067

C -3.73976 2.40382 0.02767

H -3.03796 -0.95043 0.00216

H -6.86043 1.01200 0.00398

O -1.64129 1.39714 0.02731

O -5.79518 3.49050 0.02938

O -5.51782 -1.32775 -0.01088

C -7.20423 3.47982 0.02196

C -0.81496 0.25517 0.01977

C -6.91864 -1.48155 -0.01487

H -7.51910 4.52735 0.02816

H -7.61034 2.97949 0.91276

H -7.60102 2.99284 -0.88036

H 0.21584 0.62119 0.02569

H -0.97469 -0.35296 -0.88206

H -0.97951 -0.36823 0.91026

H -7.10961 -2.55855 -0.02286

H -7.37220 -1.03077 -0.90919

H -7.37593 -1.04330 0.88376

H -3.25074 3.37396 0.03772

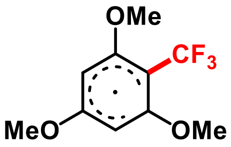

Charge: 0

Multiplicity: 2

Number of Imaginary Frequencies: 0

Final single point energy -913.834056055773 Eh

C -5.49863 1.20962 0.01095

C -4.81547 2.37908 -0.22919

C -4.78483 -0.00109 0.14780

C -3.38420 -0.04153 -0.08816

C -2.68945 1.11113 -0.31738

C -3.31938 2.46443 -0.14872

H -2.90462 -1.01557 -0.13198

H -6.58369 1.21102 0.03782

O -1.39240 1.19664 -0.66285

O -5.37591 3.57158 -0.50713

O -5.36067 -1.19119 0.43407

C -6.78208 3.65261 -0.56704

C -0.66523 -0.00254 -0.81413

C -6.75381 -1.24093 0.62739

H -7.02273 4.68728 -0.82664

H -7.23684 3.40785 0.40354

H -7.18775 2.98064 -1.33720

H 0.34605 0.28339 -1.11629

H -1.11088 -0.64211 -1.58962

H -0.61816 -0.56035 0.13190

H -6.99578 -2.27898 0.87409

H -7.30106 -0.95479 -0.28298

H -7.07054 -0.59253 1.45744

H -2.94088 3.15249 -0.91970

C -2.88958 3.09109 1.18711

F -3.41330 4.30824 1.35945

F -3.28340 2.34336 2.22593

F -1.56269 3.22004 1.27755

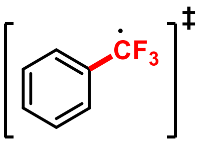

Charge: 0

Multiplicity: 2

Number of Imaginary Frequencies: 1

Final single point energy -570.062384146115 Eh

C -0.81475 0.97229 0.58111

C 0.09573 1.80223 -0.05680

C -0.44804 -0.35218 0.91985

C 0.89954 -0.75288 0.75551

C 1.79966 0.08734 0.11629

C 1.40039 1.36076 -0.30562

H -1.82741 1.31803 0.78278

H -0.20104 2.80823 -0.35088

H 2.82658 -0.23911 -0.04337

H 2.11114 2.01634 -0.80655

H 1.21185 -1.73982 1.09318

C -1.35742 -1.43065 -0.80488

F -2.66597 -1.23858 -0.81192

F -0.82895 -0.94619 -1.91310

F -1.09467 -2.72350 -0.71233

H -1.10665 -0.94230 1.55674

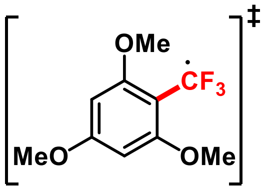

Charge: 0

Multiplicity: 2

Number of Imaginary Frequencies: 1

Final single point energy -913.795051043875 Eh

C -1.28366 -0.08795 -0.15529

C -0.57799 1.04883 -0.55143

C -0.58032 -1.28797 0.01112

C 0.80287 -1.36669 -0.22056

C 1.48692 -0.22456 -0.60988

C 0.82470 1.02341 -0.69318

H 1.29181 -2.32827 -0.10198

H -2.35552 -0.04840 0.00005

O 2.80454 -0.18058 -0.86717

O -1.14599 2.24825 -0.76050

O -1.16605 -2.43882 0.38718

C -2.52593 2.40386 -0.51069

C 3.56887 -1.35282 -0.68373

C -2.55471 -2.45598 0.63620

H -2.76042 3.45049 -0.72378

H -2.76833 2.18598 0.53877

H -3.12893 1.76052 -1.16682

H 4.60123 -1.08849 -0.92916

H 3.23468 -2.15893 -1.35182

H 3.52165 -1.69928 0.35801

H -2.80052 -3.48044 0.92962

H -3.12864 -2.19283 -0.26356

H -2.82490 -1.77302 1.45386

H 1.32574 1.86031 -1.17298

C 1.44646 1.91814 1.30732

F 0.35975 2.24128 2.00030

F 2.13973 1.01850 1.99648

F 2.19298 3.00545 1.14365

12. References

(1) Boronin, E. N.; Svetlakova, M. M.; Vorobyov, I. I.; Malysheva, Y. B.; Polushtaytsev, Y. V; Mensov, S. N.; Vorotyntsev, A. V; Fedorov, A. Y.; Noël, T.; Nyuchev, A. V. *React. Chem. Eng.* **2024**. doi:10.1039/D4RE00130C

(2) Martínez, J.; Cortés, J. F.; Miranda, R. *Processes* **2022**, *10*. doi:10.3390/pr10071274

(3) Fantozzi, N.; Volle, J. N.; Porcheddu, A.; Virieux, D.; García, F.; Colacino, E. *Chem. Soc. Rev.* **2023**, *52*, 6680–6714. doi:10.1039/d2cs00997h

(4) Kayahan, E.; Jacobs, M.; Braeken, L.; Thomassen, L. C. J.; Kuhn, S.; van Gerven, T.; Leblebici, M. E. *Beilstein J. Org. Chem.* **2020**, *16*, 2484–2504

(5) Speckmeier, E.; Fischer, T. G.; Zeitler, K. *J. Am. Chem. Soc.* **2018**, *140*, 15353–15365

(6) Hokamp, T.; Dewanji, A.; Lübbesmeyer, M.; Mück-Lichtenfeld, C.; Würthwein, E.-U.; Studer, A. *Angew. Chemie Int. Ed.* **2017**, *56*, 13275–13278. doi:https://doi.org/10.1002/anie.201706534

(7) Xiong, X.; Tan, F.; Yeung, Y.-Y. *Org. Lett.* **2017**, *19*, 4243–4246. doi:10.1021/acs.orglett.7b01899

(8) El-Deeb, I. Y.; Tian, M.; Funakoshi, T.; Matsubara, R.; Hayashi, M. *European J. Org. Chem.* **2017**, *2017*, 409–413. doi:https://doi.org/10.1002/ejoc.201601362

(9) Kang, Y.-F.; Yan, W.-J.; Zhou, T.-W.; Dai, F.; Li, X.-Z.; Bao, X.-Z.; Du, Y.-T.; Yuan, C.-H.; Wang, H.-B.; Ren, X.-R.; Liu, Q.; Jin, X.-L.; Zhou, B.; Zhang, J. *Chem. – A Eur. J.* **2014**, *20*, 8904–8908. doi:https://doi.org/10.1002/chem.201403024

(10) Beil, S. B.; Müller, T.; Sillart, S. B.; Franzmann, P.; Bomm, A.; Holtkamp, M.; Karst, U.; Schade, W.; Waldvogel, S. R. *Angew. Chemie Int. Ed.* **2018**, *57*, 2450–2454. doi:https://doi.org/10.1002/anie.201712718

(11) He, Z.-T.; Li, H.; Haydl, A. M.; Whiteker, G. T.; Hartwig, J. F. *J. Am. Chem. Soc.* **2018**, *140*, 17197–17202. doi:10.1021/jacs.8b10076

(12) Jud, W.; Maljuric, S.; Kappe, C. O.; Cantillo, D. *Org. Lett.* **2019**, *21*, 7970–7975. doi:10.1021/acs.orglett.9b02948

(13) Hong, J.; Huo, L.; Yang, Y.; Wang, G.; Zheng, C. *ChemistrySelect* **2017**, *2*, 3716–3720. doi:https://doi.org/10.1002/slct.201700789

(14) Knauber, T.; Arikan, F.; Röschenthaler, G.-V.; Gooßen, L. J. *Chem. – A Eur. J.* **2011**, *17*, 2689–2697. doi:https://doi.org/10.1002/chem.201002749

(15) Keaveney, S. T.; Schoenebeck, F. *Angew. Chemie Int. Ed.* **2018**, *57*, 4073–4077. doi:https://doi.org/10.1002/anie.201800644

(16) Wang, X.; Xu, Y.; Mo, F.; Ji, G.; Qiu, D.; Feng, J.; Ye, Y.; Zhang, S.; Zhang, Y.; Wang, J. *J. Am. Chem. Soc.* **2013**, *135*, 10330–10333. doi:10.1021/ja4056239

(17) Lin, X.; Hou, C.; Li, H.; Weng, Z. *Chem. – A Eur. J.* **2016**, *22*, 2075–2084. doi:https://doi.org/10.1002/chem.201504306

(18) Arimori, S.; Shibata, N. *Org. Lett.* **2015**, *17*. doi:10.1021/acs.orglett.5b00164

(19) Ye, Y.; Lee, S. H.; Sanford, M. S. *Org. Lett.* **2011**, *13*, 5464–5467. doi:10.1021/ol202174a

(20) Muralirajan, K.; Kancherla, R.; Bau, J. A.; Taksande, M. R.; Qureshi, M.; Takanabe, K.; Rueping, M. *ACS Catal.* **2021**, *11*, 14772–14780. doi:10.1021/acscatal.1c04053

(21) Ouyang, Y.; Xu, X.-H.; Qing, F.-L. *Angew. Chemie Int. Ed.* **2018**, *57*, 6926–6929. doi:https://doi.org/10.1002/anie.201803566

(22) Beatty, J. W.; Douglas, J. J.; Cole, K. P.; Stephenson, C. R. J. *Nat. Commun.* **2015**, *6*, 7919. doi:10.1038/ncomms8919

(23) Majek, M.; von Wangelin, A. J. *Chem. Commun.* **2013**, *49*, 5507–5509. doi:10.1039/C3CC41867G

(24) Lemmens, V.; Vos, C.; Bugaev, A. L.; Vercammen, J.; Van Velthoven, N.; Gascon, J.; De Vos, D. E. *ACS Appl. Mater. Interfaces* **2022**, *14*, 971–977. doi:10.1021/acsami.1c19655

(25) Kariofillis, S. K.; Jiang, S.; Żurański, A. M.; Gandhi, S. S.; Martinez Alvarado, J. I.; Doyle, A. G. *J. Am. Chem. Soc.* **2022**, *144*, 1045–1055. doi:10.1021/jacs.1c12203

(26) Dolbier Jr, W. R. *Guide to Fluorine NMR for Organic Chemists*; John Wiley & Sons, 2016

(27) Port, V. C.; Zeoly, L. A.; Coelho, F.; Cormanich, R. A. *Phys. Chem. Chem. Phys.* **2021**, *23*, 9080–9088. doi:10.1039/D0CP05887D

(28) Seo, S.; Taylor, J. B.; Greaney, M. F. *Chem. Commun.* **2013**, *49*, 6385–6387. doi:10.1039/C3CC41829D

(29) Wu, X.; Chu, L.; Qing, F.-L. *Tetrahedron Lett.* **2013**, *54*, 249–251. doi:https://doi.org/10.1016/j.tetlet.2012.11.011

(30) Eisenreich, F.; Palmans, A. R. A. *Chem. – A Eur. J.* **2022**, *28*, e202201322. doi:https://doi.org/10.1002/chem.202201322

(31) Natte, K.; Jagadeesh, R. V; He, L.; Rabeah, J.; Chen, J.; Taeschler, C.; Ellinger, S.; Zaragoza, F.; Neumann, H.; Brückner, A.; Beller, M. *Angew. Chemie Int. Ed.* **2016**, *55*, 2782–2786. doi:https://doi.org/10.1002/anie.201511131
(32) Eisenreich, F.; Palmans, A. R. A. *Chem. – A Eur. J.* **2022**, *28*, e202201322. doi:https://doi.org/10.1002/chem.202201322
(33) Zhang, H.-R.; Feng, C.-C.; Chen, N.; Zhang, S.-L. *Angew. Chemie Int. Ed.* **2022**, *61*, e202209029. doi:https://doi.org/10.1002/anie.202209029
(34) Wang, D.; Deng, G.-J.; Chen, S.; Gong, H. *Green Chem.* **2016**, *18*, 5967–5970. doi:10.1039/C6GC02000C
(35) Hu, R.-B.; Lam, Y.-P.; Ng, W.-H.; Wong, C.-Y.; Yeung, Y.-Y. *ACS Catal.* **2021**, *11*, 3498–3506. doi:10.1021/acscatal.0c05684
(36) Hu, W.-Q.; Pan, S.; Xu, X.-H.; Vicic, D. A.; Qing, F.-L. *Angew. Chemie Int. Ed.* **2020**, *59*, 16076–16082. doi:https://doi.org/10.1002/anie.202004116
(37) Khan, B. A.; Buba, A. E.; Gooßen, L. J. *Chem. – A Eur. J.* **2012**, *18*, 1577–1581. doi:https://doi.org/10.1002/chem.201102652
(38) Straathof, N. J. W.; Gemoets, H. P. L.; Wang, X.; Schouten, J. C.; Hessel, V.; Noël, T. *ChemSusChem* **2014**, *7*, 1612–1617. doi:https://doi.org/10.1002/cssc.201301282
(39) Singh, K.; Singh, R.; Hazari, A. S.; Adhikari, D. *Chem. Commun.* **2022**, *58*, 4384–4387. doi:10.1039/D2CC00397J
(40) Xiao, F.; Lin, J.-H.; Hao, F.; Zheng, X.; Guo, Y.; Xiao, J.-C. *Org. Chem. Front.* **2022**, *9*, 1982–1985. doi:10.1039/D2QO00067A
(41) Liang, Y.; Taya, A.; Zhao, Z.; Saito, N.; Shibata, N. *Beilstein J. Org. Chem.* **2020**, *16*, 3052–3058. doi:10.3762/bjoc.16.254
(42) Trofymchuk, S.; Bugera, M. Y.; Klipkov, A. A.; Razhyk, B.; Semenov, S.; Tarasenko, K.; Starova, V. S.; Zaporozhets, O. A.; Tananaiko, O. Y.; Alekseenko, A. N.; Pustovit, Y.; Kiriakov, O.; Gerus, I. I.; Tolmachev, A. A.; Mykhailiuk, P. K. *J. Org. Chem.* **2020**, *85*, 3110–3124. doi:10.1021/acs.joc.9b03011
(43) Chang, B.; Shao, H.; Yan, P.; Qiu, W.; Weng, Z.; Yuan, R. *ACS Sustain. Chem. Eng.* **2017**, *5*, 334–341. doi:10.1021/acssuschemeng.6b01682
(44) Yang, J.-Y.; Xu, X.-H.; Qing, F.-L. *J. Fluor. Chem.* **2016**, *186*, 45–51. doi:https://doi.org/10.1016/j.jfluchem.2016.04.008
(45) Angeli, C.; Bories, B.; Cavallini, A.; Cimiraglia, R. *J. Chem. Phys.* **2006**, *124*, 54108. doi:10.1063/1.2148946
(46) Neese, F. *Wiley Interdiscip. Rev. Comput. Mol. Sci.* **2022**, *12*, 1–15. doi:10.1002/wcms.1606
(47) Neese, F. *J. Comput. Chem.* **2003**, *24*, 1740–1747. doi:https://doi.org/10.1002/jcc.10318
(48) Neese, F.; Wennmohs, F.; Hansen, A.; Becker, U. *Chem. Phys.* **2009**, *356*, 98–109. doi:https://doi.org/10.1016/j.chemphys.2008.10.036
(49) Bykov, D.; Petrenko, T.; Izsák, R.; Kossmann, S.; Becker, U.; Valeev, E.; Neese, F. *Mol. Phys.* **2015**, *113*, 1961–1977. doi:10.1080/00268976.2015.1025114
(50) Garcia-Ratés, M.; Neese, F. *J. Comput. Chem.* **2019**, *40*, 1816–1828. doi:https://doi.org/10.1002/jcc.25833
(51) Garcia-Ratés, M.; Neese, F. *J. Comput. Chem.* **2020**, *41*, 922–939. doi:https://doi.org/10.1002/jcc.26139
(52) Helmich-Paris, B.; de Souza, B.; Neese, F.; Izsák, R. *J. Chem. Phys.* **2021**, *155*, 104109. doi:10.1063/5.0058766
